# Supplementary material for: EpiLPS: A fast and flexible Bayesian tool for estimation of the time-varying reproduction number
Source: PLoS Comput Biol. 2022 Oct 10;18(10):e1010618. doi: 10.1371/journal.pcbi.1010618 (PMC9584461; doi:10.1371/journal.pcbi.1010618)
Supplement: S1 Appendix — Analytical gradient for the Langevin-Hastings proposal and analytical version of the ratio of proposal distributions in the LPSMALA algorithm. (PDF) [file pcbi.1010618.s001.pdf]

EpiLPS: A Fast and Flexible Bayesian Tool for Estimation of the Time-Varying Reproduction Number (Supplementary Information 1)

Oswaldo Gressani[*], Jacco Wallinga, Christian L. Althaus, Niel Hens, Christel Faes

Analytical gradient for the Langevin-Hastings proposal

In this appendix, we derive the analytical expression of the gradient:

$$\nabla_{\widetilde{\boldsymbol{\zeta}}} \log p(\widetilde{\boldsymbol{\zeta}}|\lambda, \delta, \mathcal{D}) = \left(\nabla_{\boldsymbol{\theta}}^{\top} \log p(\widetilde{\boldsymbol{\zeta}}|\lambda, \delta, \mathcal{D}), \frac{\partial \log p(\widetilde{\boldsymbol{\zeta}}|\lambda, \delta, \mathcal{D})}{\partial w} \right)^{\top},$$

where the target function is $\log p(\widetilde{\boldsymbol{\zeta}}|\lambda, \delta, \mathcal{D}) = \ell(\widetilde{\boldsymbol{\zeta}}; \mathcal{D}) - 0.5\lambda \boldsymbol{\theta}^{\top} P \boldsymbol{\theta} - b_{\rho} \exp(w) + a_{\rho} w$. Let us first concentrate on the partial derivatives with respect to the spline components:

$$\nabla_{\boldsymbol{\theta}} \log p(\widetilde{\boldsymbol{\zeta}}|\lambda, \delta, \mathcal{D}) = \nabla_{\boldsymbol{\theta}} \ell(\widetilde{\boldsymbol{\zeta}}; \mathcal{D}) - \lambda P \boldsymbol{\theta}. \tag{1}$$

As already shown in the manuscript, the gradient for the log-likelihood $\nabla_{\boldsymbol{\theta}} \ell(\widetilde{\boldsymbol{\zeta}}; \mathcal{D})$ is:

$$\frac{\partial \ell(\widetilde{\boldsymbol{\zeta}}; \mathcal{D})}{\partial \theta_k} = \sum_{t=1}^{T} y_t b_k(t) - \sum_{t=1}^{T} \frac{(y_t + \exp(w)) \exp(\boldsymbol{\theta}^{\top} b(t))}{\big(\exp(\boldsymbol{\theta}^{\top} b(t)) + \exp(w)\big)} b_k(t), \quad k = 1, \ldots, K. \tag{2}$$

[*]Corresponding author. E-mail address: *oswaldo.gressani@uhasselt.be*

The last term to be computed to recover the full gradient is:

$$\frac{\partial \log p(\widetilde{\boldsymbol{\zeta}}|\lambda, \delta, \mathcal{D})}{\partial w} = \frac{\partial \ell(\widetilde{\boldsymbol{\zeta}}; \mathcal{D})}{\partial w} - b_\rho \exp(w) + a_\rho, \tag{3}$$

where

$$\begin{aligned}\frac{\partial \ell(\widetilde{\boldsymbol{\zeta}}; \mathcal{D})}{\partial w} \quad = \quad & \sum_{t=1}^{T} \Bigg\{ \underbrace{\frac{\partial \log \Gamma(y_t + \exp(w))}{\partial w}}_{\text{Term I}} - \underbrace{\frac{\partial \log \Gamma(\exp(w))}{\partial w}}_{\text{Term II}} + \underbrace{\frac{\partial \exp(w) w}{\partial w}}_{\text{Term III}} \\ & - \underbrace{\frac{\partial}{\partial w}(y_t + \exp(w)) \log \Big(\exp(\boldsymbol{\theta}^\top b(t)) + \exp(w) \Big)}_{\text{Term IV}} \Bigg\}. \end{aligned} \tag{4}$$

For Term I, using the chain rule, one recovers:

$$\begin{aligned}\frac{\partial \log \Gamma(y_t + \exp(w))}{\partial w} &= \frac{\partial \log \Gamma(y_t + \exp(w))}{\partial \Gamma(y_t + \exp(w))} \frac{\partial \Gamma(y_t + \exp(w))}{\partial (y_t + \exp(w))} \frac{\partial (y_t + \exp(w))}{\partial w} \\ &= \frac{\Gamma'(y_t + \exp(w))}{\Gamma(y_t + \exp(w))} \exp(w) \\ &= \psi(y_t + \exp(w)) \exp(w), \end{aligned} \tag{5}$$

where $\psi(\cdot)$ is the digamma function. Using the same chain rule argument, we can easily show that for Term II:

$$\frac{\partial \log \Gamma(\exp(w))}{\partial w} = \psi(\exp(w)) \exp(w). \tag{6}$$

Term III is also trivial:

$$\frac{\partial \exp(w) w}{\partial w} = \exp(w)(1 + w). \tag{7}$$

Term IV is as follows:

$$\begin{aligned}\frac{\partial}{\partial w}(y_t+\exp(w))\log\Big(\exp(\boldsymbol{\theta}^\top b(t))+\exp(w)\Big) &= \exp(w)\log\Big(\exp(\boldsymbol{\theta}^\top b(t))+\exp(w)\Big)\\ &\quad +(y_t+\exp(w))\exp(w)\Big(\exp(\boldsymbol{\theta}^\top b(t))+\exp(w)\Big)^{-1}\\ &= \exp(w)\Bigg\{\log\Big(\exp(\boldsymbol{\theta}^\top b(t))+\exp(w)\Big)\\ &\quad +(y_t+\exp(w))\Big(\exp(\boldsymbol{\theta}^\top b(t))+\exp(w)\Big)^{-1}\Bigg\}. \quad (8)\end{aligned}$$

Gathering all the above intermediate results, we obtain the following derivative for (3):

$$\begin{aligned}\frac{\partial \log p(\widetilde{\boldsymbol{\zeta}}|\lambda,\delta,\mathcal{D})}{\partial w} &= \sum_{t=1}^{T}\Bigg\{\exp(w)\Bigg[\psi(y_t+\exp(w))-\psi(\exp(w))+(1+w)-\log\Big(\exp(\boldsymbol{\theta}^\top b(t))+\exp(w)\Big)\\ &\quad -(y_t+\exp(w))\Big(\exp(\boldsymbol{\theta}^\top b(t))+\exp(w)\Big)^{-1}\Bigg]\Bigg\}-b_\rho\exp(w)+a_\rho. \quad (9)\end{aligned}$$

Analytical version of the ratio of proposal distributions in the LPS-MALA algorithm

To determine the analytical form of the ratio of proposal distributions in the Metropolis-within-Gibbs algorithm, let us use the compact notation $\boldsymbol{\Upsilon}_{(m-1)}=\nabla_{\widetilde{\boldsymbol{\zeta}}}\log p(\widetilde{\boldsymbol{\zeta}}|\lambda,\delta,\mathcal{D})\big|_{\widetilde{\boldsymbol{\zeta}}=\widetilde{\boldsymbol{\zeta}}^{(m-1)}}$, $\boldsymbol{\Upsilon}_{(\mathrm{prop})}=\nabla_{\widetilde{\boldsymbol{\zeta}}}\log p(\widetilde{\boldsymbol{\zeta}}|\lambda,\delta,\mathcal{D})\big|_{\widetilde{\boldsymbol{\zeta}}=\widetilde{\boldsymbol{\zeta}}^{(\mathrm{prop})}}$ and define the difference $\Delta\widetilde{\boldsymbol{\zeta}}=\widetilde{\boldsymbol{\zeta}}^{(\mathrm{prop})}-\widetilde{\boldsymbol{\zeta}}^{(m-1)}$, so that:

$$\begin{aligned}q\left(\widetilde{\boldsymbol{\zeta}}^{(\mathrm{prop})},\widetilde{\boldsymbol{\zeta}}^{(m-1)}\right) &= (2\pi)^{-\frac{(K+1)}{2}}|\varrho\Sigma_{LH}|^{-\frac{1}{2}}\exp\Bigg\{-\frac{1}{2\varrho}\left(\widetilde{\boldsymbol{\zeta}}^{(m-1)}-\widetilde{\boldsymbol{\zeta}}^{(\mathrm{prop})}-0.5\varrho\Sigma_{LH}\boldsymbol{\Upsilon}_{(\mathrm{prop})}\right)^\top\Sigma_{LH}^{-1}\\ &\quad\times\left(\widetilde{\boldsymbol{\zeta}}^{(m-1)}-\widetilde{\boldsymbol{\zeta}}^{(\mathrm{prop})}-0.5\varrho\Sigma_{LH}\boldsymbol{\Upsilon}_{(\mathrm{prop})}\right)\Bigg\}. \quad (10)\end{aligned}$$

The kernel of (10) is thus:

$$\begin{aligned}\ker\left(q\left(\widetilde{\boldsymbol{\zeta}}^{(\mathrm{prop})},\widetilde{\boldsymbol{\zeta}}^{(m-1)}\right)\right) &= \exp\Bigg\{-\frac{1}{2\varrho}\left(-(\Delta\widetilde{\boldsymbol{\zeta}})-0.5\varrho\Sigma_{LH}\boldsymbol{\Upsilon}_{(\mathrm{prop})}\right)^\top\Sigma_{LH}^{-1}\\ &\quad\times\left(-(\Delta\widetilde{\boldsymbol{\zeta}})-0.5\varrho\Sigma_{LH}\boldsymbol{\Upsilon}_{(\mathrm{prop})}\right)\Bigg\}\end{aligned}$$

$$
\begin{aligned}
&= \exp\Big\{-\frac{1}{2\varrho}\Big[\Big(-(\Delta\widetilde{\boldsymbol{\zeta}})^\top\Sigma_{LH}^{-1} - 0.5\varrho\boldsymbol{\Upsilon}_{(\text{prop})}^\top\Sigma_{LH}\Sigma_{LH}^{-1}\Big) \\
&\quad \times\Big(-(\Delta\widetilde{\boldsymbol{\zeta}}) - 0.5\varrho\Sigma_{LH}\boldsymbol{\Upsilon}_{(\text{prop})}\Big)\Big]\Big\} \\
&= \exp\Big\{-\frac{1}{2\varrho}\Big[(\Delta\widetilde{\boldsymbol{\zeta}})^\top\Sigma_{LH}^{-1}(\Delta\widetilde{\boldsymbol{\zeta}}) + \varrho\boldsymbol{\Upsilon}_{(\text{prop})}^\top(\Delta\widetilde{\boldsymbol{\zeta}}) + \frac{\varrho^2}{4}\boldsymbol{\Upsilon}_{(\text{prop})}^\top\Sigma_{LH}\boldsymbol{\Upsilon}_{(\text{prop})}\Big]\Big\} \\
&= \exp\Big\{-\frac{1}{2\varrho}(\Delta\widetilde{\boldsymbol{\zeta}})^\top\Sigma_{LH}^{-1}(\Delta\widetilde{\boldsymbol{\zeta}}) - \frac{1}{2}\boldsymbol{\Upsilon}_{(\text{prop})}^\top(\Delta\widetilde{\boldsymbol{\zeta}}) - \frac{\varrho}{8}\boldsymbol{\Upsilon}_{(\text{prop})}^\top\Sigma_{LH}\boldsymbol{\Upsilon}_{(\text{prop})}\Big\}.
\end{aligned}
$$

Using a similar argument, we can show that the kernel of $q\left(\widetilde{\boldsymbol{\zeta}}^{(m-1)}, \widetilde{\boldsymbol{\zeta}}^{(\text{prop})}\right)$ is:

$$
\ker\left(q\left(\widetilde{\boldsymbol{\zeta}}^{(m-1)}, \widetilde{\boldsymbol{\zeta}}^{(\text{prop})}\right)\right) = \exp\Big\{-\Big[\frac{1}{2\varrho}(\Delta\widetilde{\boldsymbol{\zeta}})^\top\Sigma_{LH}^{-1}(\Delta\widetilde{\boldsymbol{\zeta}}) - \frac{1}{2}\boldsymbol{\Upsilon}_{(m-1)}^\top(\Delta\widetilde{\boldsymbol{\zeta}}) + \frac{\varrho}{8}\boldsymbol{\Upsilon}_{(m-1)}^\top\Sigma_{LH}\boldsymbol{\Upsilon}_{(m-1)}\Big]\Big\}.
$$

This can be used to compute the ratio:

$$
\begin{aligned}
\frac{q\left(\widetilde{\boldsymbol{\zeta}}^{(\text{prop})}, \widetilde{\boldsymbol{\zeta}}^{(m-1)}\right)}{q\left(\widetilde{\boldsymbol{\zeta}}^{(m-1)}, \widetilde{\boldsymbol{\zeta}}^{(\text{prop})}\right)} &= \ker\left(q\left(\widetilde{\boldsymbol{\zeta}}^{(\text{prop})}, \widetilde{\boldsymbol{\zeta}}^{(m-1)}\right)\right)\ker^{-1}\left(q\left(\widetilde{\boldsymbol{\zeta}}^{(m-1)}, \widetilde{\boldsymbol{\zeta}}^{(\text{prop})}\right)\right) \\
&= \exp\Big\{-\frac{1}{2}\boldsymbol{\Upsilon}_{(\text{prop})}^\top(\Delta\widetilde{\boldsymbol{\zeta}}) - \frac{\varrho}{8}\boldsymbol{\Upsilon}_{(\text{prop})}^\top\Sigma_{LH}\boldsymbol{\Upsilon}_{(\text{prop})} - \frac{1}{2}\boldsymbol{\Upsilon}_{(m-1)}^\top(\Delta\widetilde{\boldsymbol{\zeta}}) + \frac{\varrho}{8}\boldsymbol{\Upsilon}_{(m-1)}^\top\Sigma_{LH}\boldsymbol{\Upsilon}_{(m-1)}\Big\} \\
&= \exp\Big\{-\frac{1}{2}\left(\boldsymbol{\Upsilon}_{(\text{prop})} + \boldsymbol{\Upsilon}_{(m-1)}\right)^\top(\Delta\widetilde{\boldsymbol{\zeta}}) - \frac{\varrho}{8}\Big[\left(\boldsymbol{\Upsilon}_{(\text{prop})} + \boldsymbol{\Upsilon}_{(m-1)}\right)^\top\Sigma_{LH}\left(\boldsymbol{\Upsilon}_{(\text{prop})} - \boldsymbol{\Upsilon}_{(m-1)}\right)\Big]\Big\} \\
&= \exp\Big\{-\frac{1}{2}\left(\boldsymbol{\Upsilon}_{(\text{prop})} + \boldsymbol{\Upsilon}_{(m-1)}\right)^\top\Big(\Delta\widetilde{\boldsymbol{\zeta}} + \frac{\varrho\Sigma_{LH}}{4}\left(\boldsymbol{\Upsilon}_{(\text{prop})} - \boldsymbol{\Upsilon}_{(m-1)}\right)\Big)\Big\}.
\end{aligned}
$$
