# Supplementary material for: EpiLPS: A fast and flexible Bayesian tool for estimation of the time-varying reproduction number
Source: PLoS Comput Biol. 2022 Oct 10;18(10):e1010618. doi: 10.1371/journal.pcbi.1010618 (PMC9584461; doi:10.1371/journal.pcbi.1010618)
Supplement: S2 Appendix — Complete simulation results (for EpiLPS and EpiEstim) when EpiEstim reports Rt at the window boundary, sensitivity analyses and computational time of EpiLPS. (PDF) [file pcbi.1010618.s002.pdf]

EpiLPS: A Fast and Flexible Bayesian Tool for Estimation of the Time-Varying Reproduction Number (Supplementary Information 2)

Oswaldo Gressani, Jacco Wallinga, Christian L. Althaus, Niel Hens, Christel Faes

Table of Contents

1. Data generating process **2**

2. Specification of the simulation settings **2**

3. Scaling of the covariance matrix in LPSMAP **3**

4. Figures of the simulation study **4**

Scenario 1 4

Scenario 2 5

Scenario 3 6

Scenario 4 7

Scenario 5 8

Scenario 6 9

Scenario 7 10

Scenario 8 11

Scenario 9 12

5. Plot of the mean and variance of y_t in Scenarios 1-9 .... **13**

6. Sensitivity analyses **17**

Sensitivity with respect to prior choice of δ 17

Sensitivity with respect to the number of B-splines K 19

Sensitivity with respect to the B-splines domain $[1, T]$ 20

7. Computational time **21**

1. Data generating process

The data generating process (DGP) to obtain the incidence of cases $\{y_t, t = 1, \dots, T\}$ for an epidemic lasting T days given a serial interval distribution $\boldsymbol{\varphi} = \{\varphi_1, \dots, \varphi_k\}$ of length k and a functional form of the reproduction number denoted by $R(t)$ is as follows. (Note: we use the notation $R(t)$ or R_t interchangeably here). From the renewal equation model, the expected number of cases $E(y_t) = \mu(t)$ is related to the reproduction number in the following way:

$$\mu(t) = \begin{cases} \mu(1) & \text{for } t = 1, \\ R(t)\left(\sum_{s=1}^{t-1} \varphi_s y_{t-s}\right) & \text{for } 2 \leq t \leq k, \\ R(t)\left(\sum_{s=1}^{k} \varphi_s y_{t-s}\right) & \text{for } k < t \leq T. \end{cases}$$

On the first day of the simulated epidemic, the DGP assumes a mean incidence of $\mu(1) = 10$ and starts with $y_1 = 10$ index cases. The mean number of cases on day $t = 2$ is then computed using the above renewal equation formula and the number of cases y_2 is sampled from the negative binomial distribution with mean $\mu(2)$ and overdispersion parameter ρ. This process is iterated until day T as illustrated below.

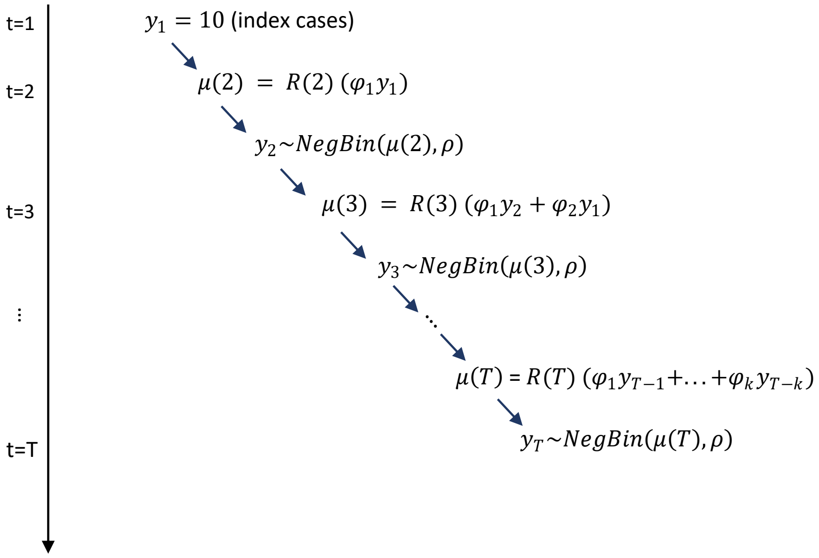

2. Specification of the simulation settings

Scenario(s)	Disease	SI Mean and SD*	Reference
1-4	Influenza	Mean = 2.6<br>SD = 1.5	Ferguson et al. (2005) [1]<br>Cori et al. (2013) [2]
5-8	SARS-CoV-1	Mean = 8.4<br>SD = 3.8	Lipsitch et al. (2003) [3]<br>Cori et al. (2013) [2]
9	MERS-CoV	Mean = 6.8<br>SD = 4.1	Cauchemez et al. (2016) [4]

***S2 Table 1.* * Serial interval distributions used in the different simulation scenarios. Discretized version of the serial interval is obtained by using the Cori et al. (2013) discretization formula assuming a (shifted) Gamma distribution (see the discr_si routine in the EpiEstim package).**

Scenario	$R(t)$ functional form	Days	Overdispersion
1	$R(t) = 1.3$	$T = 40$	$\rho = 1000$
2	$R(t) = 2$ if $t < 20$<br>$R(t) = 0.9$ if $t \geq 20$	$T = 40$	$\rho = 1000$
3	$R(t) = 0.25 + exp(cos(t/7))$	$T = 40$	$\rho = 1000$
4	$R(t) = exp(cos(t/15))$	$T = 40$	$\rho = 1000$
5	$R(t) = 1.3$	$T = 40$	$\rho = 5$
6	$R(t) = 2$ if $t < 20$<br>$R(t) = 0.9\ if\ t \geq 20$	$T = 40$	$\rho = 5$
7	$R(t) = 0.25 + exp(cos(t/7))$	$T = 40$	$\rho = 5$
8	$R(t) = exp\ (cos(t/15))$	$T = 40$	$\rho = 5$
9	$R(t) = 0.5\ (exp(sin(\pi t/9)) + 1.5\ exp(cos(4/t)))$	$T = 60$	$\rho = 50$

S2 Table 2. Functional form of the reproduction number; total number of days and overdispersion parameter in each scenario.

For a simulation scenario with S replications of epidemics with a duration of T days, the formulas for the bias, mean square error (MSE), coverage probability (CP) and credible interval width (CI^{Δ}) averaged over days 8,…,T are provided below. The subscript $\mathcal{M}$ indicates for which model the performance measure is computed with $\mathcal{M} \in (\text{LPSMAP}, \text{LPSMALA}, \text{EpiEstim})$, $\hat{R}$ denotes the estimated reproduction number, $\mathbb{I}(\cdot)$ is the indicator function and R is the true value.

$$\text{Bias}_{\mathcal{M}} = \frac{1}{(T-8+1)} \sum_{t=8}^{T} \left\{ \frac{1}{S} \sum_{s=1}^{S} \left(\widehat{R}_{\mathcal{M},t}^{(s)} - R_t \right) \right\},$$

$$\text{MSE}_{\mathcal{M}} = \frac{1}{(T-8+1)} \sum_{t=8}^{T} \left\{ \frac{1}{S} \sum_{s=1}^{S} \left(\widehat{R}_{\mathcal{M},t}^{(s)} - R_t \right)^2 \right\},$$

$$\text{CP}_{\mathcal{M},(1-\alpha)\%} = \frac{1}{(T-8+1)} \sum_{t=8}^{T} \left\{ \left(\frac{1}{S} \sum_{s=1}^{S} \mathbb{I}\left(R_t \in \text{CI}_{\mathcal{M},t,(1-\alpha)\%}^{(s)} \right) \right) \times 100 \right\},$$

$$\text{CI}_{\mathcal{M},(1-\alpha)\%}^{\Delta} = \frac{1}{(T-8+1)} \sum_{t=8}^{T} \left\{ \frac{1}{S} \sum_{s=1}^{S} \text{CI}_{\mathcal{M},t,(1-\alpha)\%}^{\Delta,(s)} \right\}.$$

3. Scaling of the covariance matrix in LPSMAP

The covariance matrix scaling factor $0 < \kappa_t^{\hat{\rho}} < 1$ proposed in LPSMAP is used to improve the accuracy of the (approximate) credible interval for the reproduction number and is based on the mean-to-variance (MV) ratio $E(y_t)/V(y_t)$ as the latter gives an indication of how much signal in the data is blurred by the noise. An estimate of the MV ratio for the whole epidemic period is $\widehat{MV} = (1 + (1/\hat{\rho})(1/T)\sum_{t=1}^{T} \hat{\mu}(t))^{-1}$ and can be interpreted as a measure of the global signal strength in the data. We recommend using the scaling factor correction on the covariance matrix whenever the following inequality holds $\widehat{MV} \geq (1/\hat{\rho})$. As data are more overdispersed, i.e. as $\rho \to 0$, we have that $\widehat{MV} \to 0$ and $(1/\hat{\rho}) \to +\infty$, so that the latter inequality is less likely to hold and the scaling correction will not be implemented.

4. Figures of the simulation study

Scenario 1

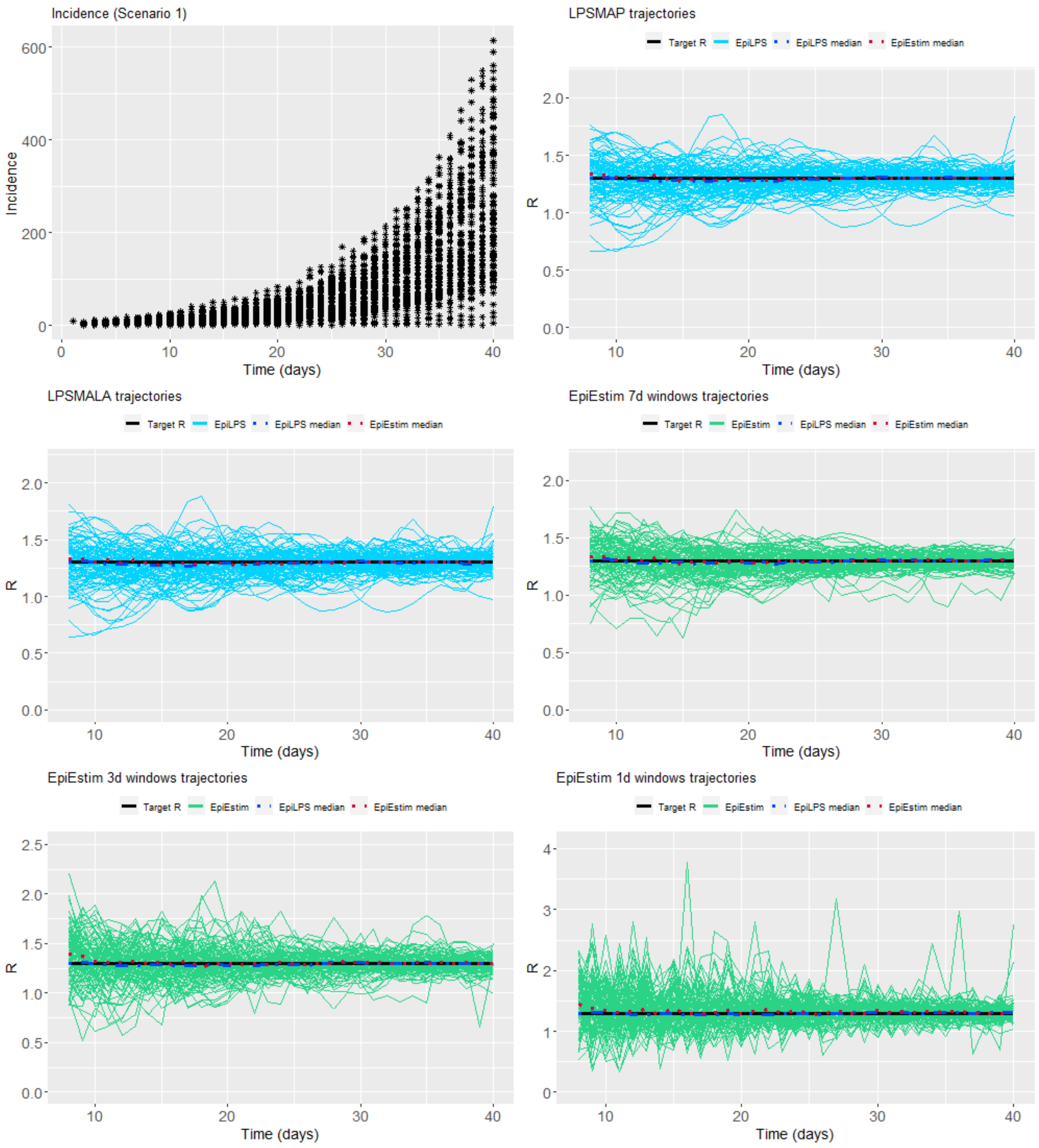

S2 Fig 1. ***Results for Scenario 1 considering S=100 simulated epidemics with a duration of T=40 days and a FLU like serial interval. Top left panel represents the simulated epidemic curves. Estimated trajectories in light blue are for EpiLPS with LPSMAP and LPSMALA (with a chain length of 3 000 including a burn-in of 1 000) respectively, using K=40 B-splines and a second-order penalty. The green estimated trajectories are for EpiEstim under different sliding windows (weekly 7d, three days 3d and daily 1d) and estimated R(t) reported at the end of the window. Dashed (dotted) curves correspond to the pointwise median estimate of R(t) with EpiLPS (EpiEstim).***

Scenario 2

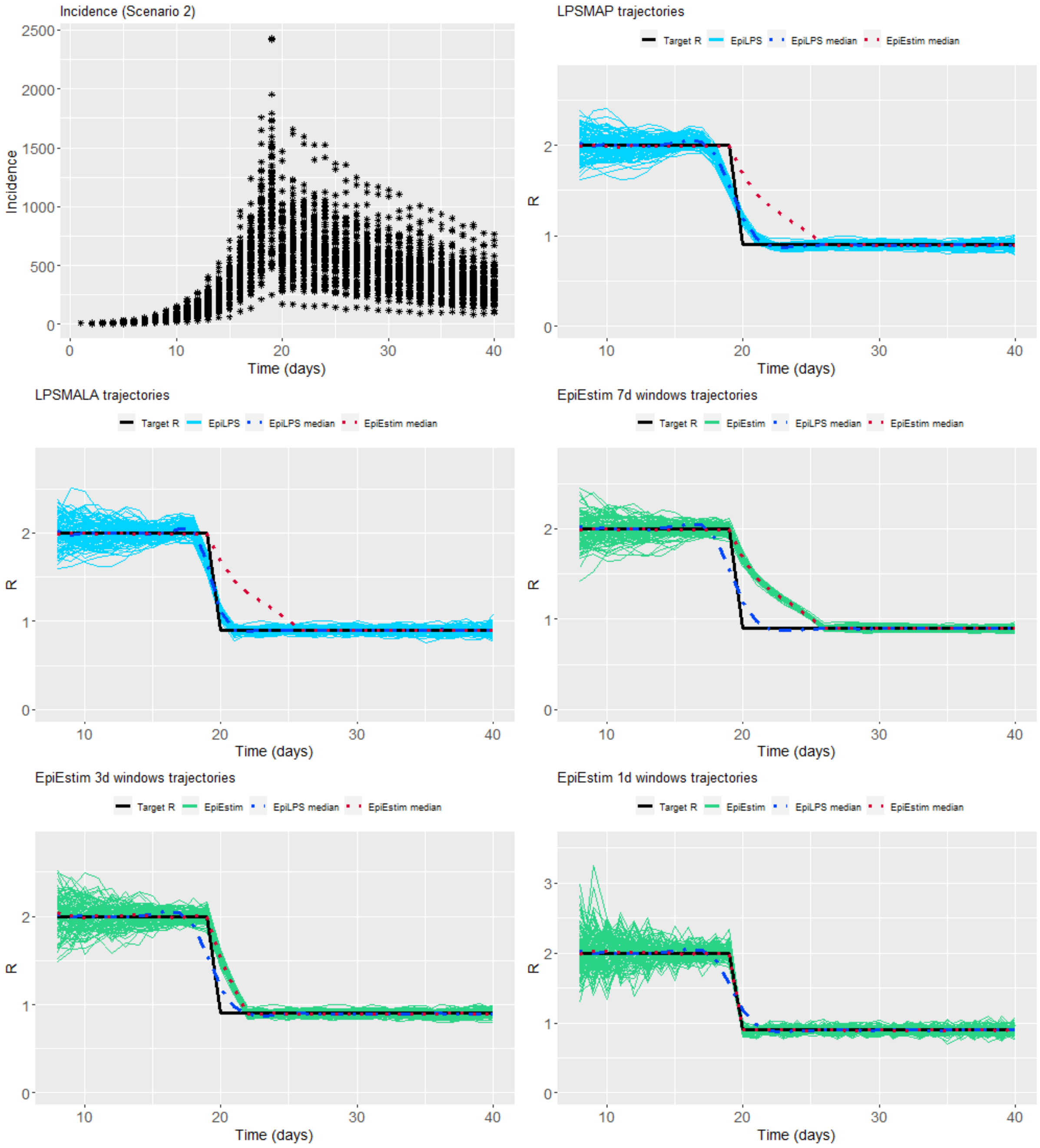

S2 Fig 2. ***Results for Scenario 2 considering S=100 simulated epidemics with a duration of T=40 days and a FLU like serial interval. Top left panel represents the simulated epidemic curves. Estimated trajectories in light blue are for EpiLPS with LPSMAP and LPSMALA (with a chain length of 3 000 including a burn-in of 1 000) respectively, using K=40 B-splines and a second-order penalty. The green estimated trajectories are for EpiEstim under different sliding windows (weekly 7d, three days 3d and daily 1d) and estimated R(t) reported at the end of the window. Dashed (dotted) curves correspond to the pointwise median estimate of R(t) with EpiLPS (EpiEstim).***

Scenario 3

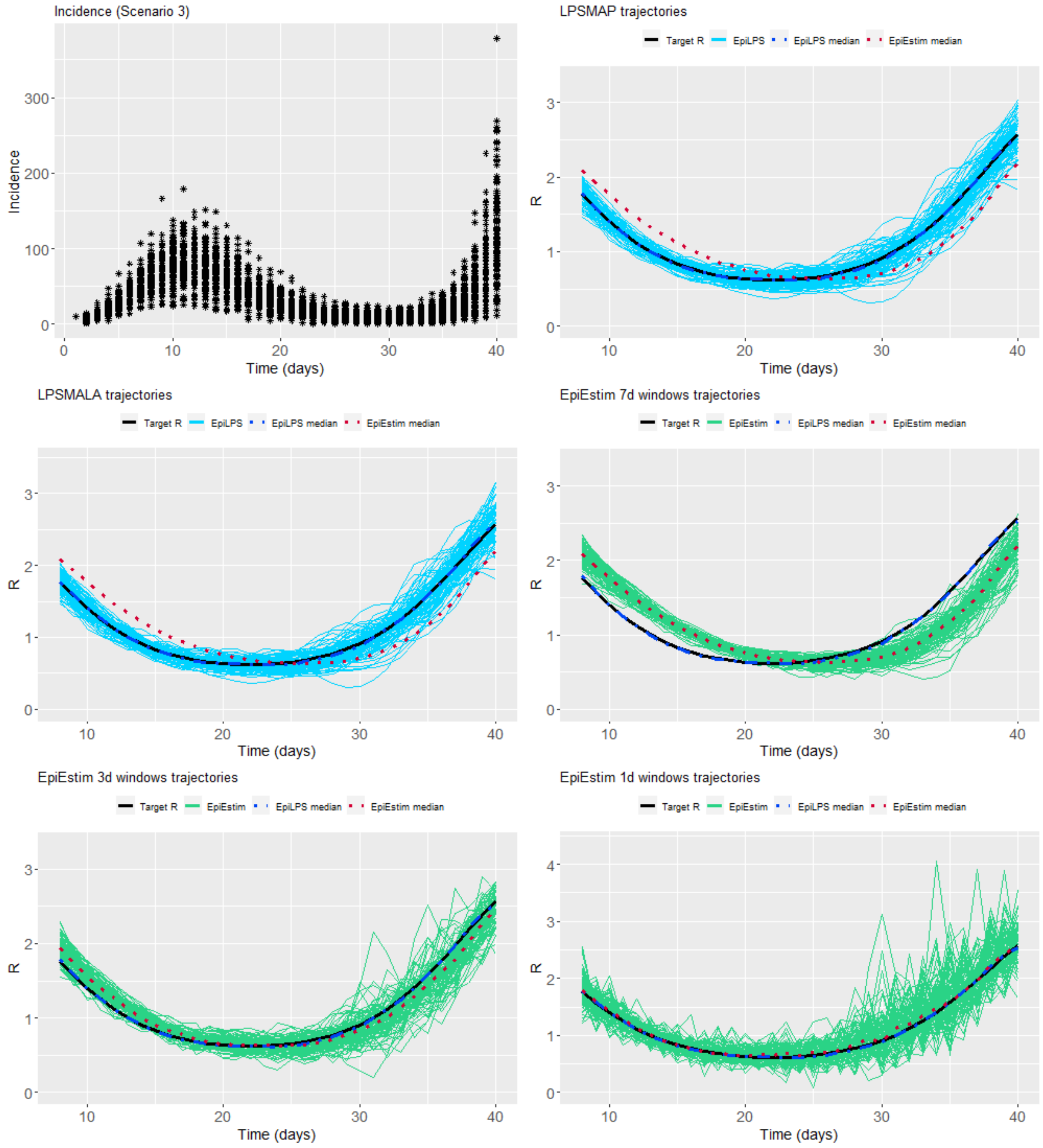

S2 Fig 3. Results for Scenario 3 considering S=100 simulated epidemics with a duration of T=40 days and a FLU like serial interval. Top left panel represents the simulated epidemic curves. Estimated trajectories in light blue are for EpiLPS with LPSMAP and LPSMALA (with a chain length of 3 000 including a burn-in of 1 000) respectively, using K=40 B-splines and a second-order penalty. The green estimated trajectories are for EpiEstim under different sliding windows (weekly 7d, three days 3d and daily 1d) and estimated R(t) reported at the end of the window. Dashed (dotted) curves correspond to the pointwise median estimate of R(t) with EpiLPS (EpiEstim).

Scenario 4

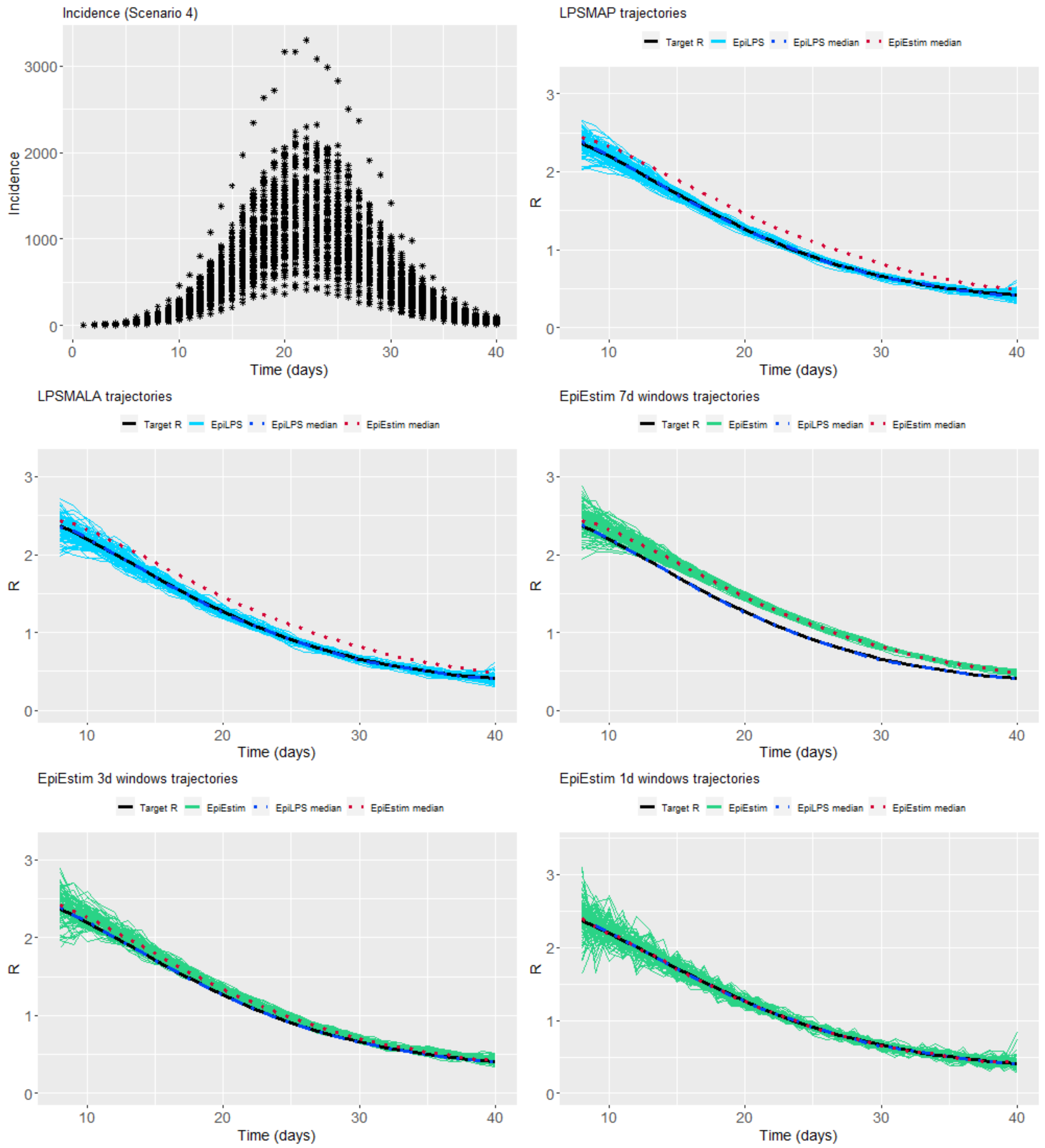

S2 Fig 4. ***Results for Scenario 4 considering S=100 simulated epidemics with a duration of T=40 days and a FLU like serial interval. Top left panel represents the simulated epidemic curves. Estimated trajectories in light blue are for EpiLPS with LPSMAP and LPSMALA (with a chain length of 3 000 including a burn-in of 1 000) respectively, using K=40 B-splines and a second-order penalty. The green estimated trajectories are for EpiEstim under different sliding windows (weekly 7d, three days 3d and daily 1d) and estimated R(t) reported at the end of the window. Dashed (dotted) curves correspond to the pointwise median estimate of R(t) with EpiLPS (EpiEstim).***

Scenario 5

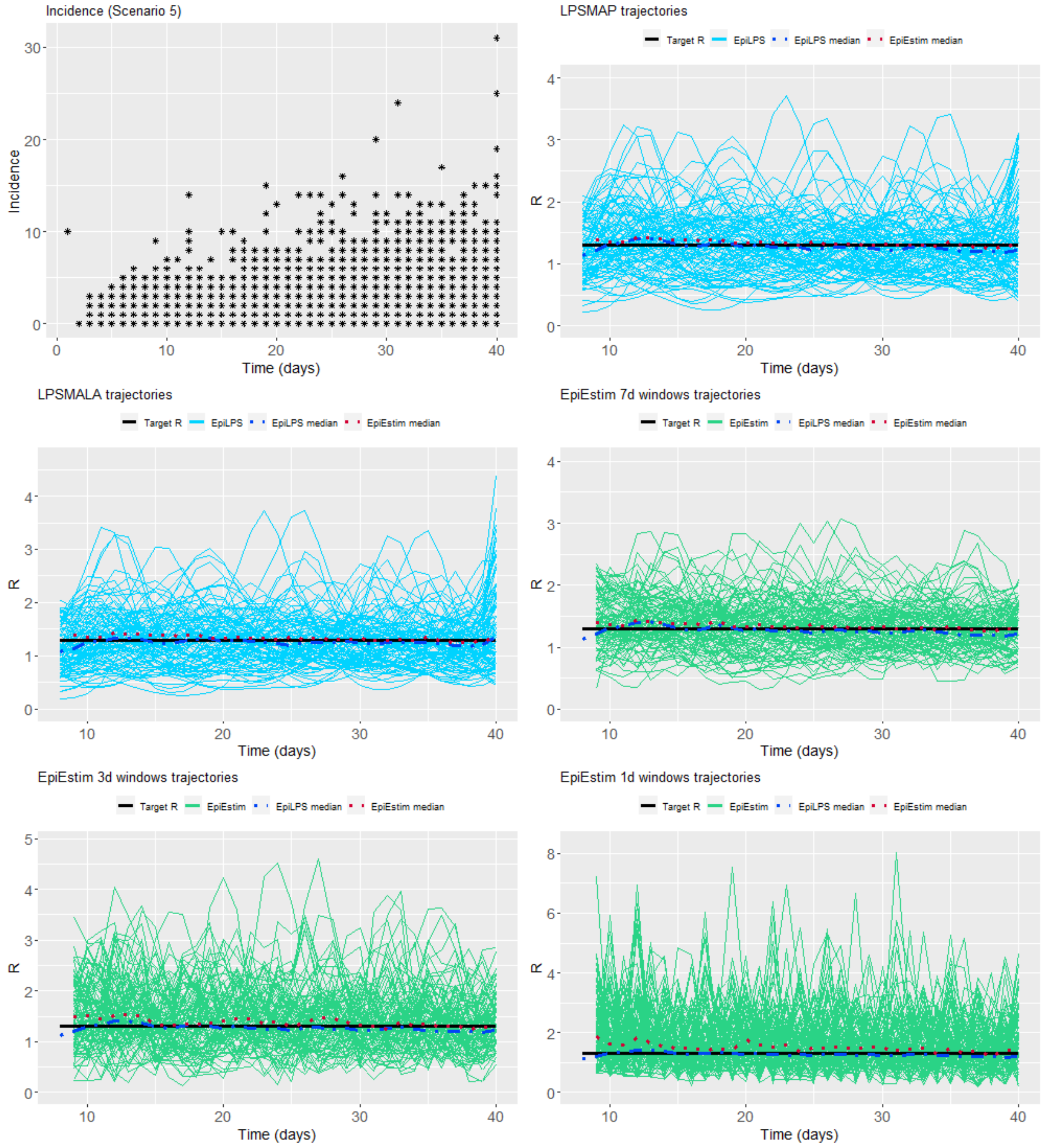

S2 Fig 5. Results for Scenario 5 considering S=100 simulated epidemics with a duration of T=40 days and a SARS-CoV-1 like serial interval. Top left panel represents the simulated epidemic curves. Estimated trajectories in light blue are for EpiLPS with LPSMAP and LPSMALA (with a chain length of 3 000 including a burn-in of 1 000) respectively, using K=40 B-splines and a second-order penalty. The green estimated trajectories are for EpiEstim under different sliding windows (weekly 7d, three days 3d and daily 1d) and estimated R(t) reported at the end of the window. Dashed (dotted) curves correspond to the pointwise median estimate of R(t) with EpiLPS (EpiEstim).

Scenario 6

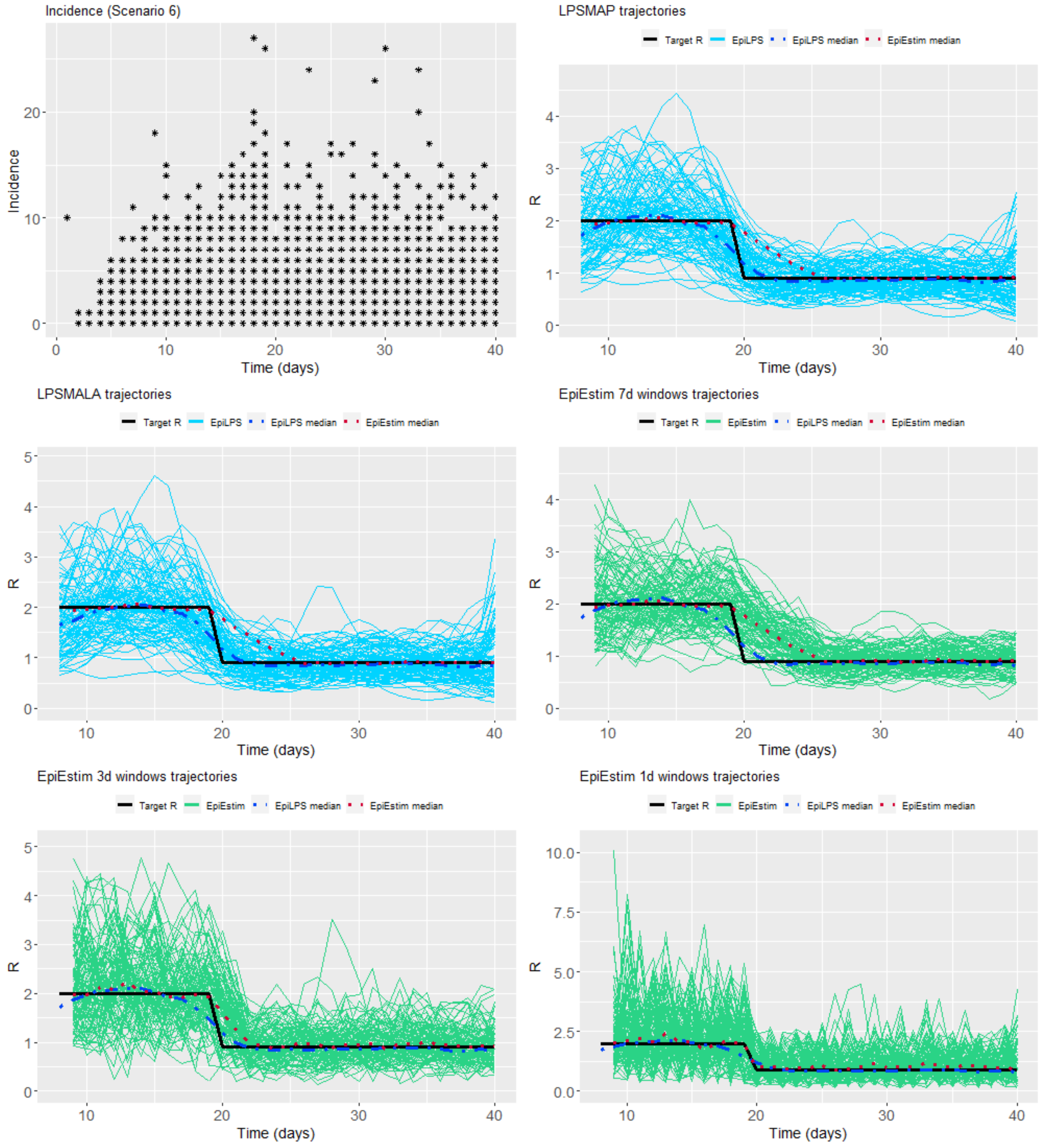

S2 Fig 6. ***Results for Scenario 6 considering S=100 simulated epidemics with a duration of T=40 days and a SARS-CoV-1 like serial interval. Top left panel represents the simulated epidemic curves. Estimated trajectories in light blue are for EpiLPS with LPSMAP and LPSMALA (with a chain length of 3 000 including a burn-in of 1 000) respectively, using K=40 B-splines and a second-order penalty. The green estimated trajectories are for EpiEstim under different sliding windows (weekly 7d, three days 3d and daily 1d) and estimated R(t) reported at the end of the window. Dashed (dotted) curves correspond to the pointwise median estimate of R(t) with EpiLPS (EpiEstim).***

Scenario 7

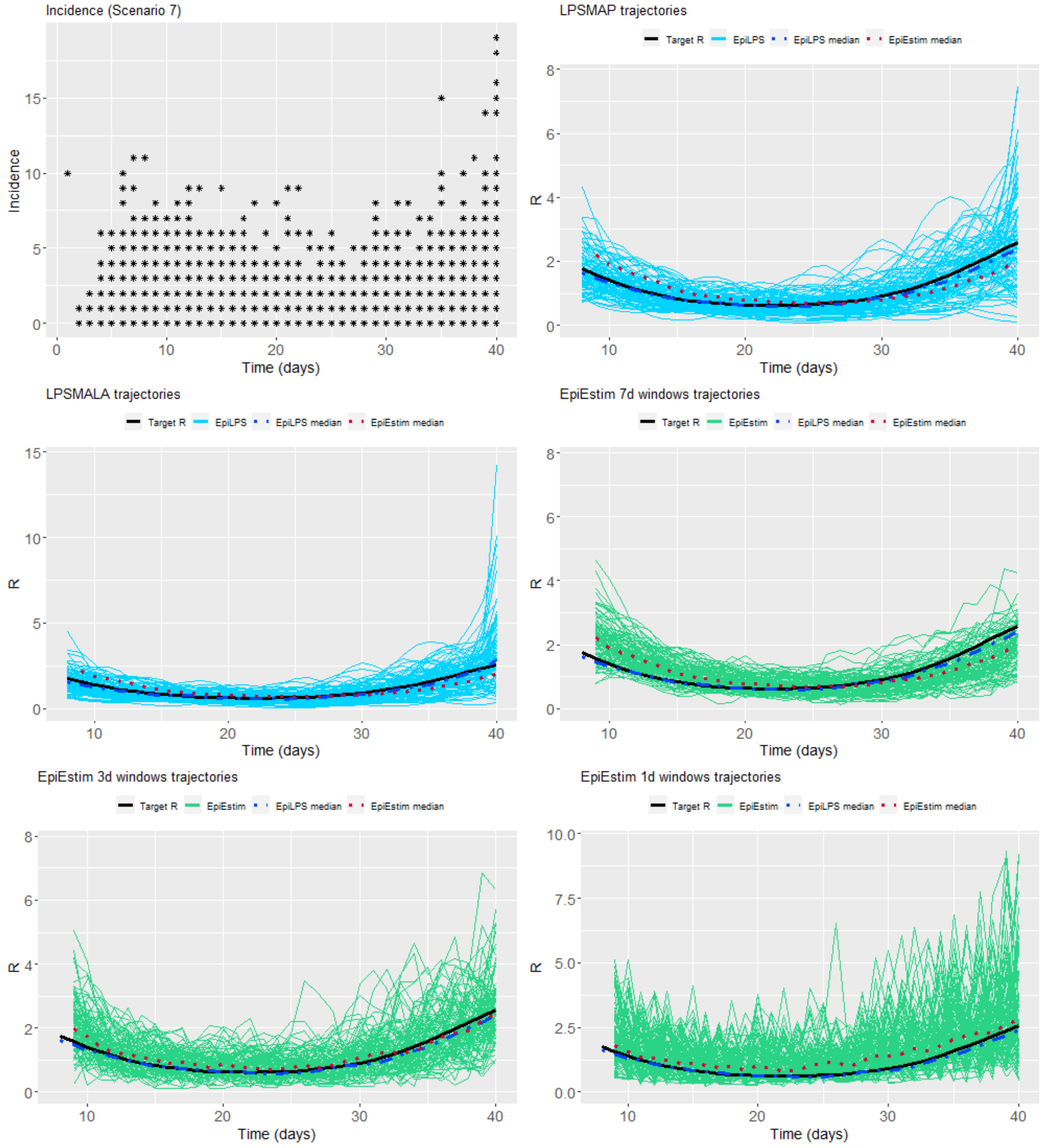

S2 Fig 7. Results for Scenario 7 considering S=100 simulated epidemics with a duration of T=40 days and a SARS-CoV-1 like serial interval. Top left panel represents the simulated epidemic curves. Estimated trajectories in light blue are for EpiLPS with LPSMAP and LPSMALA (with a chain length of 3 000 including a burn-in of 1 000) respectively, using K=40 B-splines and a second-order penalty. The green estimated trajectories are for EpiEstim under different sliding windows (weekly 7d, three days 3d and daily 1d) and estimated R(t) reported at the end of the window. Dashed (dotted) curves correspond to the pointwise median estimate of R(t) with EpiLPS (EpiEstim).

Scenario 8

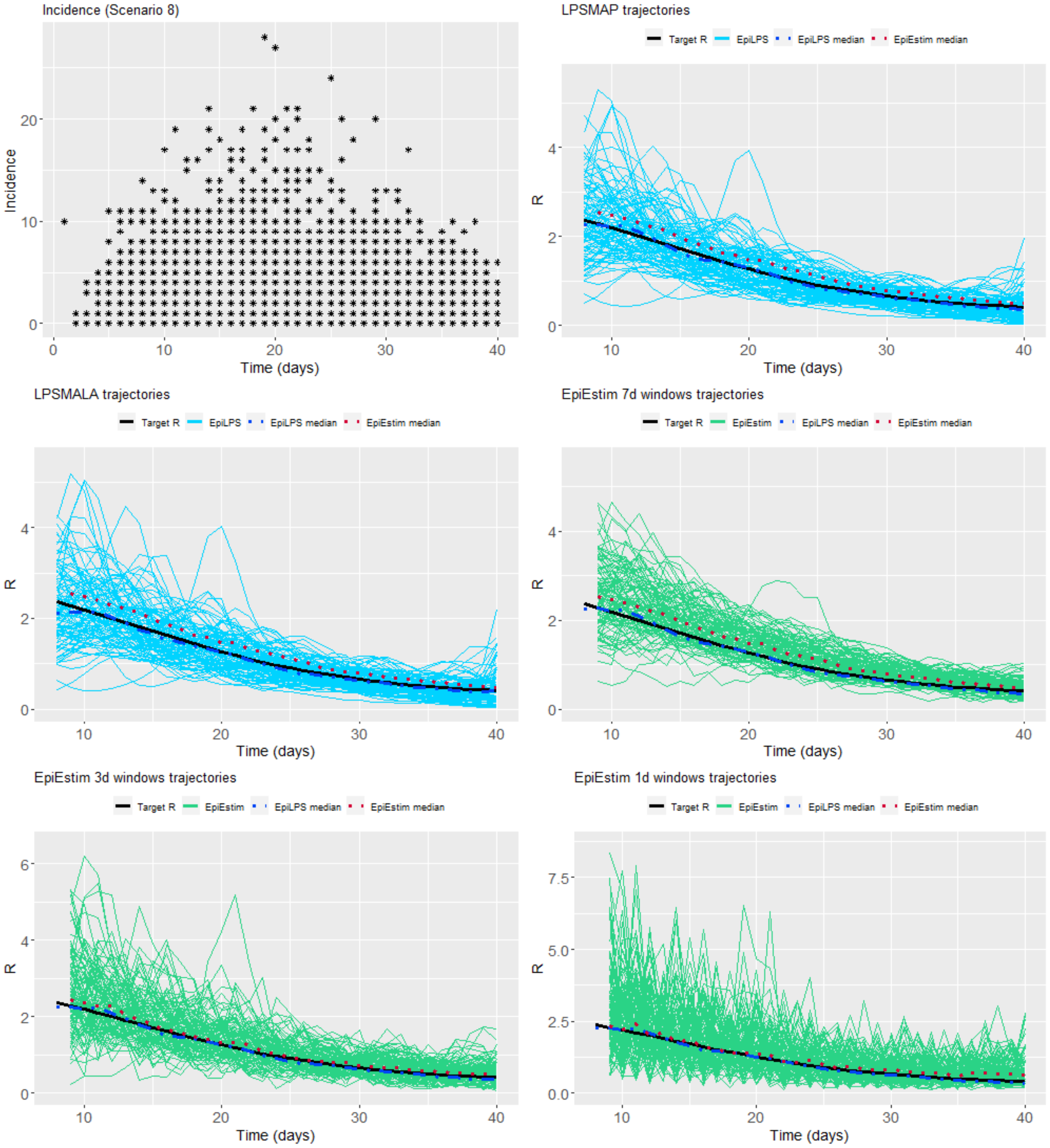

S2 Fig 8. ***Results for Scenario 8 considering S=100 simulated epidemics with a duration of T=40 days and a SARS-CoV-1 like serial interval. Top left panel represents the simulated epidemic curves. Estimated trajectories in light blue are for EpiLPS with LPSMAP and LPSMALA (with a chain length of 3 000 including a burn-in of 1 000) respectively, using K=40 B-splines and a second-order penalty. The green estimated trajectories are for EpiEstim under different sliding windows (weekly 7d, three days 3d and daily 1d) and estimated R(t) reported at the end of the window. Dashed (dotted) curves correspond to the pointwise median estimate of R(t) with EpiLPS (EpiEstim).***

Scenario 9

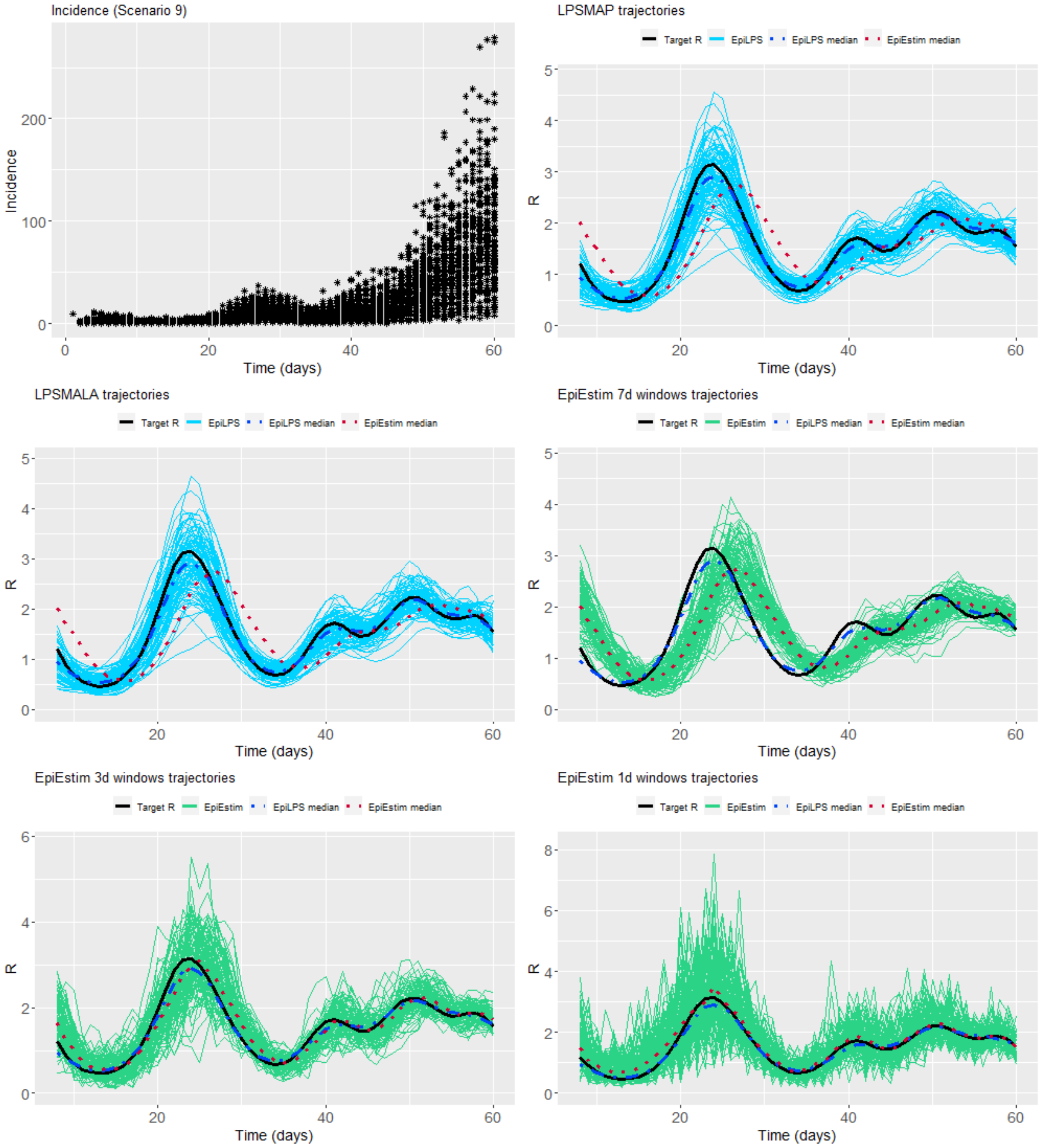

S2 Fig 9. ***Results for Scenario 9 considering S=100 simulated epidemics with a duration of T=60 days and a MERS-CoV like serial interval. Top left panel represents the simulated epidemic curves. Estimated trajectories in light blue are for EpiLPS with LPSMAP and LPSMALA (with a chain length of 3 000 including a burn-in of 1 000) respectively, using K=40 B-splines and a second-order penalty. The green estimated trajectories are for EpiEstim under different sliding windows (weekly 7d, three days 3d and daily 1d) and estimated R(t) reported at the end of the window. Dashed (dotted) curves correspond to the pointwise median estimate of R(t) with EpiLPS (EpiEstim).***

5. Plot of the mean and variance of y_t in Scenarios 1-9

Let $E(y_t)^{(s)}$ and $V(y_t)^{(s)}$ denote the mean and variance of y_t at iteration s of the simulation. Figures below show these quantities on a same plot for each scenario across all the simulated epidemics $s = 1, \dots, 100$, in order to have an idea of the degree of overdispersion present in the data. In Scenarios 1-4, the overdispersion is much stronger as compared to the other scenarios. Therefore, the simulations allow to assess the performance of EpiLPS and EpiEstim under varying levels of overdispersion across the considered scenarios.

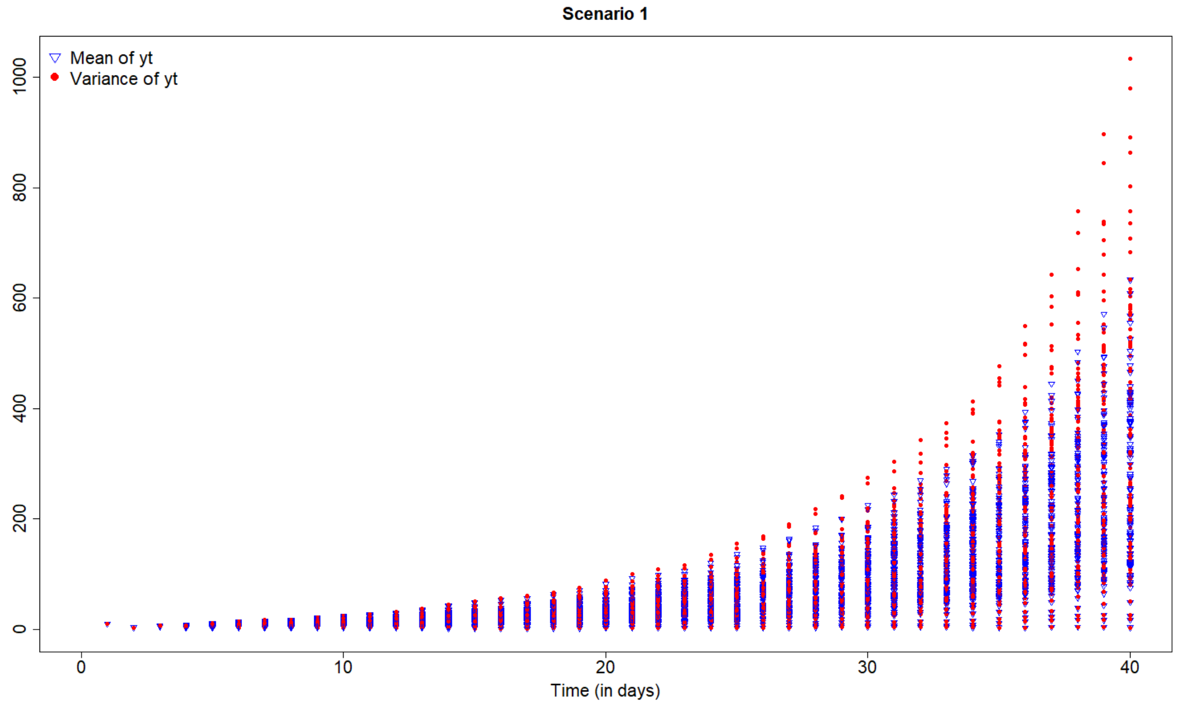

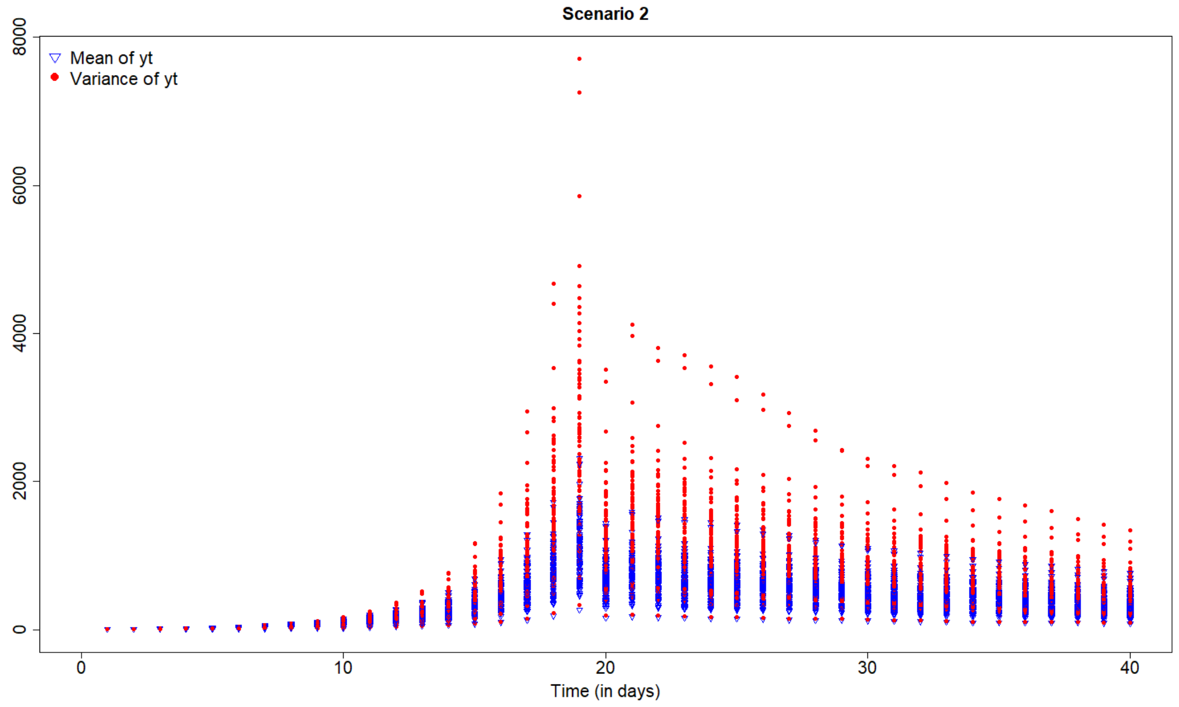

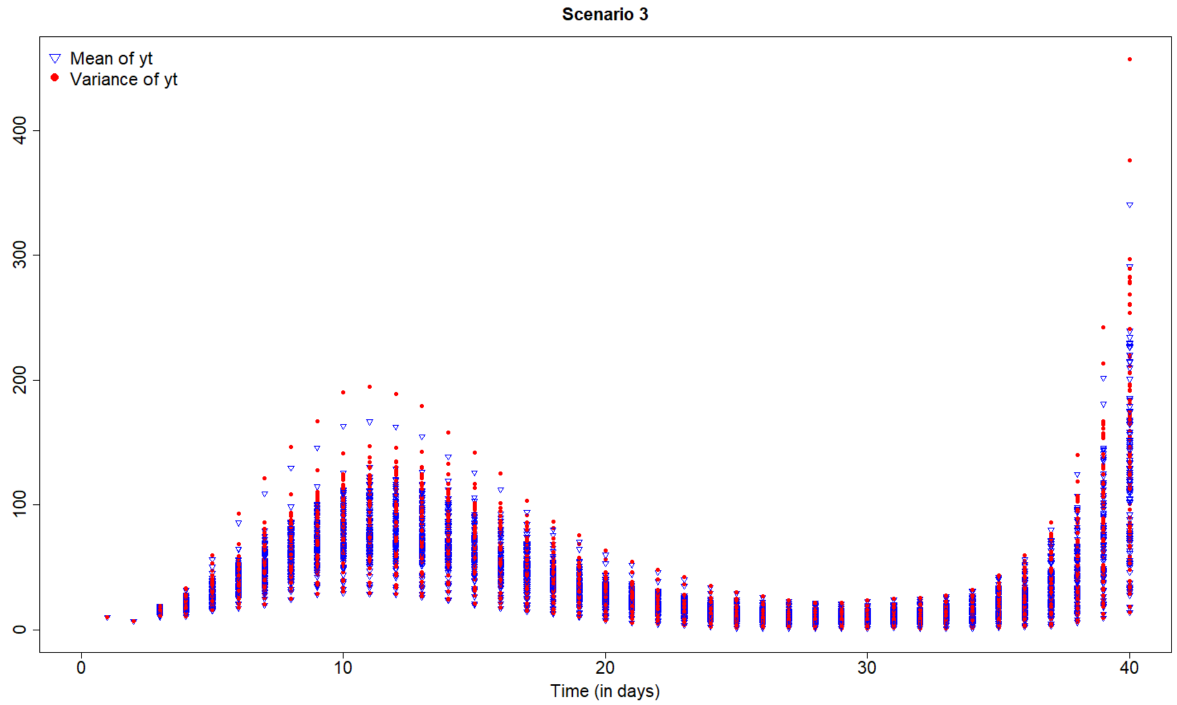
Scenario 3
Mean of yt
Variance of yt
0
100
200
300
400
0
10
20
30
40
Time (in days)

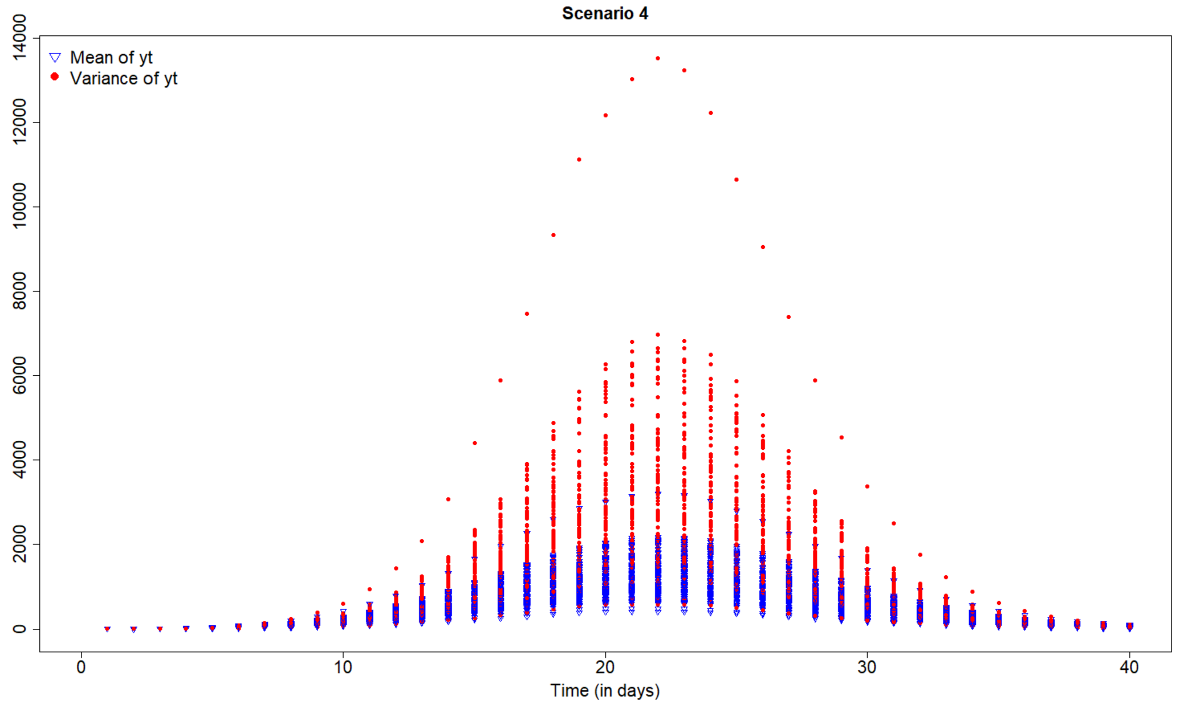
Scenario 4
Mean of yt
Variance of yt
0
2000
4000
6000
8000
10000
12000
14000
0
10
20
30
40
Time (in days)

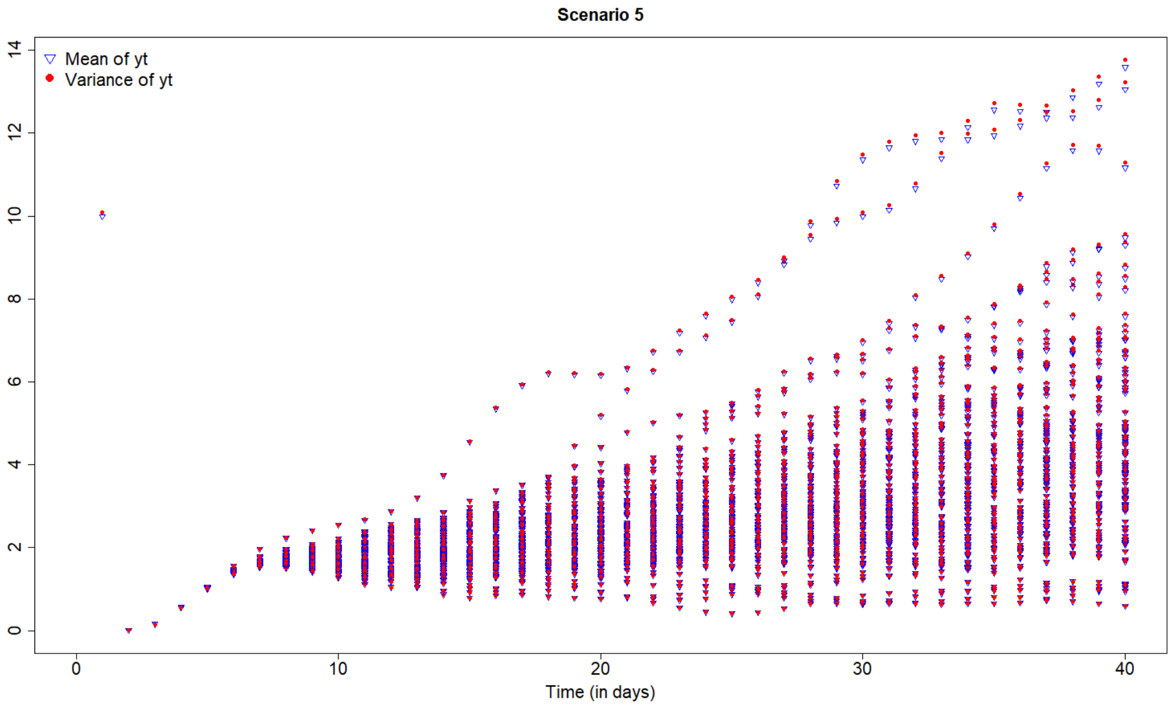
Scenario 5
Mean of yt
Variance of yt
Time (in days)
0
10
20
30
40
0
2
4
6
8
10
12
14

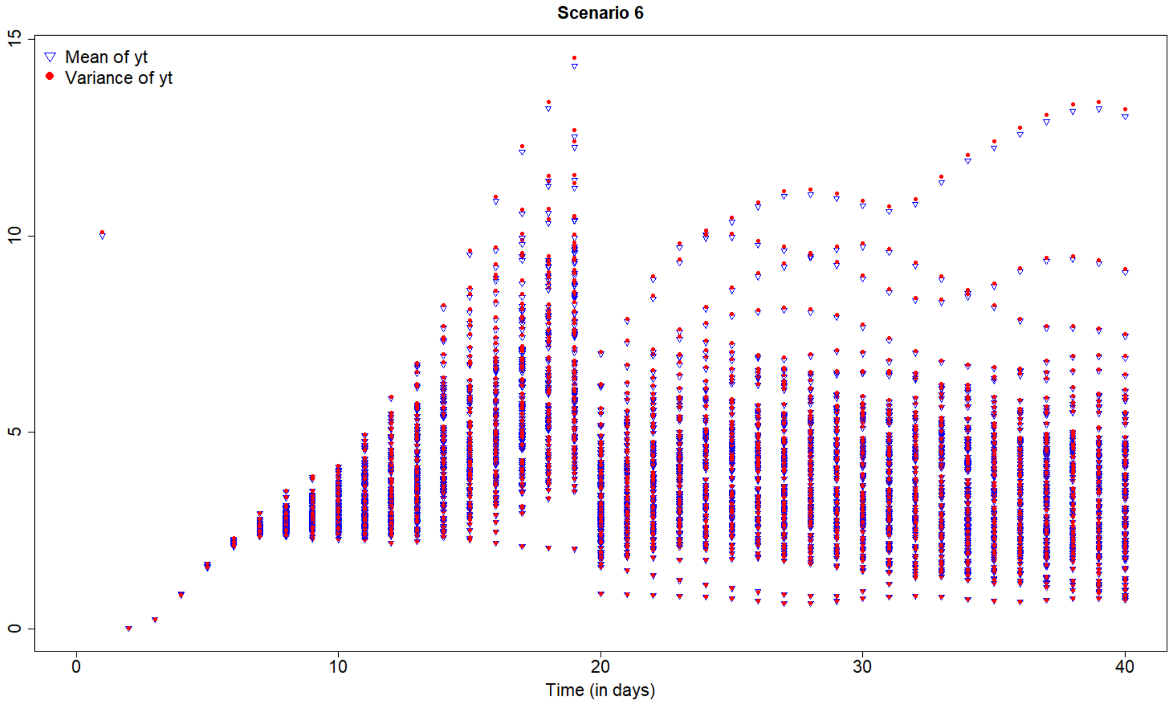
Scenario 6
Mean of yt
Variance of yt
Time (in days)
0
10
20
30
40
0
5
10
15

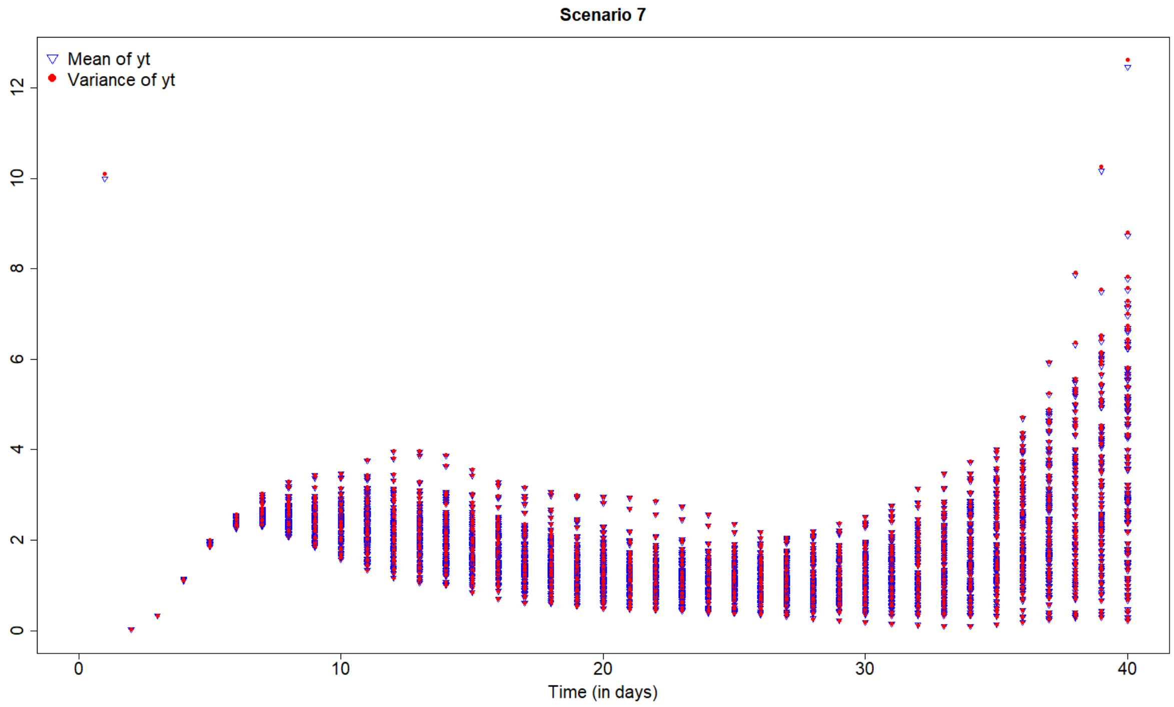
Scenario 7
Mean of yt
Variance of yt
0
2
4
6
8
10
12
0
10
20
30
40
Time (in days)

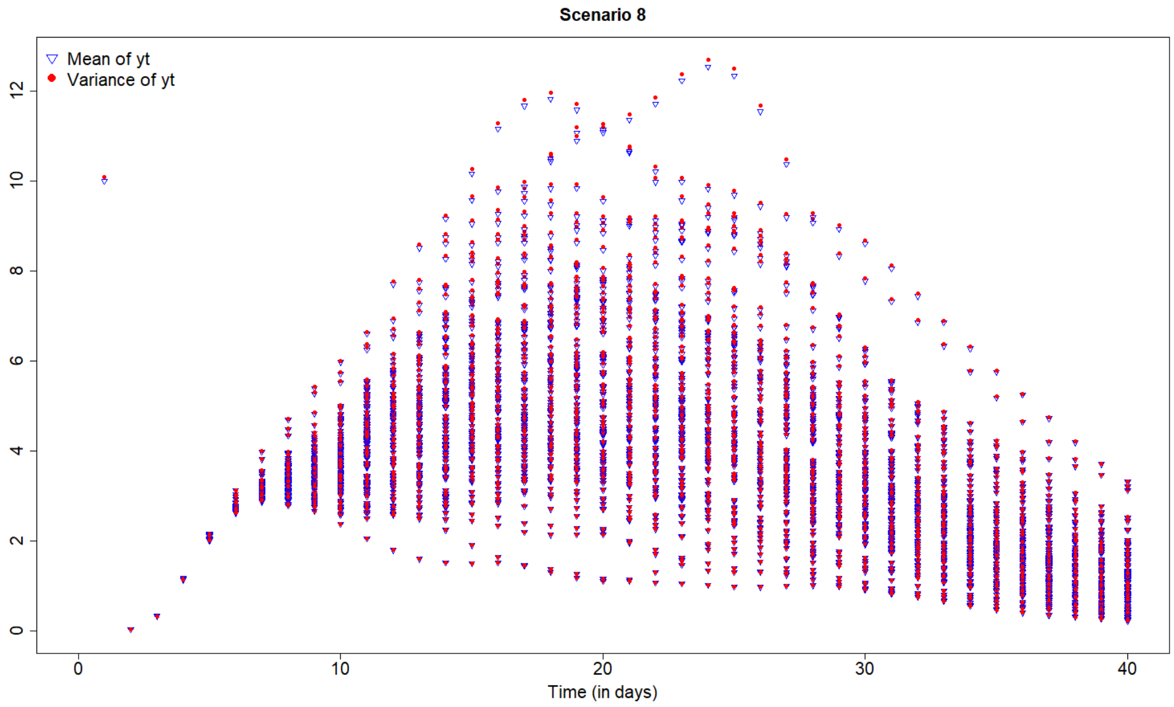
Scenario 8
Mean of yt
Variance of yt
0
2
4
6
8
10
12
0
10
20
30
40
Time (in days)

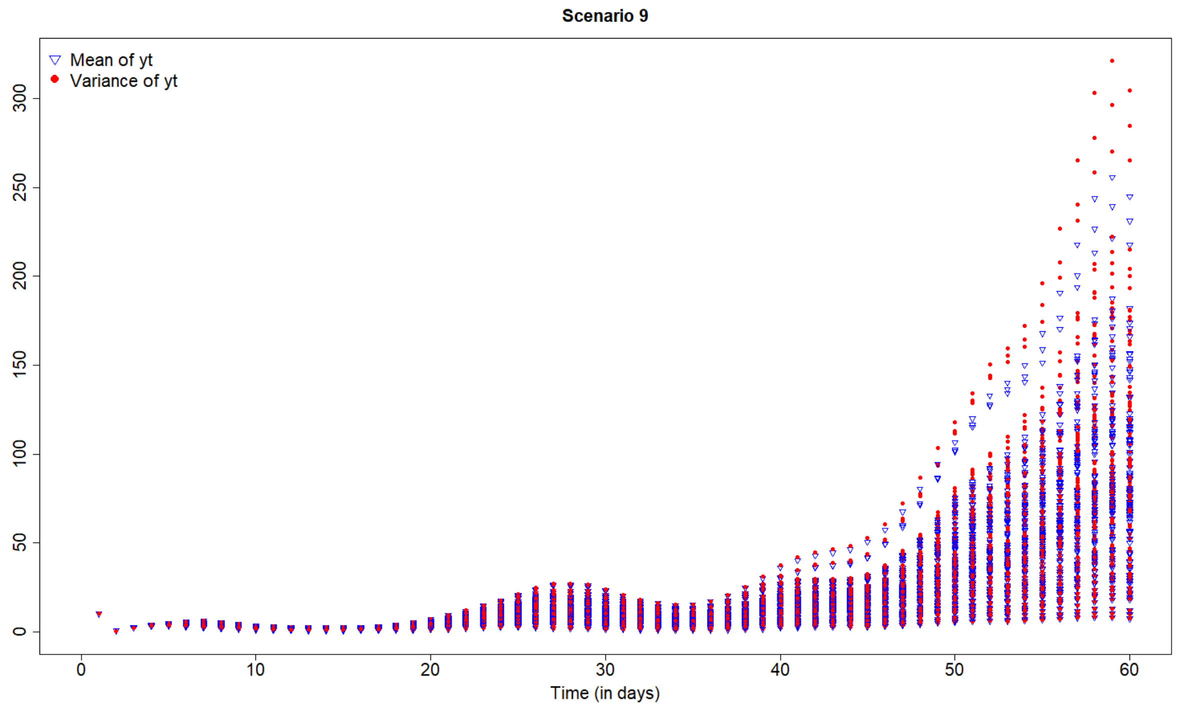

6. Sensitivity analyses

Sensitivity with respect to prior choice of δ

The dispersion hyperparameter δ is assumed to have a Gamma prior with default shape parameter $a_\delta = 10$ and default rate parameter $b_\delta = 10$ (so that the hyperprior for δ has mean $a_\delta/b_\delta = 1$ and variance $a_\delta/b_\delta^2 = 0.1$). The sensitivity of the fitted $R(t)$ curve (with LPSMAP, K = 40 B-splines and a second-order penalty) to different couples (a_δ, b_δ) is measured by simulating $S = 50$ epidemics of T days under Scenario 3 and 9 respectively. The median $R(t)$ estimate is then computed across the $S = 50$ epidemics at time point $t = 8, \dots, T$. Following Jullion and Lambert (2007) [5], we suggest to use EpiLPS with couples satisfying $a_\delta = b_\delta$, i.e., the mean of the δ hyperprior is equal to 1. The sensitivity analysis is therefore implemented with couples satisfying this constraint as shown in Table 3.

Couple (a_δ, b_δ)	Mean a_δ/b_δ	Variance a_δ/b_δ^2
$a_\delta = 5;\quad b_\delta = 5$	1	0.200
$a_\delta = 10;\ b_\delta = 10$	1	0.100
$a_\delta = 20;\ b_\delta = 20$	1	0.050
$a_\delta = 30;\ b_\delta = 30$	1	0.033
$a_\delta = 50;\ b_\delta = 50$	1	0.020
$a_\delta = 60;\ b_\delta = 60$	1	0.017

S2 Table 3. Couples of a_δ and b_δ chosen to measure the sensitivity of the fitted $R(t)$ curve.

Figures 10 and 11 show the estimated median $R(t)$ trajectories for Scenario 3 and 9 respectively under different specifications for a_δ and b_δ. For Scenario 3, the fitted curves cannot be distinguished. For Scenario 9, the fitted curves are almost the same and the $R(t)$ estimate with LPSMAP has little sensitivity with respect to the choice of a_δ and b_δ. For $a_\delta = b_\delta = 5$ in Scenario 9, the median $R(t)$ trajectory is slightly more biased in regions where the target function has more pronounced curvature.

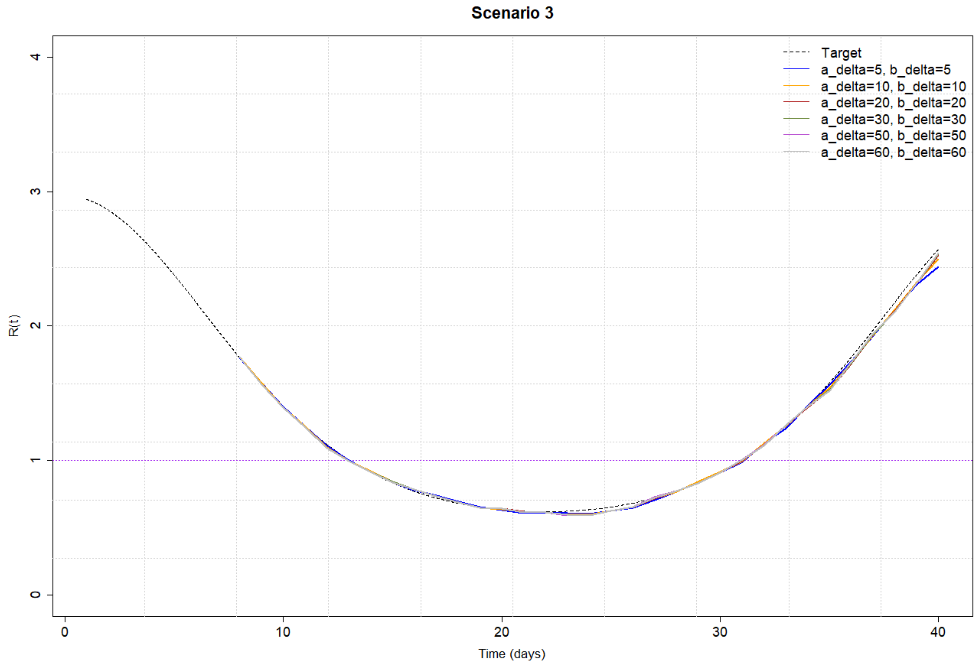

***S2 Fig 10.* Median R(t) trajectories (computed over S=50 epidemics) with different values for a_δ and b_δ under Scenario 3. R(t) estimates are obtained with LPSMAP using K=40 B-splines and a second-order penalty.**

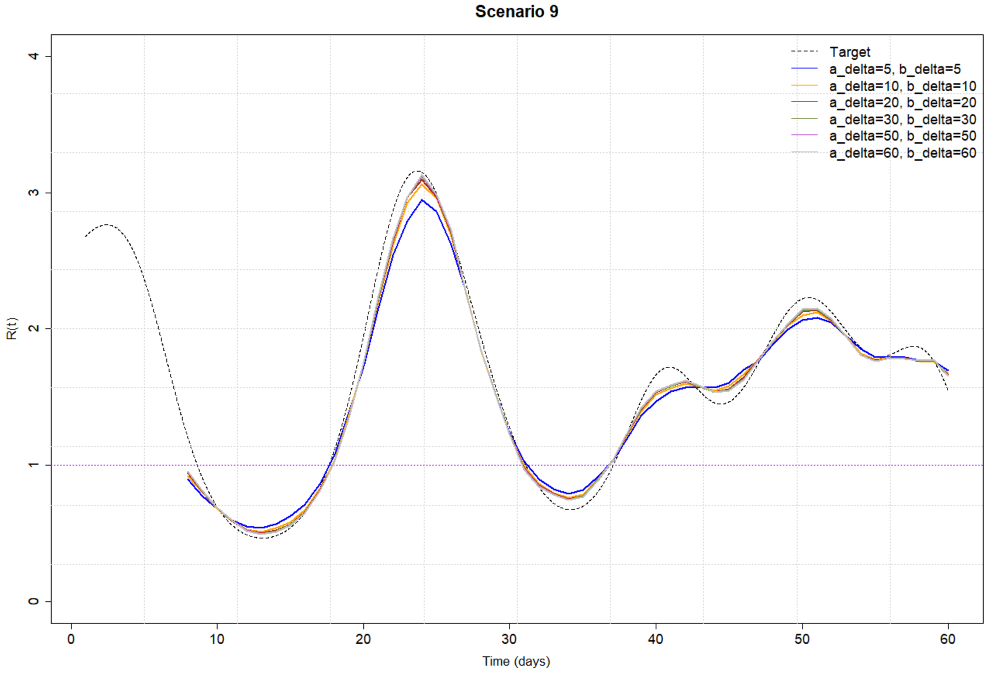

***S2 Fig 11.* Median R(t) trajectories (computed over S=50 epidemics) with different values for a_δ and b_δ under Scenario 9. R(t) estimates are obtained with LPSMAP using K=40 B-splines and a second-order penalty.**

Sensitivity with respect to the number of B-splines K

The simulation study in the paper uses K = 40 B-splines. The philosophy behind P-splines is to use a "large" number of B-spline basis functions. There is in principle no risk of specifying too many of them as the penalty will be in charge for counterbalancing the flexibility. However, the computational cost for implementing the EpiLPS algorithm will increase with K (see the next section on computing time). Figures 12 and 13 show the sensitivity of the median R(t) fit (computed with LPSMAP across 50 simulated epidemics) with respect to different basis sizes under Scenario 3 and 9 respectively. The figures show that the LPSMAP fits of the reproduction number are not sensitive with respect to the choice of K. In Figure 13, the use of K = 20 B-splines gives a satisfactory smooth fit to the target $R(t)$ but regions with high curvature are captured less precisely. Regions with more curvature can better be captured when more B-splines are used (e.g. K $=$ 30).

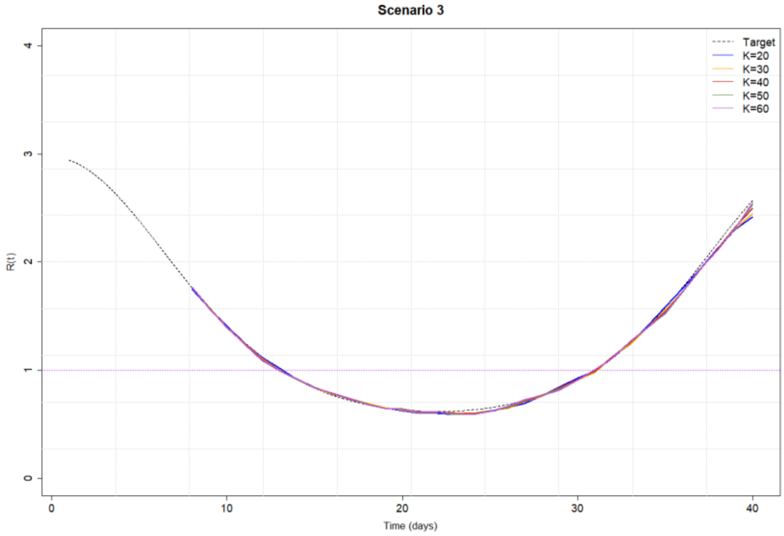

S2 Fig 12. Median R(t) trajectories for Scenario 3 (computed over S = 50 epidemics) with different sizes for the B-spline basis.

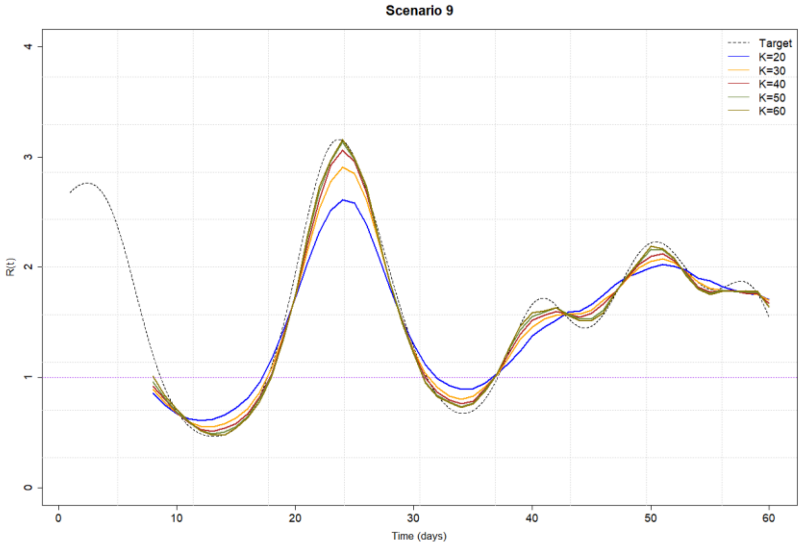

S2 Fig 13. Median R(t) trajectories for Scenario 9 (computed over S = 50 epidemics) with different sizes for the B-spline basis.

Sensitivity with respect to the B-splines domain $[1, T]$

EpiLPS is intrinsically a global smoothing approach in the sense that there is a single smoothing parameter λ; the key value that will determine the degree of smoothness of the fitted $R(t)$ curve. As such, applying EpiLPS on time domains with different widths such as $[1, T_1]$ and $[1, T_2]$ with $T_2 > T_1$ will yield different smoothing parameters, say, λ_1 (when applied to an epidemic of T_1 days) and λ_2 (when applied to an epidemic of T_2 days). This means that the resulting $R(t)$ fit at time $t \in [1, T_1]$ can potentially change when one applies EpiLPS to the wider domain $[1, T_2]$. This should not be considered a drawback of the approach. In fact, the main objective is to have a tool that accurately captures the evolution of the reproduction number over time. If past fitted $R(t)$ values slightly change when considering longer time domains but remain "close" to the target $R(t)$ value, then we can consider that the method is indeed effective in capturing the time trend of the reproduction number. We simulated data for an epidemic of $T = 60$ days under Scenario 9 and computed an estimate for $R(t)$ on rolling windows of days [1,10], [1,11],…,[1,60]. For each window, we computed the estimated $R(t)$ with LPSMAP and EpiEstim (with weekly sliding windows and $R(t)$ reported at the right boundary of the window and at the window midpoint, respectively) and plotted the estimates from day $t = 8$ to the upper bound of each time window. The light blue triangles in Figure 14 represent the rolling estimates with EpiLPS and the red diamonds are estimates obtained with EpiEstim (with $R(t)$ reported at the end of the window) . The orange, yellow and dark blue triangles highlight the $R(t)$ estimates with LPSMAP on domains [1,20], [1,40] and [1,60], respectively. We see that the EpiLPS estimates (despite varying at each time point t because of varying time windows) appear on average closer to the target reproduction number as compared to the estimates provided by EpiEstim (which do not change). Table 4 shows the mean absolute error (MAE) for $R(t)$ computed as $T^{-1} \sum_{t=1}^{T} |\hat{R}(t) - R(t)|$ for epidemics of duration $T \in \{20,25,30,35,40,45,50,55,60\}$ for the two methods.

Time domain	EpiLPS	EpiEstim (window boundary)	EpiEstim (window midpoint)
[1,20]	0.192	0.627	0.197
[1,25]	0.108	0.715	0.177
[1,30]	0.171	0.663	0.248
[1,35]	0.163	0.670	0.233
[1,40]	0.154	0.628	0.213
[1,45]	0.148	0.564	0.198
[1,50]	0.149	0.533	0.181
[1,55]	0.178	0.507	0.169
[1,60]	0.189	0.473	0.165

***S2 Table 4.* Mean absolute error of R(t) estimates with LPSMAP and EpiEstim (with weekly sliding windows) on different time intervals.**

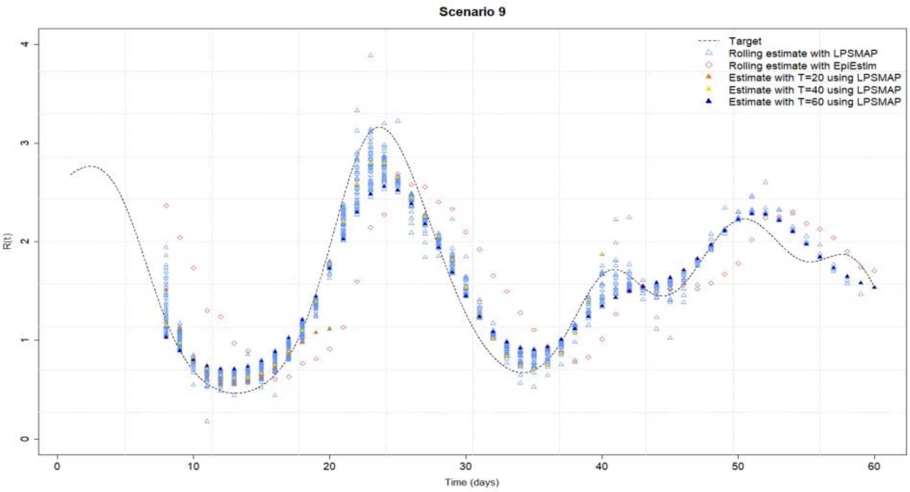

***S2 Fig 14.* Rolling R(t) estimates with LPSMAP (triangles) and EpiEstim (diamonds) with R(t) reported at the end of the window, computed on time windows ranging from [1,10] to [1,60].**

7. Computational time

The computational time of the EpiLPS algorithm is mainly affected by the number of B-splines considered in the basis (K) and the total number of days (T) of the epidemic. We therefore report the computational time required to run EpiLPS for different (T, K) combinations. Obviously, LPSMAP requires far less computational resources as it is a completely sampling-free approach relying on the maximum *a posteriori* estimate of the hyperparameter vector. Even with an epidemic of two months and $K = 60$, LPSMAP is extremely fast and delivers results in a fraction of a second. For LPSMALA, the computational times are larger as the algorithm relies on a MCMC scheme. However, even for $T = 60$ and $K = 60$, LPSMALA can be implemented in less than 10 seconds, which is a relatively reasonable time given the number of parameters involved in the model.

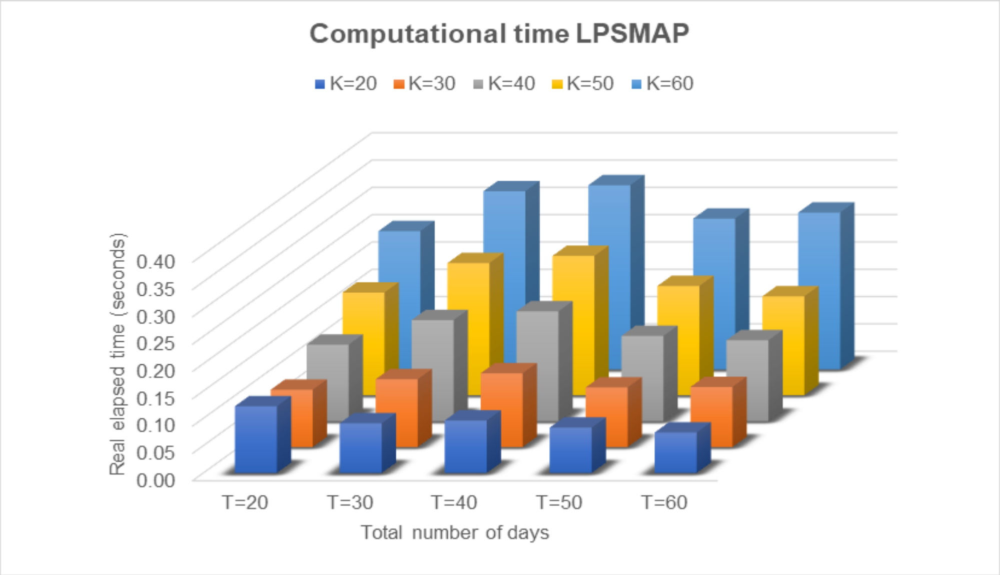

S2 Fig 15. *Computational time for LPSMAP under different (T, K)combinations.*

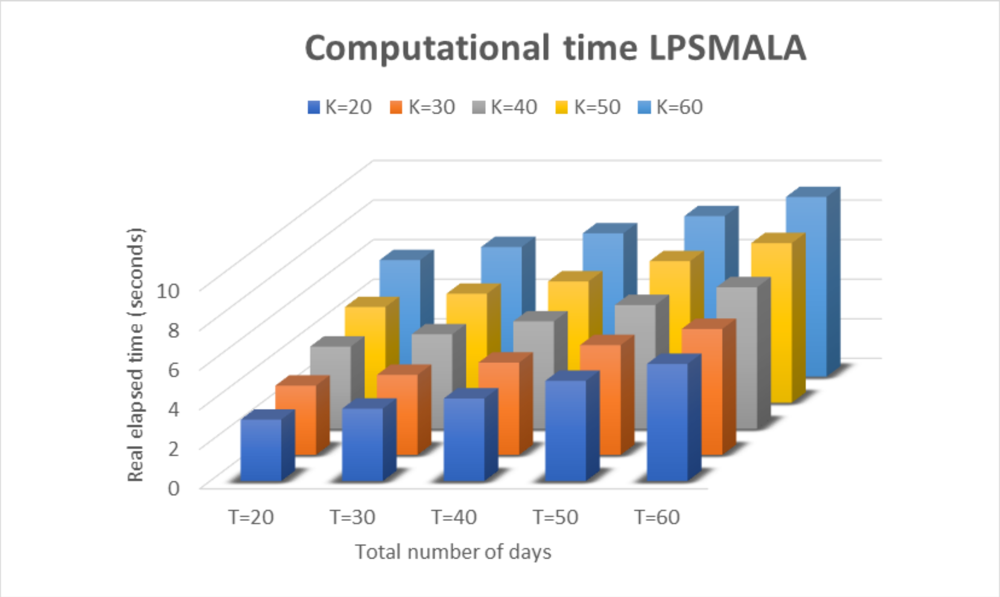

S2 Fig 16. *Computational time for LPSMALA with a chain length of 3 000 under different (T, K) combinations.*

References

1. Ferguson NM, Cummings DA, Cauchemez S, Fraser C, Riley S, Meeyai A, et al. Strategies for containing an emerging influenza pandemic in Southeast Asia. Nature. 2005;**437**(7056):209-214. https://doi.org/10.1038/nature04017

2. Cori A, Ferguson NM, Fraser C, Cauchemez S. A new framework and software to estimate time-varying reproduction numbers during epidemics. American Journal of Epidemiology. 2013;**178**(9):1505-1512. https://doi.org/10.1093/aje/kwt133

3. Lipsitch M, Cohen T, Cooper B, Robins JM, Ma S, James L, et al. Transmission dynamics and control of severe acute respiratory syndrome. Science. 2003;**300**(5627):1966-1970. https://doi.org/10.1126%2Fscience.1086616

4. Cauchemez S, Nouvellet P, Cori A, Jombart T, Garske T, Clapham H, et al. Unraveling the drivers of MERS-CoV transmission. Proceedings of the national academy of sciences. 2016;**113**(32):9081-9086. https://doi.org/10.1073/pnas.1519235113

5. Jullion A, Lambert P. Robust specification of the roughness penalty prior distribution in spatially adaptive Bayesian P-splines models. Computational Statistics & Data Analysis. 2007;**51**(5):2542-2558. https://doi.org/10.1016/j.csda.2006.09.027
