# Supplementary material for: EpiLPS: A fast and flexible Bayesian tool for estimation of the time-varying reproduction number
Source: PLoS Comput Biol. 2022 Oct 10;18(10):e1010618. doi: 10.1371/journal.pcbi.1010618 (PMC9584461; doi:10.1371/journal.pcbi.1010618)
Supplement: S3 Appendix — Complete simulation results (for EpiLPS and EpiEstim) when EpiEstim reports Rt at the window midpoint and additional sensitivity analyses. (PDF) [file pcbi.1010618.s003.pdf]

EpiLPS: A Fast and Flexible Bayesian Tool for Estimation of the Time-Varying Reproduction Number (Supplementary Information 3)

Oswaldo Gressani, Jacco Wallinga, Christian L. Althaus, Niel Hens, Christel Faes

Table of Contents

1. Sliding windows under the Cori et al. (2013) convention and the Gostic et al. (2020) recommendation 2

2. Figures of the simulation study with centered windows for EpiEstim 3

Scenario 1 3

Scenario 2 4

Scenario 3 5

Scenario 4 6

Scenario 5 7

Scenario 6 8

Scenario 7 9

Scenario 8 10

Scenario 9 11

3. Estimation performance for the overdispersion parameter 12

4. Are credible intervals affected by different choices of $a_\delta = b_\delta$? 12

1. Sliding windows under the Cori et al. (2013) convention and the Gostic et al. (2020) recommendation.

	Cori et al. (2013) convention $[t-\omega; t]$			Gostic et al. (2020) recommendation $[t-\omega/2; t+\omega/2]$		
t	ω=6 (weekly)	ω=2 (3 days)	ω=0 (daily)	ω=6 (weekly)	ω=2 (3 days)	ω=0 (daily)
8	[2 ; 8]	[6 ; 8]	8	[5 ; 11]	[7 ; 9]	8
9	[3 ; 9]	[7 ; 9]	9	[6 ; 12]	[8 ; 10]	9
10	[4 ; 10]	[8 ; 10]	10	[7 ; 13]	[9 ; 11]	10
⋮	⋮	⋮	⋮	⋮		⋮
37	[31 ; 37]	[35 ; 37]	37	[34 ; 40]	[36 ; 38]	37
38	[32 ; 38]	[36 ; 38]	38	Ø	[37 ; 39]	38
39	[33 ; 39]	[37 ; 39]	39	Ø	[38 ; 40]	39
40	[34 ; 40]	[38 ; 40]	40	Ø	Ø	40
57	[51 ; 57]	[55 ; 57]	57	[54 ; 60]	[56 ; 58]	57
58	[52 ; 58]	[56 ; 58]	58	Ø	[57 ; 59]	58
59	[53 ; 59]	[57 ; 59]	59	Ø	[58 ; 60]	59
60	[54 ; 60]	[58 ; 60]	60	Ø	Ø	60

S3 Table 1. ***Sliding windows of width ω for the nine epidemic scenarios over days t=8,…,40 (Scenarios 1-8) and days t=8,…,60 (Scenario 9). The table on the left is for the Cori et al. (2013) [1] convention, where the estimated R_t is reported at the end of the corresponding window. The table on the right is the Gostic et al. (2020) [2] recommendation that consists in reporting the estimated R_t at the midpoint of the smoothing window.***

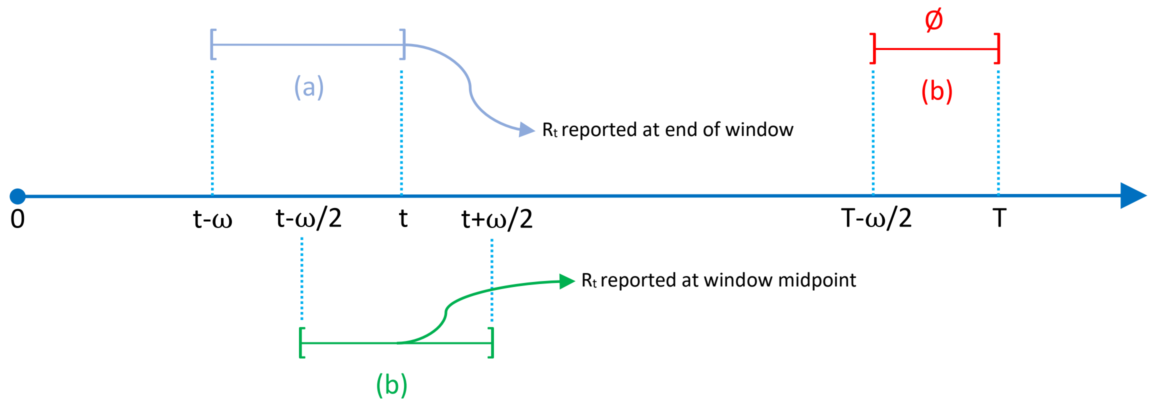

S3 Fig 1. ***Illustration of smoothing windows of width ω to estimate R_t with EpiEstim. (a) Cori et al. (2013) [1] convention with sliding windows $[t-\omega; t]$, where R_t is reported at the end of the window. (b) Gostic et al. (2020) [2] recommendation with centered sliding windows $[t-\omega/2; t+\omega/2]$, where R_t is reported at the midpoint of the window. Under the midpoint rule, R_t estimates for the last $\omega/2$ time units are unavailable.***

2. Figures of the simulation study with centered windows for EpiEstim

Scenario 1

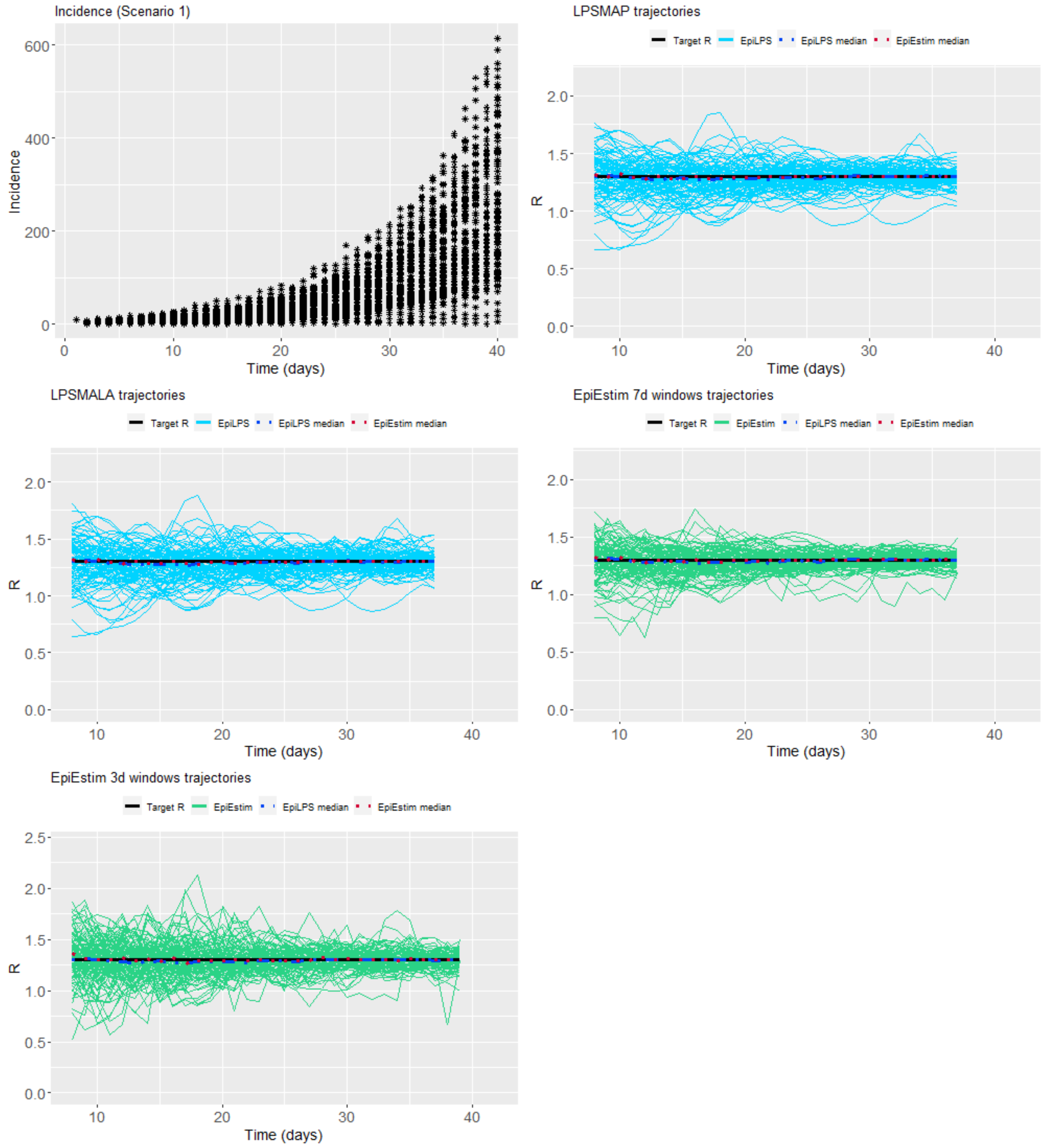

S3 Fig 2. Results for Scenario 1 considering S=100 simulated epidemics with a duration of T=40 days and a FLU like serial interval. Top left panel represents the simulated epidemic curves. Estimated trajectories in light blue are for EpiLPS with LPSMAP and LPSMALA (with a chain length of 3 000 including a burn-in of 1 000) respectively, using K=40 B-splines and a second-order penalty. The green estimated trajectories are for EpiEstim with R(t) reported at the midpoint of the smoothing window for weekly (7d) and three days (3d) windows. Dashed (dotted) curves correspond to the pointwise median estimate of R(t) with EpiLPS (EpiEstim).

Scenario 2

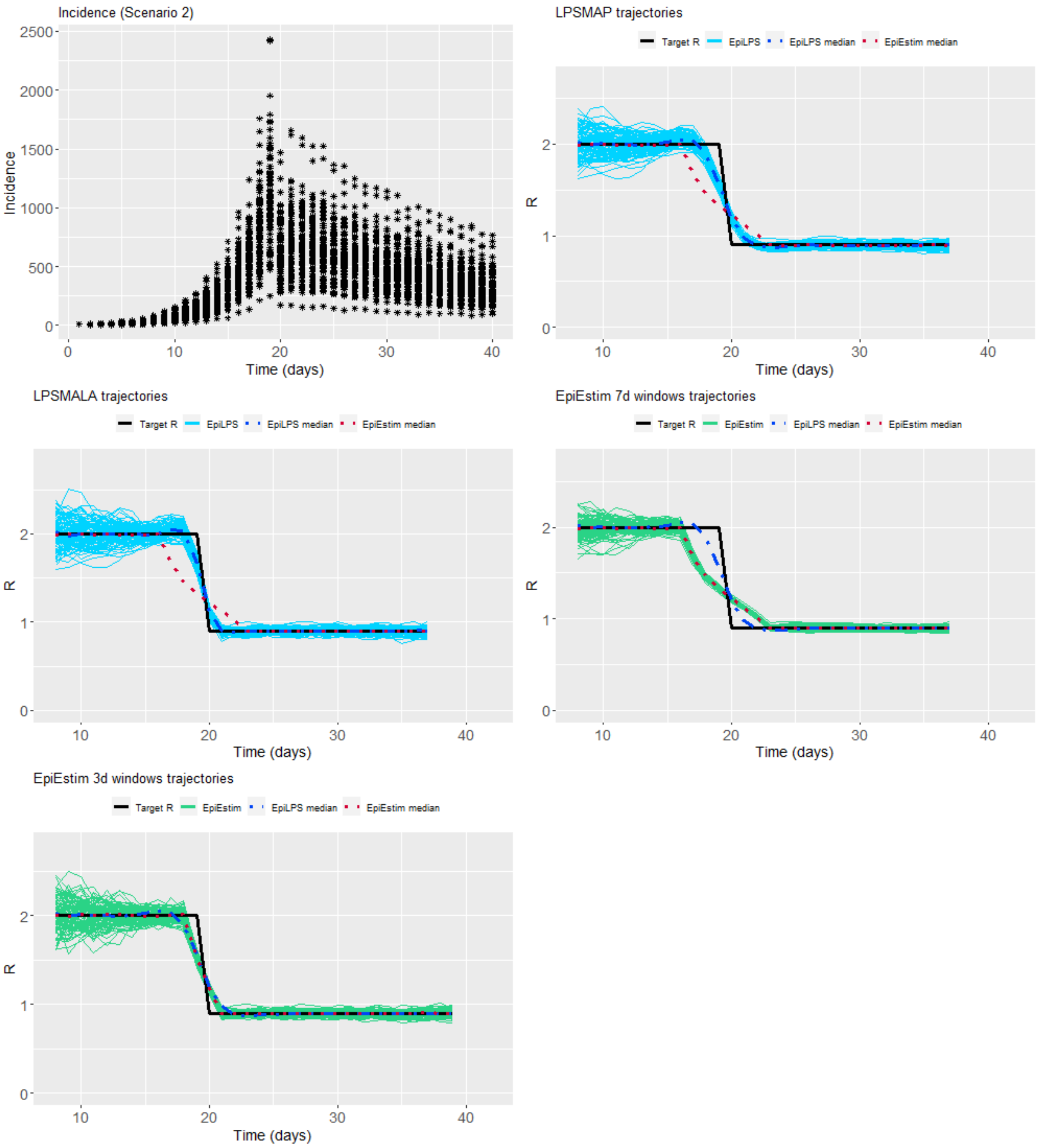

***S3 Fig 3.** Results for Scenario 2 considering S=100 simulated epidemics with a duration of T=40 days and a FLU like serial interval. Top left panel represents the simulated epidemic curves. Estimated trajectories in light blue are for EpiLPS with LPSMAP and LPSMALA (with a chain length of 3 000 including a burn-in of 1 000) respectively, using K=40 B-splines and a second-order penalty. The green estimated trajectories are for EpiEstim with R(t) reported at the midpoint of the smoothing window for weekly (7d) and three days (3d) windows. Dashed (dotted) curves correspond to the pointwise median estimate of R(t) with EpiLPS (EpiEstim).*

Scenario 3

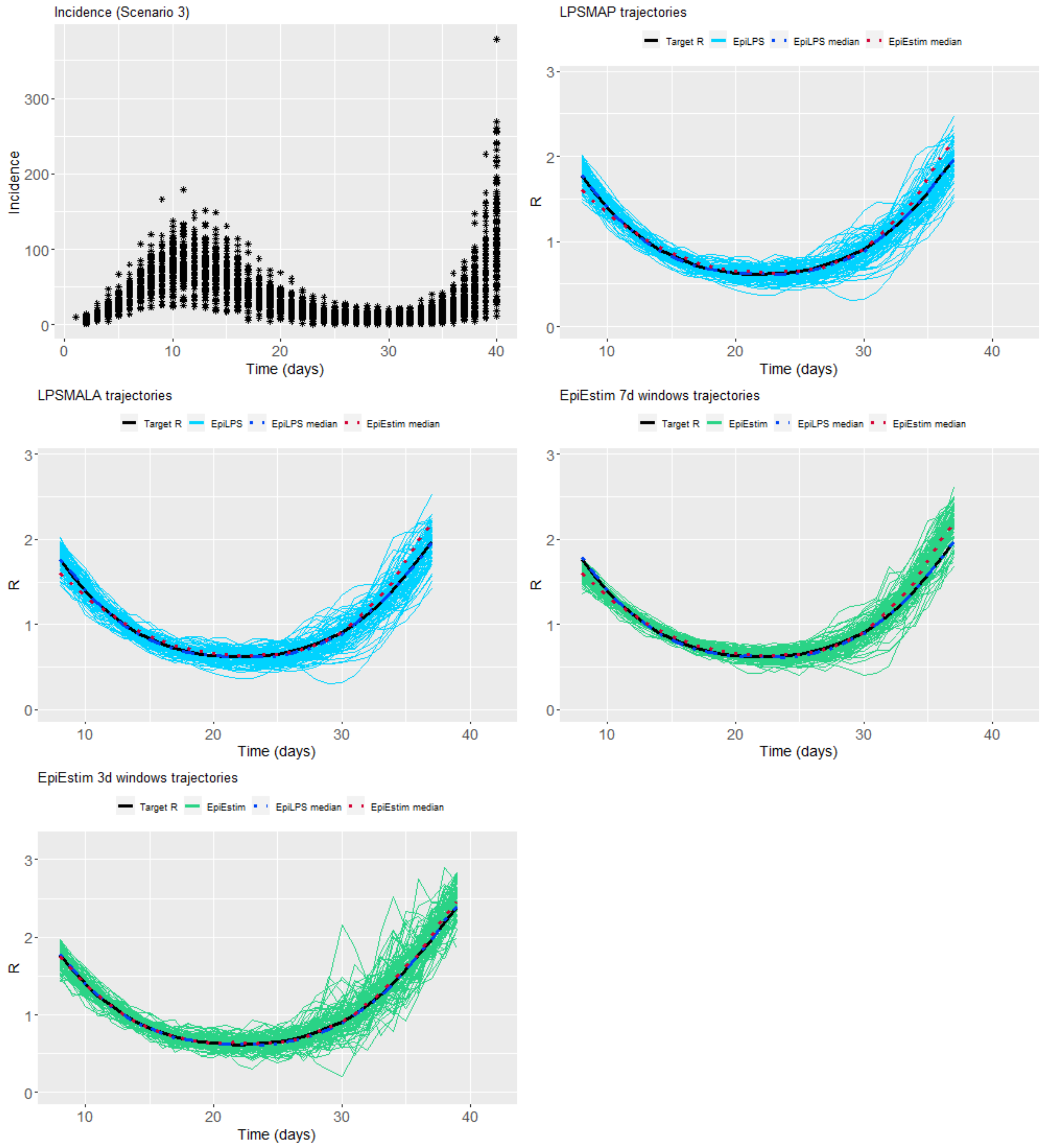

***S3 Fig 4.** Results for Scenario 3 considering S=100 simulated epidemics with a duration of T=40 days and a FLU like serial interval. Top left panel represents the simulated epidemic curves. Estimated trajectories in light blue are for EpiLPS with LPSMAP and LPSMALA (with a chain length of 3 000 including a burn-in of 1 000) respectively, using K=40 B-splines and a second-order penalty. The green estimated trajectories are for EpiEstim with R(t) reported at the midpoint of the smoothing window for weekly (7d) and three days (3d) windows. Dashed (dotted) curves correspond to the pointwise median estimate of R(t) with EpiLPS (EpiEstim).*

Scenario 4

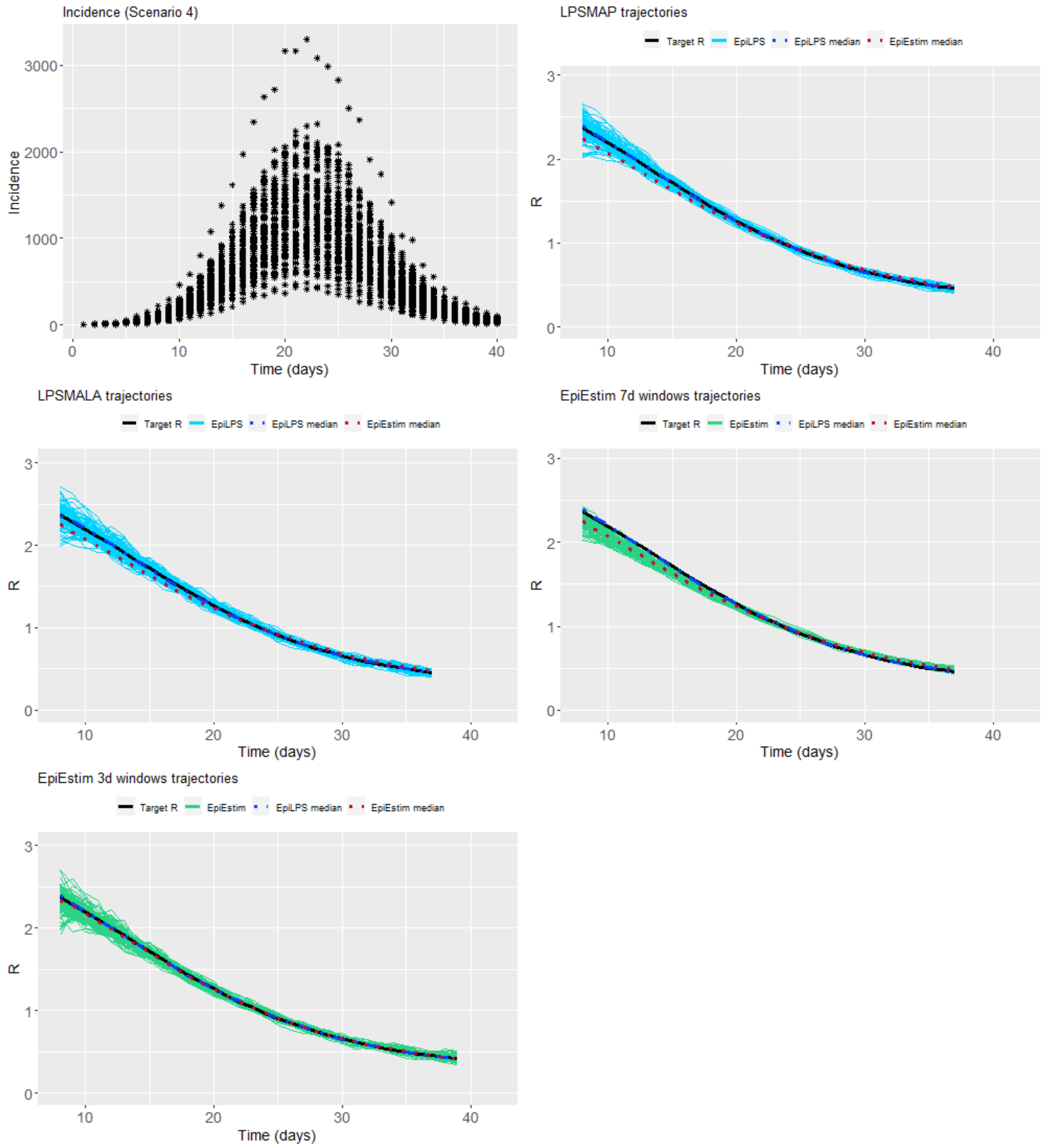

S3 Fig 5. ***Results for Scenario 4 considering S=100 simulated epidemics with a duration of T=40 days and a FLU like serial interval. Top left panel represents the simulated epidemic curves. Estimated trajectories in light blue are for EpiLPS with LPSMAP and LPSMALA (with a chain length of 3 000 including a burn-in of 1 000) respectively, using K=40 B-splines and a second-order penalty. The green estimated trajectories are for EpiEstim with R(t) reported at the midpoint of the smoothing window for weekly (7d) and three days (3d) windows. Dashed (dotted) curves correspond to the pointwise median estimate of R(t) with EpiLPS (EpiEstim).***

Scenario 5

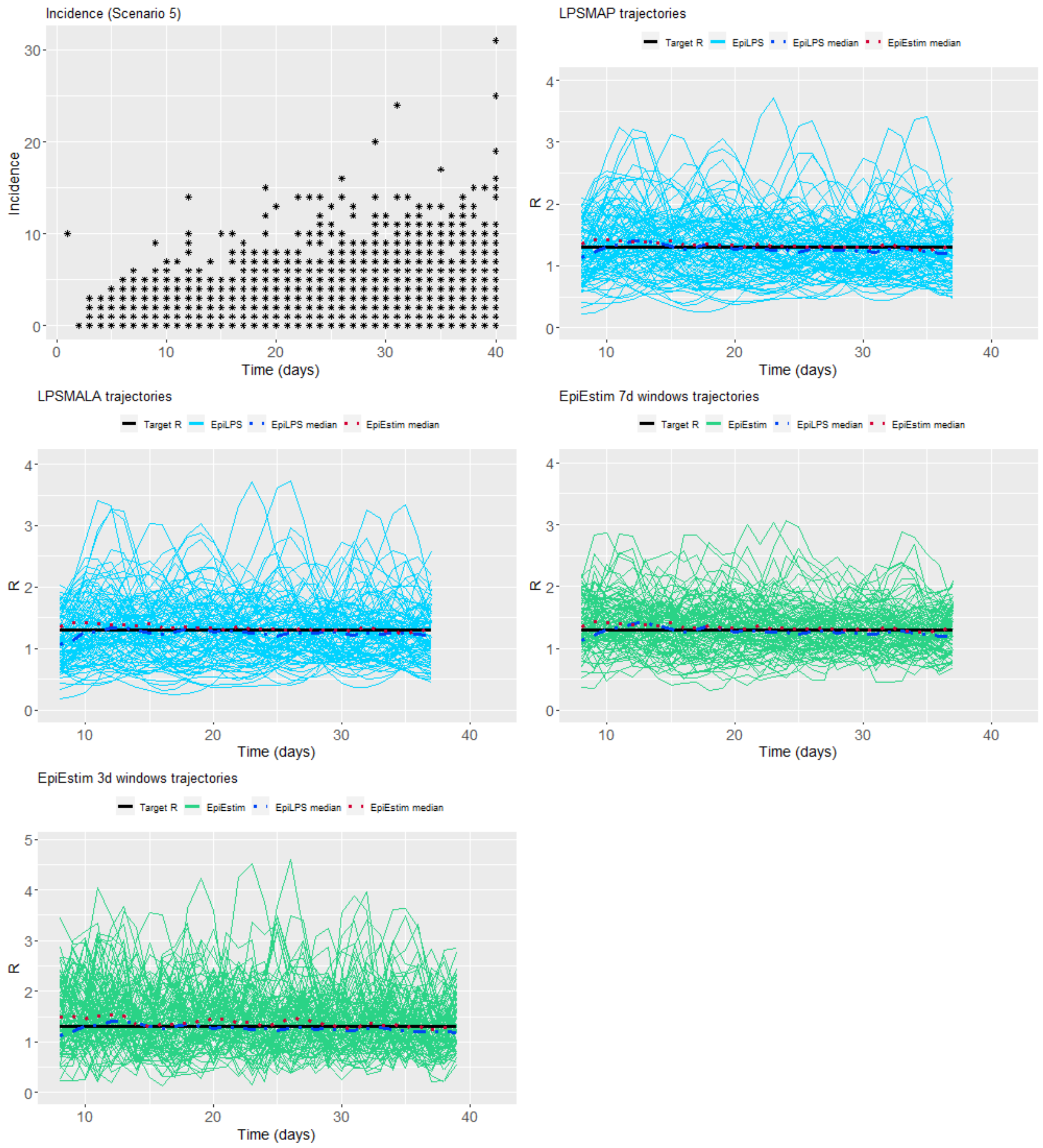

S3 Fig 6. Results for Scenario 5 considering S=100 simulated epidemics with a duration of T=40 days and a SARS-CoV-1 like serial interval. Top left panel represents the simulated epidemic curves. Estimated trajectories in light blue are for EpiLPS with LPSMAP and LPSMALA (with a chain length of 3 000 including a burn-in of 1 000) respectively, using K=40 B-splines and a second-order penalty. The green estimated trajectories are for EpiEstim with R(t) reported at the midpoint of the smoothing window for weekly (7d) and three days (3d) windows. Dashed (dotted) curves correspond to the pointwise median estimate of R(t) with EpiLPS (EpiEstim).

Scenario 6

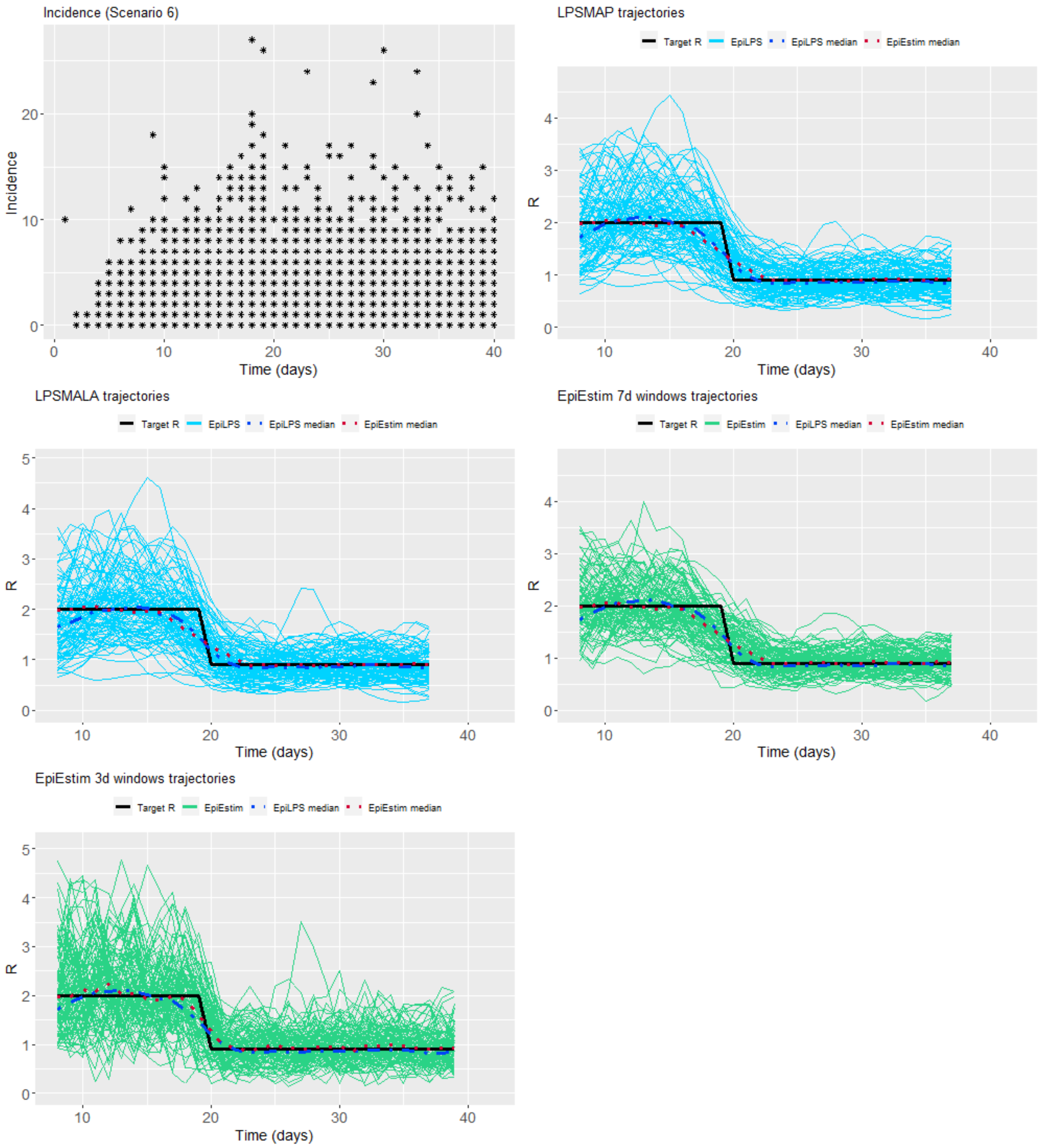

S3 Fig 7. ***Results for Scenario 6 considering S=100 simulated epidemics with a duration of T=40 days and a SARS-CoV-1 like serial interval. Top left panel represents the simulated epidemic curves. Estimated trajectories in light blue are for EpiLPS with LPSMAP and LPSMALA (with a chain length of 3 000 including a burn-in of 1 000) respectively, using K=40 B-splines and a second-order penalty. The green estimated trajectories are for EpiEstim with R(t) reported at the midpoint of the smoothing window for weekly (7d) and three days (3d) windows. Dashed (dotted) curves correspond to the pointwise median estimate of R(t) with EpiLPS (EpiEstim).***

Scenario 7

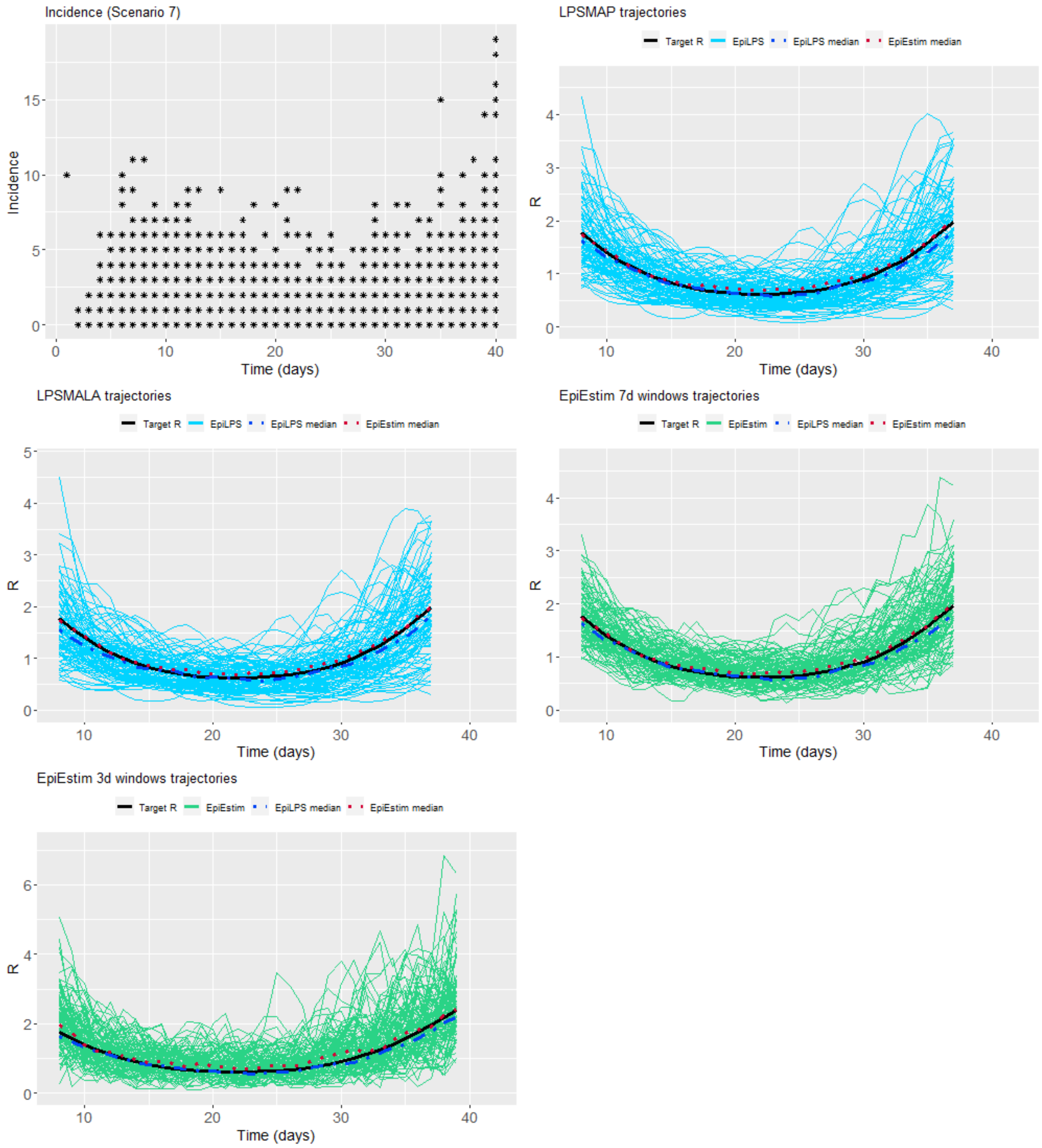

S3 Fig 8. ***Results for Scenario 7 considering S=100 simulated epidemics with a duration of T=40 days and a SARS-CoV-1 like serial interval. Top left panel represents the simulated epidemic curves. Estimated trajectories in light blue are for EpiLPS with LPSMAP and LPSMALA (with a chain length of 3 000 including a burn-in of 1 000) respectively, using K=40 B-splines and a second-order penalty. The green estimated trajectories are for EpiEstim with R(t) reported at the midpoint of the smoothing window for weekly (7d) and three days (3d) windows. Dashed (dotted) curves correspond to the pointwise median estimate of R(t) with EpiLPS (EpiEstim).***

Scenario 8

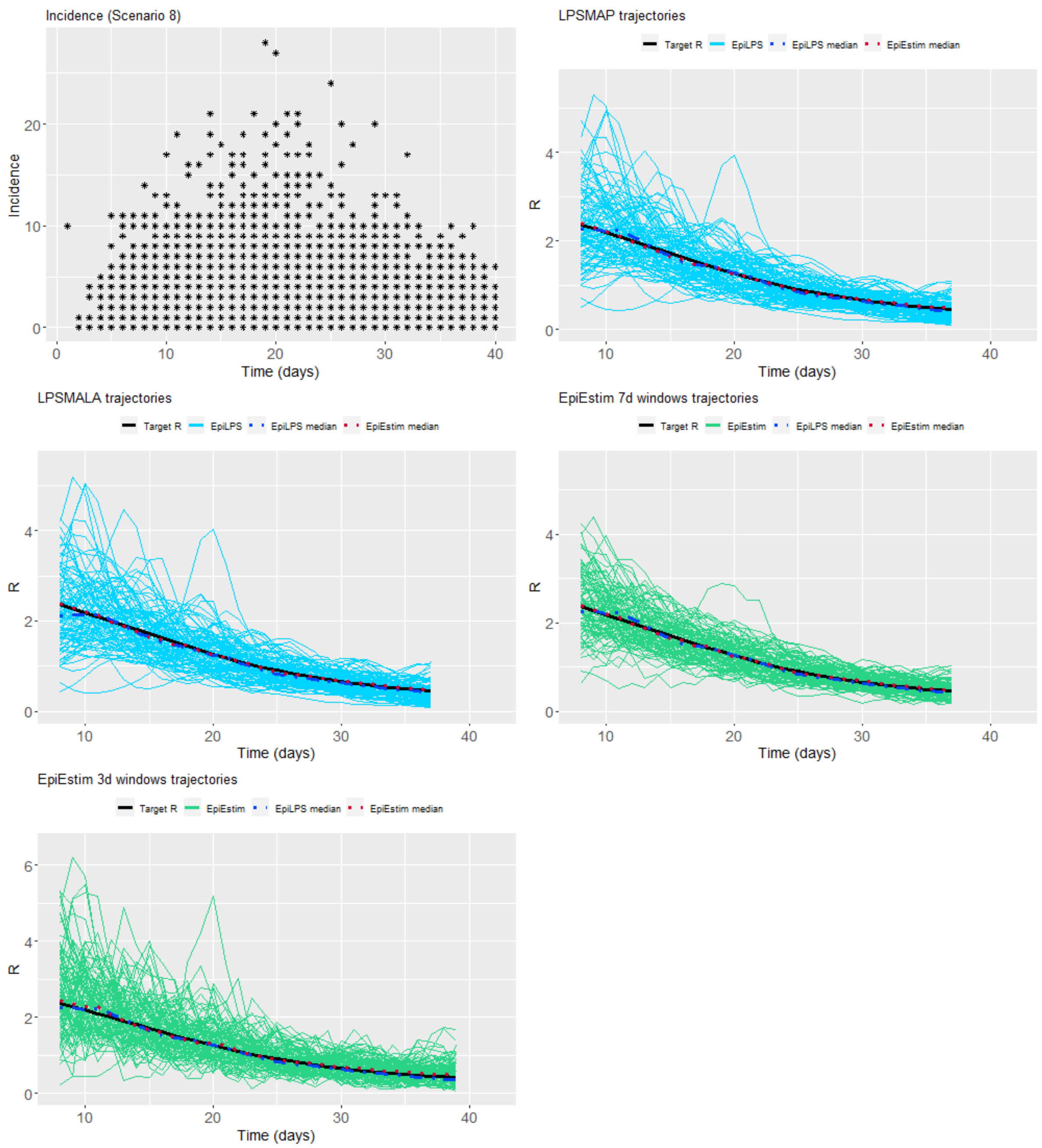

S3 Fig 9. ***Results for Scenario 8 considering S=100 simulated epidemics with a duration of T=40 days and a SARS-CoV-1 like serial interval. Top left panel represents the simulated epidemic curves. Estimated trajectories in light blue are for EpiLPS with LPSMAP and LPSMALA (with a chain length of 3 000 including a burn-in of 1 000) respectively, using K=40 B-splines and a second-order penalty. The green estimated trajectories are for EpiEstim with R(t) reported at the midpoint of the smoothing window for weekly (7d) and three days (3d) windows. Dashed (dotted) curves correspond to the pointwise median estimate of R(t) with EpiLPS (EpiEstim).***

Scenario 9

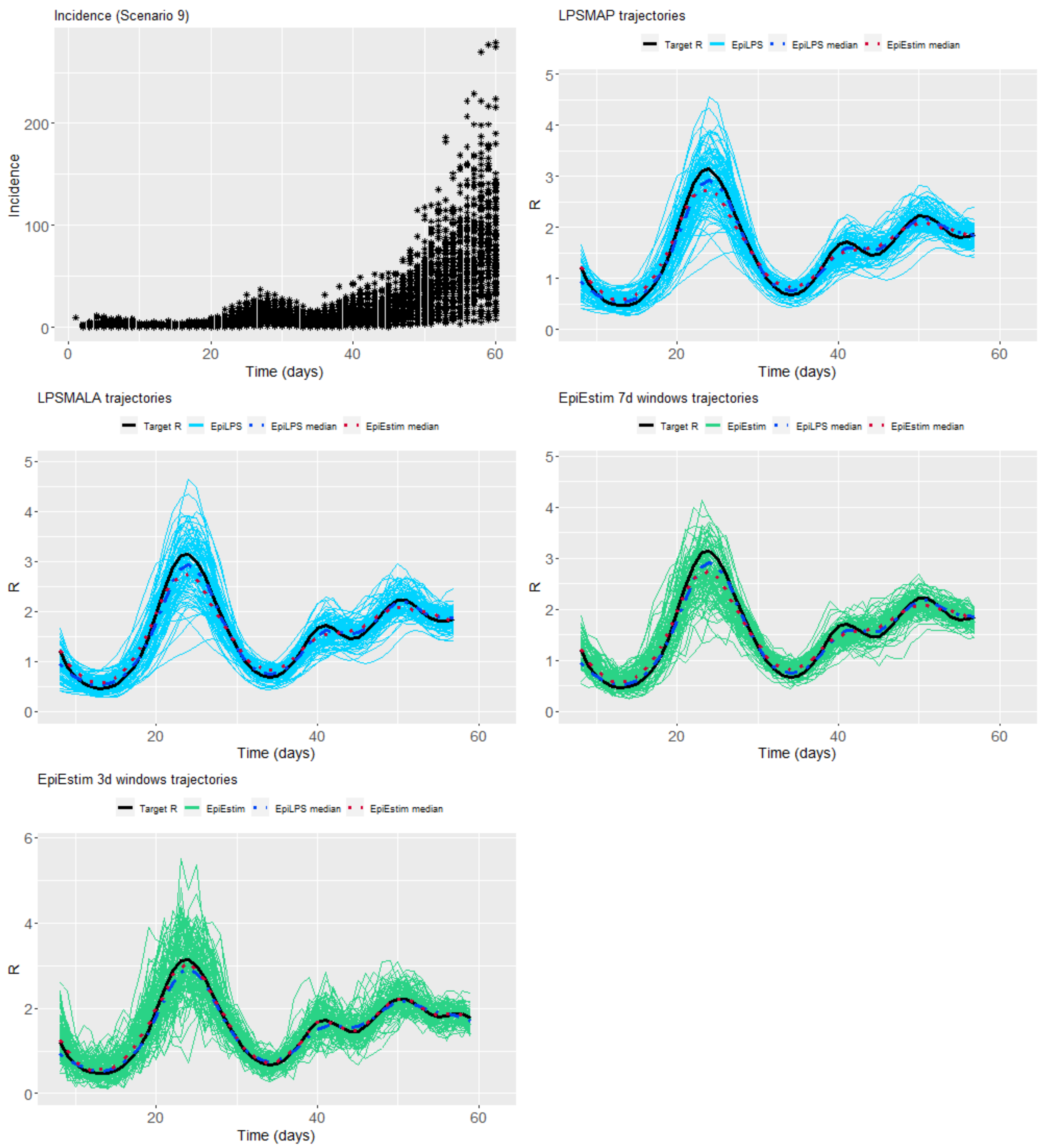

S3 Fig 10. Results for Scenario 9 considering S=100 simulated epidemics with a duration of T=60 days and a MERS-CoV like serial interval. Top left panel represents the simulated epidemic curves. Estimated trajectories in light blue are for EpiLPS with LPSMAP and LPSMALA (with a chain length of 3 000 including a burn-in of 1 000) respectively, using K=40 B-splines and a second-order penalty. The green estimated trajectories are for EpiEstim with R(t) reported at the midpoint of the smoothing window for weekly (7d) and three days (3d) windows. Dashed (dotted) curves correspond to the pointwise median estimate of R(t) with EpiLPS (EpiEstim).

3. Estimation performance for the overdispersion parameter

To have a flavor of the estimation performance of the overdispersion parameter ρ with EpiLPS, we present boxplots of the latter parameter estimate (in log scale) for Scenarios 5-8 computed over *S=100* simulated epidemics and add the true value (red dashed line) of the overdispersion parameter $\rho = 5$ (in log scale) for the sake of comparison. From Figure 11, we observe a small downward bias for each scenario and a bias that is more pronounced for Scenario 7.

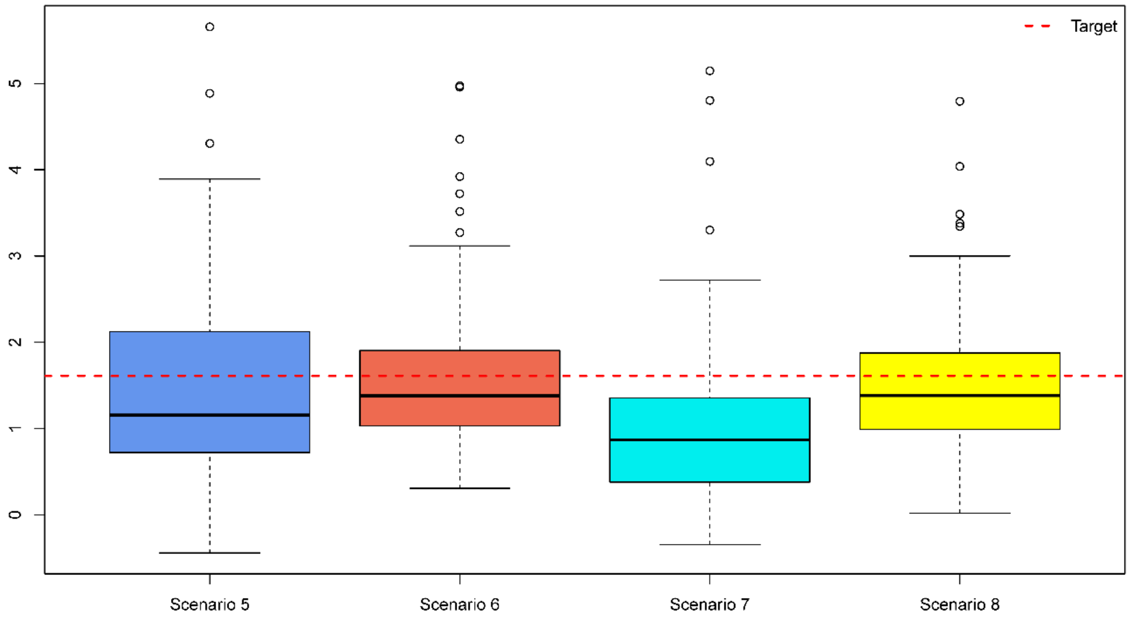

S3 Fig 11. ***Boxplots of the estimated overdispersion parameter with EpiLPS for Scenarios 5-8 computed over S=100 simulated epidemics. A mild downward bias is observed for each scenario.***

4. Are credible intervals affected by different choices of $a_\delta = b_\delta$?

We measure the sensitivity of the credible interval (CI) for R_t (computed by EpiLPS-LPSMAP) with respect to different choices for the prior on the hyperparameter δ. For Scenario 3 and Scenario 9, we simulate S = 50 epidemics and compute the 90% credible interval of R_t at each time step for couples (a_δ = 5, b_δ = 5), (a_δ = 10, b_δ = 10), (a_δ = 20, b_δ = 20), (a_δ = 30, b_δ = 30), (a_δ = 50, b_δ = 50) and (a_δ = 60, b_δ = 60). Figure 12 and Figure 13 show the results for Scenario 3 and Scenario 9, respectively. The computed credible intervals only vary slightly with different couples (a_δ, b_δ), thus showing robustness for EpiLPS-LPSMAP.

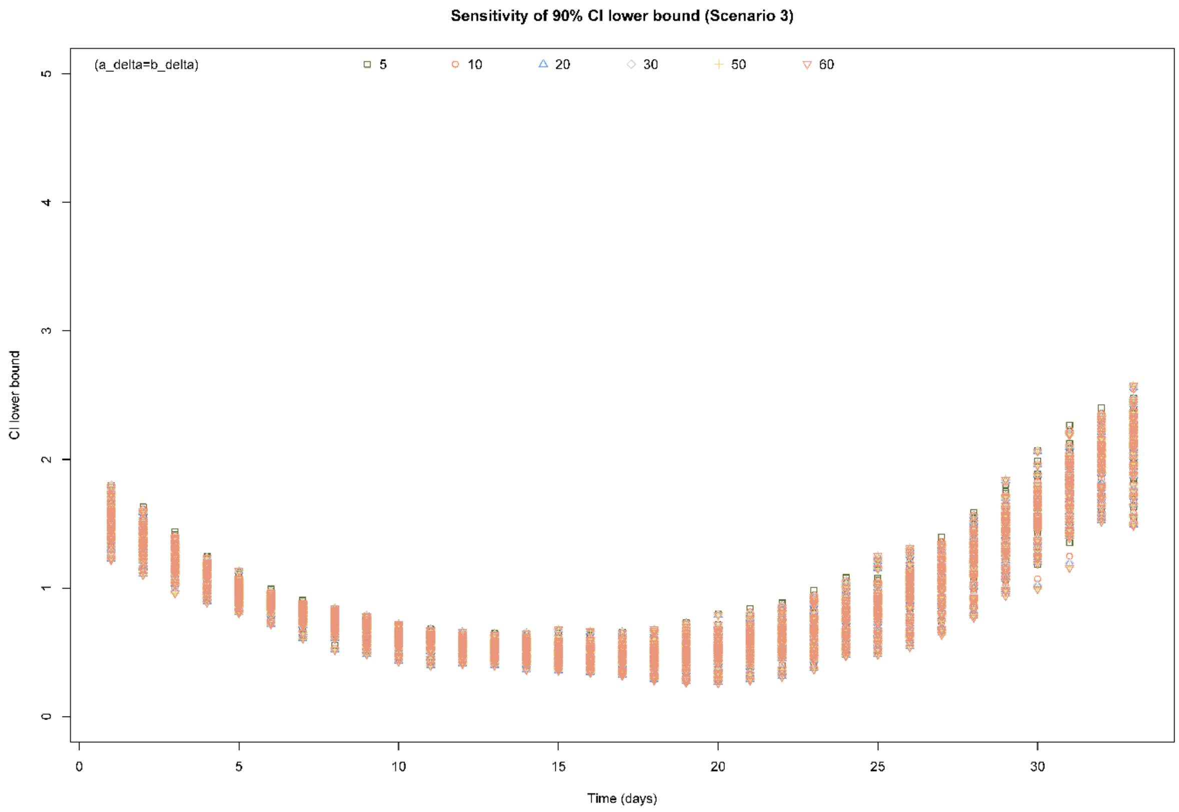

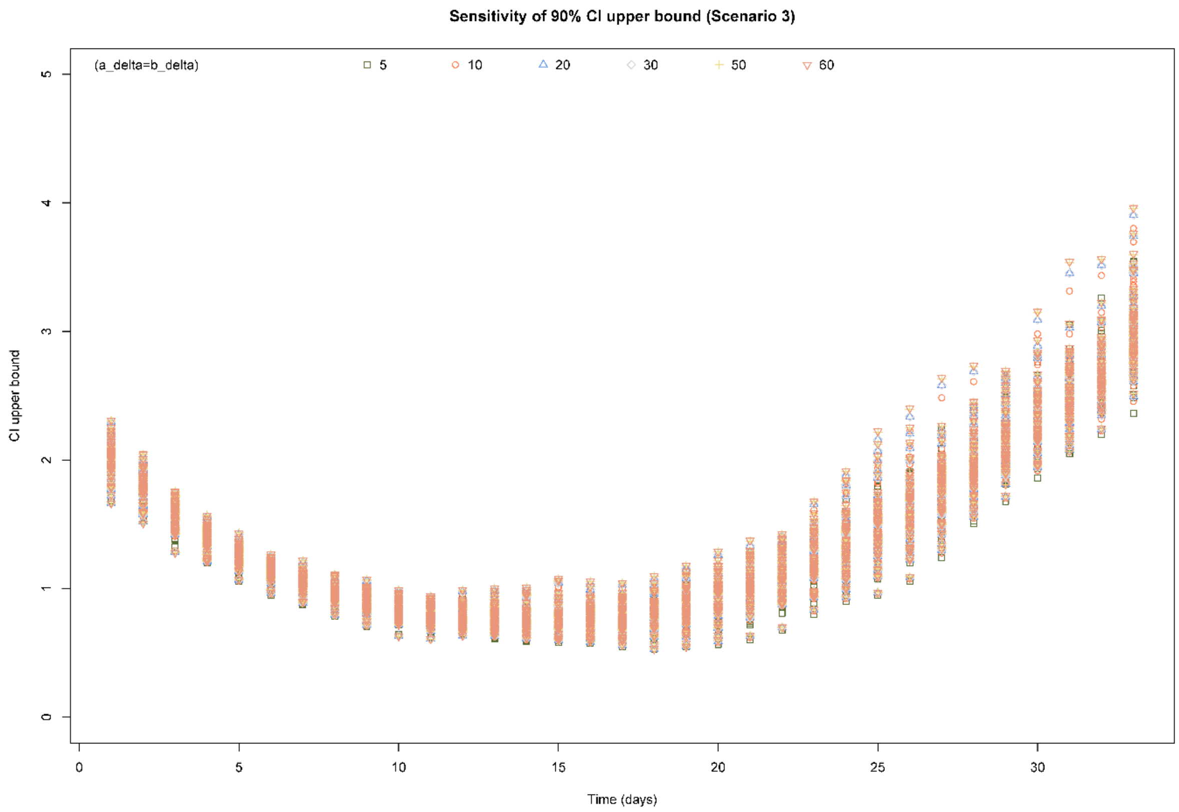

S3 Fig 12. ***Sensitivity of 90% credible intervals for R_t computed with EpiLPS-LPSMAP for different couples (a_δ, b_δ) under Scenario 3. (Top) Lower bound of CI. (Bottom) Upper bound of CI.***

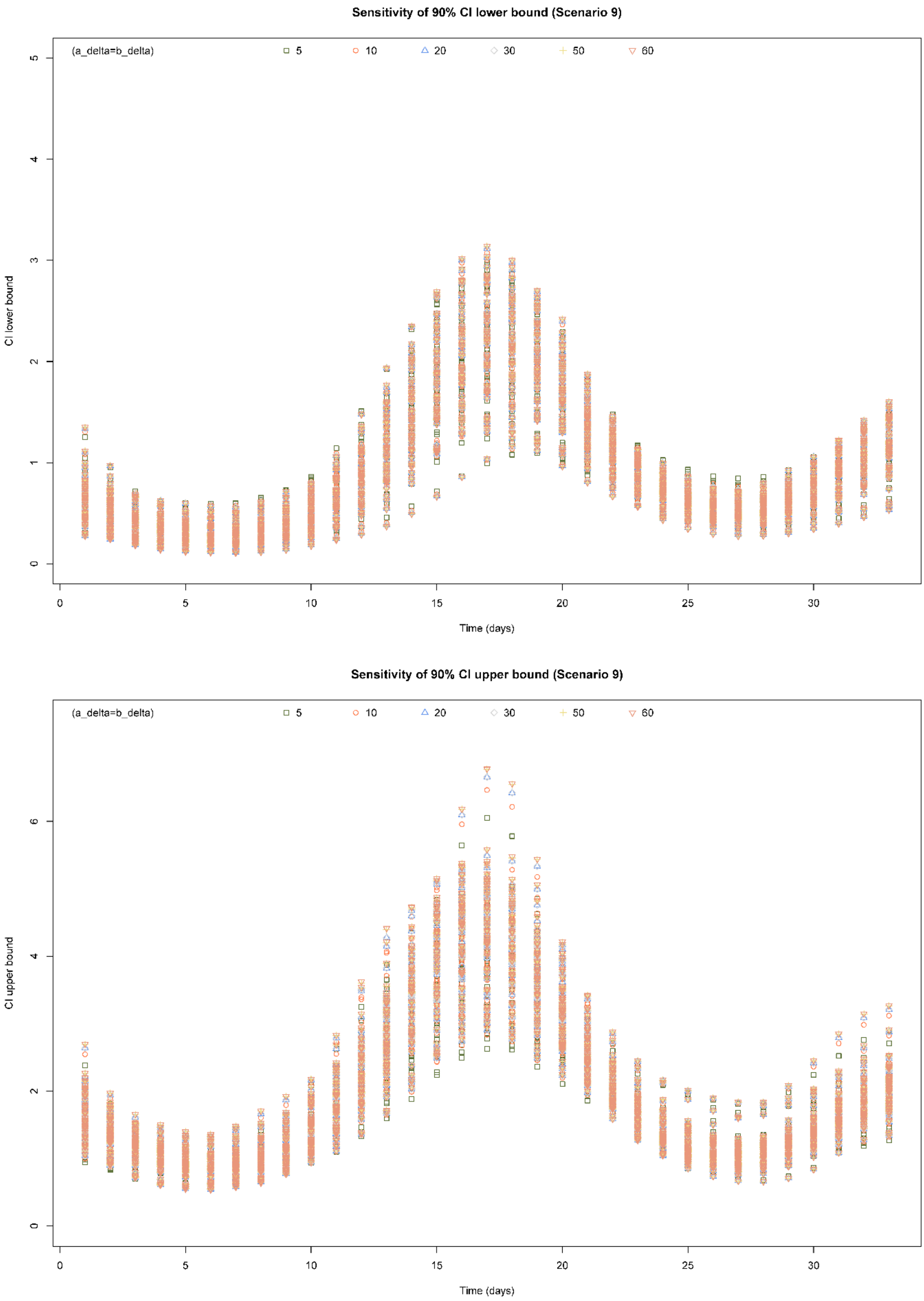

S3 Fig 13. ***Sensitivity of 90% credible intervals for R_t computed with EpiLPS-LPSMAP for different couples (a_δ, b_δ) under Scenario 9. (Top) Lower bound of CI. (Bottom) Upper bound of CI.***

References

1. Cori A, Ferguson NM, Fraser C, Cauchemez S. A new framework and software to estimate time-varying reproduction numbers during epidemics. American Journal of Epidemiology. 2013;**178**(9):1505‑1512. https://doi.org/10.1093/aje/kwt133

2. Gostic KM, McGough L, Baskerville EB, Abbott S, Joshi K, Tedijanto C, et al. Practical considerations for measuring the effective reproductive number, Rt. PloS Computational Biology. 2020;**16**(12):1‑21. https://doi.org/10.1371/journal.pcbi.1008409
