# Supplementary material for: EpiLPS: A fast and flexible Bayesian tool for estimation of the time-varying reproduction number
Source: PLoS Comput Biol. 2022 Oct 10;18(10):e1010618. doi: 10.1371/journal.pcbi.1010618 (PMC9584461; doi:10.1371/journal.pcbi.1010618)
Supplement: Attachment — Submitted filename: Answers_to_reviewers.pdf [file pcbi.1010618.s004.pdf]

EpiLPS: a fast and flexible Bayesian tool for estimation of the time-varying reproduction number

Answers to reviewers' comments

General note to the reviewers

Dear reviewers,

I would like to thank you (on behalf of all co-authors) for the thorough review that was delivered for this manuscript. The comments and suggestions regarding different aspects of the paper (clarification of the methodology, comparisons of EpiLPS with EpiEstim for estimation of the reproduction number $R(t):=R_t$, requests for a more detailed simulation study) gave us the possibility to improve the manuscript in many aspects. As a result, we believe that this significantly revised version gives a clearer picture of the strength of the proposed method with clear explanations regarding the differences with the EpiEstim method. We also recently discovered that a variance correction term in LPSMAP actually improves the accuracy of credible intervals for R_t and included this in the paper. Detailed point-by-point answers to your comments are given below. Amendments made in the main manuscript are highlighted in green color to ease the review process.

Reviewer #1

Reviewer #1: In this study, the authors propose a new method and R package to estimate the time-varying epidemic reproduction number. They use a simulation study to evaluate the performance of their method and compare it to an existing approach (EpiEstim), and present estimated reproduction numbers for historical SARS-CoV-1 and influenza epidemics as well as for SARS-Cov-2 in four European countries. I think the approach is interesting, particularly because it seems to allow for superspreading which few currently available methods do, but I have a number of queries that I would like to see addressed. A caveat: I haven't had time to look into all the mathematical formulas in detail but for those I have looked into in depth they were all correct.

Major comments

Added value of the work

I think in the introduction and throughout the manuscript you could make it clearer from early on 1) why splines are useful (what are they trying to achieve in plain language compared to non spline based approaches) and 2) what is the unique aspect that your approach tackles (I think the use of a negative binomial likelihood, which I think you should emphasize more and again explain in plain English why it's a useful feature, e.g. to model superspreading). I have to say I was very excited by the prospect of this paper precisely because I was hoping it would address gaps in the literature on

estimating the reproduction number in the presence of super-spreading; but this doesn't seem to be specifically addressed in the end, particularly in the simulation study which used a Poisson offspring distribution (see further comments below). So in summary, could the authors more clearly explain what makes their method different / better than others and could they show in their simulation study examples illustrating this more clearly?

- We have revised the text throughout the manuscript to explain clearly 1) why the negative binomial likelihood is desirable for analyzing infectious disease count data and 2) why the chosen penalized B-splines setting is attractive within our model. We added these explanations in Section 2.1 just after introducing the mathematical formula of the negative binomial distribution and after Equation (2) for motivating the spline approach. We understand the gap existing in the literature on estimating R_t in presence of superspreading, however EpiLPS was not originally meant to directly address this issue, at least not in the *biological* sense of superspreading. Indeed, we cannot really talk about an *offspring* distribution here, as our data generating process (DGP) in the simulation study is not anchored on branching process theory. In fact, the DGP assumes a functional form for R_t and simulates the incidence case counts from a parametric distribution (now the negative binomial instead of the Poisson distribution assumed in the first submitted version of the paper) based on the renewal equation. The rationale for choosing such a DGP is that one is able to make comparisons (and so compute performance metrics) between estimated R_t at a given time step t and the ''true'' R_t values over multiple simulated epidemics. This is harder to achieve when we simulate data from a biological model (as target R_t values will change from one simulated epidemic to the other). Note also that this is also what Cori et al. (2013) did in their simulation study and it is therefore more suitable to make meaningful comparisons between EpiLPS and EpiEstim. As in Cori et al. (2013), the reproduction number provided by EpiLPS can only be interpreted in an "average" sense (for a homogeneous group) rather than in an "individual-based'' sense (heterogeneous group). We can however argue that EpiLPS captures superspreading to some extent in the sense that the secondary cases generated by superspreaders are reflected within the case incidence data used as an input in the EpiLPS methodology. Finally, the negative binomial distribution in the DGP is motivated by the fact that (1) variation in reporting and (2) heterogeneity in secondary transmission (i.e. "superspreading") can result in a process that is not simply Poisson.

Simulation study

I thought the simulation study could be hugely improved to better demonstrate the contexts in which the method performs well / better than others.

- The simulation section has been improved and totally redesigned so as to make comparisons between EpiEstim and EpiLPS clearer.

First, why did you simulate from a Poisson and not a Negative binomial (see my comment above)? This seems very bizarre since I think this may be the main strength of your approach. I would suggest you expand your simulation study to demonstrate how well the method performs at various levels of superspreading / overdispersion. I would anticipate that benefits over for example EpiEstim may appear more clearly in the presence of overdispersion, which is not accounted for in most other approaches for estimation of R.

- We now simulate the incidence cases from a negative binomial distribution with scenarios assuming different degrees of overdispersion. We added a new file ''Supplementary

information 2'' in which we give a detailed account of the data generating process. We also summarize all the estimated R_t trajectories (and simulated epidemic curves) for all the simulation settings in these supplementary materials, as there are too many figures to be included in the main manuscript. The benefits of EpiLPS over EpiEstim appear more clearly now, especially in terms of coverage of credible intervals.

Second, how did you choose the serial interval for each scenario? It seems weird to have different (rather ad hoc) ones for each scenario as it's then hard to tell whether differences between scenarios come from the Rt profile or the change in SI. I would suggest using a couple of SI (maybe matching diseases e.g. flu and ebola for a short and longer SI?) and considering both SIs for each scenario.

- Thank you, this is indeed a very good point. We now have rewritten Scenarios 1-4 with an influenza-like serial interval, Scenarios 5-8 with a SARS-CoV-1 like serial interval (and additionally one more scenario with a MERS-CoV like serial interval), to make our SI choice less ad hoc. The influenza serial interval is shorter in length than the SARS serial interval, so we have a short and longer SI as you suggested. The detailed description of the chosen serial intervals and their associated reference in the literature are provided in Table 1 of the main manuscript. Scenarios 1-4 and 5-8 have the same underlying "true'' R_t functions and differ with respect to the serial interval distribution.

Third, how did you choose the values of K = 20 and second order penalty for the simulation study? Please consider alternative values (as you do with the window length for EpiEstim); I anticipate results may be quite sensitive to this choice.

1. The philosophy behind P-splines is to use a "large" number of B-splines basis function and to counterbalance the resulting flexibility of the model with a roughness penalty on the coefficients of contiguous B-spline parameters. There is no consensus on what "large" means here, but as Eilers & Marx (2021) put it: *"…even 1,000 B-splines will work well…"*. In our new simulation settings, we use K=40 and this choice is arbitrary as well, but we find it large enough for the modeling purposes of this article. The argument that the fit is sensitive to the choice of K would go against the P-splines philosophy. In Supplementary information 2, we add a sensitivity analysis of the R_t fit with LPSMAP in two different scenarios. This sensitivity analysis confirms that the fit has a negligible sensitivity to the choice of K as long as K is large.

2. It is actually very interesting that you establish a relation between K in our model and the time window length in EpiEstim. The window length in EpiEstim is the main driving force of smoothness (larger windows → smoother estimate, smaller windows → undersmoothed estimate with too much noise/variation picked up). In EpiLPS it is the value of the smoothing parameter λ that determines smoothness, not the number of B-splines K. Therefore, we think that the right analogy to be made between EpiLPS and EpiEstim is by comparing the key drivers of smoothness for the fit (the time window and λ). And here, our EpiLPS approach has a competitive argument in the sense that λ is naturally estimated within the model (via a maximum a posteriori argument (LPSMAP) or MCMC (LPSMALA)). In other words, with EpiLPS we do not need to care about the trade-off between an over- or under-fitting phenomenon induced by a free parameter (the time window in EpiEstim), as EpiLPS solves this tradeoff intrinsically via a Bayesian estimation of λ.

Finally, for EpiEstim results, how did you interpret these? i.e. for the 7 day time window, what "true" value of Rt (used for simulation) did you compare the 7 day average for? Rt at the window midpoint? The start? The end? It is very suspicious that in all scenarios (Figures 1-4, but maybe clearest Figure 3

the EpiEstim estimates seem to be "delayed", as if you are not comparing like to like. And what priors did you use for EpiEstim? Also, what results would you obtain if you used 1 day windows for EpiEstim?

- The performance measures in the simulation study (Bias, MSE, coverage probability and credible interval width) are computed by comparing the value of the reproduction number at time step t with the estimate of R_t at the end of the time window for EpiEstim. Cori et al. (2013) explain this (in their Web appendix 3) by saying that `` *...the estimate of R over a chosen time window can only be obtained at the end of that window, since it requires the observation of incident cases over the whole window.*" This explains why EpiEstim estimates appear lagged. This lag can be reduced by considering smaller time windows (see our new section in the simulation study discussing this) but at the cost of more wiggly fits. Following your suggestion, we also now add the results with daily sliding windows for EpiEstim (see Table 2 and Table 3).

Potential limitations of the work

The authors claim that "alleviating the window size assumption (as in EpiLPS) can be of interest" but they introduce a number of other parameters, including K, the number of spline knots, the choice of which is I think as arbitrary as that of the time window in EpiEstim albeit less easy to interpret. The authors should present sensitivity of their results to these parameters and add some discussion.

- A sensitivity analysis to the choice of K (and hence the number of spline knots) has been added in Supplementary information 2 showing that this choice has a negligible effect on the fitted R_t curve. The number of B-splines K is in fact arbitrary (and has no direct interpretation), but the P-splines philosophy advises a recipe with a "a large" number of B-splines and a penalty to act as a counterforce to the resulting flexibility. Our analyses in the paper use K=40 B-splines (which is more than enough, but more could be used if desired). We added a discussion on the sensitivity of the R_t fit with respect to K in Section 3.2.

Ability to detect sudden changes in Rt over time: "We assume that μ(t) evolves smoothly over the time course of the epidemic" ◊ Does this mean the method would not be very appropriate to estimate rapid changes in R(t) for example the reduction in R associated with a lockdown or a similar sudden control measure? This is what your simulation study suggests, and should be added to the discussion, along with more discussion on the contexts in which the authors expect their method to be particularly useful.

- EpiLPS is able to track rapid changes in R_t and is even able to react "quicker'' than EpiEstim with weekly sliding windows as shown in our simulations. The reason is that EpiLPS provides a global smoother (i.e. smoothing is done over the entire domain of the epidemic curve at once). We added a complete discussion about this in Section 4. The lag of the R_t estimate with a weekly sliding window in EpiEstim can be reduced by considering a time window of shorter length (e.g. three days or one day) but at the cost of more wiggly and abrupt estimates capturing more variation than necessary. This trade-off between lag and noise is absent in EpiLPS.

In figure 1 your approach seems able to detect a drop in Rt before it actually happens ◊ how is that possible? This suggests to me that estimates of Rt will be informed by data from later (after t), which would potentially preclude real time use. Could the authors clarify and discuss this.

- In fact, EpiLPS delivers a global smooth curve by looking further backwards than the window width assumed in EpiEstim. Note that a reference in our paper (Gostic et al. 2020) discuss this

scenario and explain that smoothing approaches can either “lead or lag’’ the true R_t value. In particular, note that reporting EpiEstim values at the midpoint of the time window (instead of at the end of the window) will result in the R_t estimate to lead (i.e. the estimated curve will start dropping before the drop actually happens) the “true” R_t value as in our case. We do not think that this should preclude real time use, but agree that EpiLPS does not provide real-time estimates of R_t in the same sense as the Cori method. See discussion in Section 4.

What is the effect of the different priors and hyperpriors?

- A sensitivity analysis with respect to the choice of $a_\delta = b_\delta$ (as in Jullion and Lambert 2007) in the hyperprior for δ has been added in Supplementary information 2. Results show that estimates of R_t are robust to the different choices considered for a_δ and b_δ. This is discussed in Section 3.2 in the paper.

A lot of the methodology is based on the Laplace approximation and I would have liked to see some evidence and/or discussion about the extent to which this approximation is going to be valid in the context of the renewal equation.

- We now describe the two key roles of the Laplace approximation in Section 2.2. We also add a reference that has already shown the strength of Laplacian-P-splines (LPS) in modeling count data (specifically in the context of generalized additive models). As LPS is valid in the context of modeling count data, it will also remain valid for modeling R_t since, essentially, the estimated average incidence of cases (obtained with LPS) is plugged into the renewal equation.

Generally more discussion should be added on the caveats / subjective choices, and which types of epidemic contexts do we think this method will perform well / better than other methods.

- We give more general discussions about the comparison of EpiLPS with EpiEstim through the text and especially in Section 4. In addition, we discuss the caveats and subjective choices and we also implemented several sensitivity analyses (with respect to the number of B-splines K, and the δ hyperprior).

Methodological clarifications

In section 2.4 I don’t think rho (the overdispersion parameter) appears at all. Could you please clarify the dependency on rho in this section?

- The dependency on the overdispersion parameter rho has been clarified in this section just below Equation 11.

Section 2.4.1, can you state more clearly what phi1, etc are; I assume phi1 = P(SI = 1)? Please define k more clearly. You should also highlight an assumption which is that p(SI <= 0) = 0 = p(SI > k). Please also more clarify in equation (9) whether y_t is the incidence of infections or symptomatic infections. In equation 9, please clearly state the values of s over which the sum is taken

- Thank you for this comment on mathematical notation. We give a clearer mathematical definition of the serial interval distribution and add the assumptions when SI<=0 and SI>k. Equation (9) has been rewritten clearly with the formula now including the values of s over which the sum is taken.

Does the method lead to estimating the overdispersion parameter rho? If so, what estimates did you get for SARS-CoV-1 and 2 and flu and how do they compare with estimates from the literature obtained from analysing contact tracing data for example?

- The EpiLPS tool is primarily designed to estimate the reproduction number by smoothing the mean incidence counts over a given considered period and from there infer the reproduction number via the renewal equation. The overdispersion parameter rho is thus a nuisance (hyper)parameter in our model and its estimate is inferred from the whole set of observed case incidence data. As such, the estimates we obtain for rho based on the aggregated case incidence data are quite different from the individual-based models estimates for rho obtained from analyzing contact tracing data. For obtaining estimates of approximately the same magnitude, one could extend the model in the philosophy of Johnson et al. (2021) to directly account for superspreading, but this would lead to an entirely different model. It is therefore important to distinguish between overdispersion parameters for case counts (due to reporting and heterogeneity in transmission) and for the number of secondary cases (offspring distribution). These two parameters are not the same and are often being confused. Even though superspreading can lead to overdispersion in case counts, an overdispersion of case counts does not necessarily imply superspreading.

Related to my point about sensitivity to the number of spline knots K, I wonder how robust rho estimation would be to different choices for K, as I suspect it is hard to disentangle variations of Rt in time (the magnitude of which will be driven by K) from overdispersion (rho). I am not entirely sure these two are identifiable and would have hoped the authors show more clearly if this is true or not.

- A sensitivity analysis in Supplementary information 2 shows that estimation of R_t is actually quite robust to different choices for K (provided one chooses K large enough). The magnitude of the variations of R_t in time are actually not driven by K, but by the value of the smoothing parameter λ, the key driver of smoothness in EpiLPS. Therefore, there is no reason to think that there is an indentifiability issue between the estimate of R_t and the estimation of the parameter rho (overdispersion) which is actually a nuisance parameter in our model that is included within our hyperparameter vector.

Comparison between your two methods and with EpiEstim

It is not clear how or when one should use LPSMALA versus LPSMAP; I would recommend that this is discussed further and that results of the two approaches are compared more systematically in figures. For example why did you use LPSMALA for SARS and Flu but LPSMAP for covid?

- LPSMAP and LPSMALA essentially have the same accuracy (as shown by the performance measures in the simulation study) when it comes to estimation of R_t. However, LPSMALA (being a (gradient-based) MCMC algorithm) requires a much larger computational budget. We therefore recommend using LPSMALA for epidemics with shorter durations (like in the SARS or influenza examples) and LPSMAP for longer durations (like in the Covid-19 application) to obtain quick results. If computational budget is not an issue, LPSMALA can of course also be implemented on outbreaks with long duration. We now give complete figures of the LPSMAP and LPSMALA trajectories (Supplementary information 2) in the simulation study and make the recommendation for LPSMAP/LPSMALA in Section 4.

In Figure 5: how do these compare to the EpiEstim estimates? Cori et al. used 5 datasets but you only looked at 2 here, was there a specific reason? Could you include them all?

- The code for overlaying the EpiEstim estimates in Figure 5 has been added in the Github repository (see link in the Data availability statement). Moreover, we directly added the Cori estimates to Figure 6 for a direct comparison with EpiLPS in the application of SARS-CoV-2. Yes, we looked at only 2 historical datasets while Cori et al. (2013) considered 5. We wanted to focus on two historical outbreaks and one more recent outbreak (COVID-19). For the sake of space, we therefore did not tackle all the datasets that Cori et al. considered. We are currently working on further documentation on EpiLPS (and a dedicated website https://epilps.com) where more datasets will be used to illustrate the EpiLPS tool.

Implementation

Could the authors give some indication of computing time please.

- Computing times are now reported for different (T,K) couples in Section 3.2.

Also, it is great that the R package is available on CRAN, but a shame that a vignette is not available. I would suggest that the authors use the SARS and flu examples for example as a basis for a vignette which would allow users to get started (I did notice some code is available to reproduce the results but the readme is not very informative so I think a vignette would be much better; I was using the link from the data availability section). The example in the epilps function runs well and fast (even with the LPSMALA method which is encouraging but I haven't checked convergence was achieved).

- The link that you used from the data availability section has a README file that is in fact not very informative as its purpose was mainly to add code to reproduce the results of the article. Note however that I have a dedicated Github repository for EpiLPS (https://github.com/oswaldogressani/EpiLPS) which is much more informative and allows to get started with the routines. I plan to dedicate future work on explaining in detail how the EpiLPS algorithm works and eventually add a vignette later on.

Minor comments

Intro: I would avoid talking about "severity" of the outbreak in the context of the reproduction number as this may be confusing since R only measures transmissibility, not severity.

- The term ''severity'' has been replaced by ''transmission potential''.

Equation 4: please clarify the notations, is L(theta, rho, D) the likelihood?

- Yes , it is the likelihood and it is now clearly stated just below Equation 4.

Do you really need to have both equations 12 and 13?

- No indeed, this is redundant information. We removed Equation 13.

Reviewer #2

Reviewer #2: This paper presents a variant of the by now well known Cori et al (2013, 2021) approach to R_t estimation in an infectious disease outbreak based on smoothing the incidence count time series with splines.

The approach has the same basic problems as similar approaches to R_t, namely not accounting for cases not included in the time series of reported cases, delays in reporting, mixtures of differently infective strains, different incidences in different population groups.
Furthermore, a fixed serial time distribution is assumed (note that not all notified cases are due to symptom appearance and that recent research points to generation/serial times changing during an epidemic due to interventions and public behaviour...).

All this notwithstanding, R_t has turned out to be an important summary of population disease progress, used by decision makers, media and the public.

The paper is essentially concentrated on the computational part of R_t estimation as noted by the authors in the paragraph spanning pages 4-5, not on innovation of the concept of R_t itself. In fact, 14 pages out of 25 are essentially devoted to mathematical/statistical details of the used algorithms. Further 4 pages show the results of simulations of the proposed method compared to the EpiEstim method and finally there are 2 pages showing the results of the proposed new method applied to some real data.

Thus, the paper advocates use of the EpiLPS method as being slightly better than EpiEstim based on some simulations and with the theoretical advantage of not having to choose widths of moving windows in data. Perhaps, for a journal like PLoS Comp Biol, a much shorter paper with all the maths moved to an electronic appendix, would have been sufficient and quite adequate.

- Thank you for your suggestions. We restructured the paper and added more emphasis on the comparison between EpiLPS and EpiEstim. This gives a more in-depth view about what truly distinguishes EpiLPS from EpiEstim. The technical details that were in the appendix within the main article are now available in Supplementary information 1. We also give more details regarding the simulation study in Supplementary information 2 to reduce the length of the paper. As such, only the math that is essential in the presentation of the EpiLPS methodology is present in the main manuscript.

Some minor comments

On several occasions, R_t is called a "metric" in conflict with the usual mathematical meaning of the word.

- We replaced the term ‘’metric’’ by ‘’parameter’’ or “statistic”.

In section 2.1, 3rd line, "infections" are called "contaminations", which may not be the most suitable synonym...

- We replaced ‘’contaminations’’ by ‘’infections’’.

The four simulation scenarios are reasonable, but why are the serial interval distributions different?

- Thank you. We have completely restructured the simulation scenarios with influenza, SARS-CoV-1 and MERS-CoV like serial interval (SI) distributions. This makes our SI choice less ‘‘ad-hoc’’ and more meaningful for the simulations.

Section 3.1 shows the proposed method seemingly having better adaptability to the "true" R_t but the comparison is with EpiEstim used with two fixed choices of window. Which choices of window widths have actually been advocated by EpiEstim users?

- To our knowledge, weekly sliding windows seem to be a popular choice. (It is also the default in the EpiEstim package). We added a daily time window. With daily sliding windows, the estimated R_t trajectory with EpiEstim is really wiggly as it captures a lot of variation. We have reported the entire set of trajectories of the simulation study in Supplementary information 2, where the effect of changing the sliding windows in EpiEstim is now clearly visible. Having used daily windows in the simulation study also adds to the interpretability for the difference between EpiLPS and EpiEstim.

Reviewer #3

Reviewer #3: This is a very technical paper describing a method for estimating the reproduction ratio from epidemic data. The method uses well known approaches (MCMC, laplace approximmation) that are combined and provide an improvement in computation time.

1 - The method cannot be presented as "real-time". Real time should be reserved for methods really attempting to estimate "in real time", that is characterized by estimating R(t) from data up to time t. Here, as is common in such presentations, the authors look at a global estimate obtained from the whole data. The R(t) estimate obtained from the renewal equation is actually informed from the global spline smoothing.

- This is indeed true as we look “at a global estimate obtained from the whole data” and not just the most recent estimate. We have therefore changed the title to: “EpiLPS: a fast and flexible Bayesian tool for estimation of the time-varying reproduction number”. We discuss more in detail in what respect the EpiLPS smoother is a global smoother and what are the consequences in terms of inferring the reproduction number (see Section 4 and Supplementary information 2).

2 - In the end, it is not very clear if the method should be prefered to the EpiEstim method. It is mentioned that the coverage is better, but from what I read on EpiEstim they are using a window and estimating an average R(t) over the window, with a corresponding confidence interval. So In the end, I'm not convinced that the comparison as presented here is fair, because the confidence interval is for R(t) in your approach and for an average R(t) in the other case.

- Thank you. We now describe in detail how the ‘‘true’’ R_t in the simulation study is compared with the EpiEstim R_t, which actually coincides with the R_t at the end of the window size. Actually, the comparison is fair in the sense that the ‘‘true’’ R at time t is compared to the

estimated R given by EpiEstim and EpiLPS at time t. So, essentially, we are comparing two meaningful point estimates of R_t. In our new simulations, we compare EpiLPS with three choices of the time windows in EpiEstim (weekly, three days and daily windows) to have a more complete set of comparisons.

3 - There is one strange feature in the estimate seen in fig 2 for example : the R(t) starts to decrease before the date of intervention. Do I have to tell you that this feature is always unwelcome? Indeed, this is exactly what will be used to say that "evidently" the intervention was not responsible for the decrease in R(t) because "it started before the intervention". How could it be possible to change the prior on the smoothing parameter automatically to change this?

- The reason why the R_t estimate with EpiLPS drops before the date of intervention is because it is a global smoother. At the moment one uses the EpiLPS tool for this example, i.e. at time T=40 of the epidemic, the drop in R_t arising at day t=20 is actually captured by data before and after day t=20, so that our global smoother reacts a bit early. A similar example has been discussed by a reference we give in the paper (Gostic et al. 2020). By suggesting to report the EpiEstim estimate of R_t in the middle of the time window, the latter reference shows graphically that with EpiEstim it is also possible to have an estimated R_t that drops before the intervention truly happens. In Section 4, we give more details regarding the behavior of our EpiLPS global smoother and do not call it a real-time approach anymore. We advise against changing the priors to obtain a different fit of R_t as this would go against the Bayesian philosophy (i.e. twisting our priors to get the desired result would not be honest). This would not work anyway as we have shown that EpiLPS estimates of R_t are robust with regard to prior choices (see Supplementary information 2).

4 - I suppose that running the method "in real time" during the course of an epidemic would lead to changes in the estimates over the whole period because there would be changes in the smoothing parameter. I would find it rather difficult to recommend the method if the "past" estimates would change as time passes.

- Thank you, I think this relates to my point raised above. We have added an illustration about how estimates of R_t in the past change when applying EpiLPS sequentially over the time course of a simulated epidemic (Supplementary information 2). We think that recommendability of EpiLPS (from a stand-alone point of view as well as compared to EpiEstim) should not be made based on whether past estimates change or not, but based on whether the past R_t estimates remain close to the target R_t. For instance, when receiving data about incidence cases on a daily basis, a feature often encountered is that the numbers are adjusted (up to two or three days in the past), so that even when applying EpiEstim, the estimated R_t trajectory will be different in the past.

5 - Are the confidence intervals "point-wise" or "trajectory-wise"?

- The constructed credible intervals are ‘‘pointwise’’ both in EpiEstim and EpiLPS, we changed the figure captions to make this explicit.

6 - The method boasts using the NB formulation, but it is not shown how this makes the method more relevant. For example, was there evidence in the examples that this was required? what was the difference between assuming NB or Poisson?

- We give a more detailed description that motivates the benefits of assuming a negative binomial (NB) distribution for the case incidence data. Also, our simulations are now drawing incidence data from the NB distribution instead of the Poisson distribution in the data generating process. It thus gives a clearer picture when comparing EpiLPS (which uses a NB likelihood) and EpiEstim (which uses a Poisson likelihood). The performance of both approaches has been measured in nine different scenarios and results have been reported in Tables 2 and 3. Using the NB formulation also allows us to get an estimate of rho (the overdispersion parameter) and hence to quantify overdispersion.

7 - I'm surprised that the daily pattern is not considered at all. In most countries, there was a strong such pattern. How comes the spline does not try to adapt here to this pattern, since the prior seems to be favoring wiggliness?

- The daily pattern (i.e. daily sliding windows) is now considered for EpiEstim in the simulation study. In EpiLPS, even if we have a prior that favors wiggliness, note that it is the posterior penalty parameter that is key in determining the degree of smoothness of the fitted curve. EpiEstim with daily sliding windows are shown to capture ‘‘too much’’ noise or variation. There is a trade-off between over- and under-smoothing in EpiEstim that is settled by the (free) choice of the time window width. The advantage of EpiLPS is that this trade-off issue is dealt with internally by P-splines.

8 - The model for using the NB does not really account for individual variation in transmission, where one would assume that the offspring of each case is Xi = NB(R(t), rho). In this case, a more sensible description is i(t) = Sum Xi = NB(R(t) i(t-1), rho i(t-1)) assuming here for simplicity a generation time of one time unit. That is the dispersion parameter increases with incidence. This would be of interest to see whether you can accomodate this description in here since it is much more relevant.

- In fact, this is a very interesting and important point. Individual variation in transmission is not accounted for in EpiLPS as it is not a model motivated by a branching process, but rather a global smoothing model over the entire epidemic curve. As such, we try to capture variation in case counts by using a hyperparameter that has no mechanistic or biological meaning. Adapting individual variation in the model would completely change its structure and we therefore consider it as an interesting path (among many others) to extend and enrich the EpiLPS tool in the future.

References

Cori, A., Ferguson, N. M., Fraser, C., and Cauchemez, S. (2013). A new framework and software to estimate time-varying reproduction numbers during epidemics. *American Journal of Epidemiology*, 178(9):1505–1512. https://doi.org/10.1093/aje/kwt133

Gostic, K. M., McGough, L., Baskerville, et al. (2020). Practical considerations for measuring the effective reproductive number, Rt. *PLoS Computational Biology*, 16(12), e1008409. https://doi.org/10.1371/journal.pcbi.1008409

Johnson, K. D., Beiglböck, M., Eder, M., et al. (2021). Disease momentum: estimating the reproduction number in the presence of superspreading. *Infectious Disease Modelling*, 6, 706-728. https://doi.org/10.1016/j.idm.2021.03.006
