# Supplementary material for: EpiLPS: A fast and flexible Bayesian tool for estimation of the time-varying reproduction number
Source: PLoS Comput Biol. 2022 Oct 10;18(10):e1010618. doi: 10.1371/journal.pcbi.1010618 (PMC9584461; doi:10.1371/journal.pcbi.1010618)
Supplement: Attachment — Submitted filename: Answers_to_reviewers_v2.pdf [file pcbi.1010618.s005.pdf]

EpiLPS: a fast and flexible Bayesian tool for estimation of the time-varying reproduction number

Answers to reviewers' comments for Major Revision 2

General note to the reviewer

Dear reviewer,

I would like to thank you (on behalf of all co-authors) for your detailed comments and helpful suggestions in this second review round. It permitted us to give a broader perspective to the paper by comparing EpiLPS with EpiEstim following the recommendation of Gostic et al. (2020) to report the estimated Rt at the midpoint of the time window. This new comparison reveals that EpiLPS still outperforms EpiEstim even under the latter best practice and, as such, makes a stronger case for EpiLPS. Moreover, we make a clearer statement as to how the EpiLPS tool should be used, namely that it is a robust tool for retrospective analysis and is by nature only partially real-time. You can find the point-by-point answers to your comments below. Amendments made in the main manuscript are highlighted in color to ease the review process.

Reviewer #1

Reviewer #1: I appreciate the changes made by the authors, which I think have improved the manuscript, but I still have critical concerns about the work presented.

My three main concerns are as follows.

First, much of the paper is focused on a comparison with EpiEstim, but I think the comparison is not made in a fair way. Indeed Rt is compared to the Rt EpiEstim would estimate on the time window]t-w;t] where w is the width of the estimation window. EpiEstim authors do explain this is the only way to have a fair evaluation of the real-time ability to recover Rt. But it does mean the estimates are "lagged". If you are not tied by the real-time assumption, then a much better estimate of Rt is that obtained from EpiEstim on the time interval centered around t: [t – w/2; t + w/2]. This does require data after t but will no longer result in a lag. Since the method presented here is not applicable in real-time, and uses data after t, I think a much fairer baseline comparator for this new method would be this non lagged, non-real-time version of EpiEstim. I don't think the article is publishable without showing this as I believe the performance of that version of EpiEstim will be much better. Hopefully the new methods developed by the authors will be even better (and looking at the figure results, I think they may well be) but this needs to be demonstrated.

- Thank you for this insightful and detailed comment. We have restructured the simulation study section and added two additional subsections discussing the comparison between EpiLPS and EpiEstim when Rt is (1) reported at the boundary of the window [t-w; t] and (2) at the midpoint of the window, i.e. [t-w/2; t+w/2] following the best practice recommendation of Gostic et al. (2020). We have run the simulations for all scenarios (Scenario 1- Scenario 9) and compared EpiLPS with EpiEstim estimates reported at the window midpoint. We discuss

the results in Section 3.3 and show all the performance indicators and related figures of the estimated Rt trajectories in Supplementary Information 3 to avoid loading the paper with more tables and figures. As such, we have a "fairer'' baseline comparator for our new EpiLPS tool. Results show that even when comparing EpiLPS with this non lagged, non real-time version of EpiEstim, EpiLPS is still outperforming EpiEstim. Except for Scenario 1, where results are more or less of a similar order of magnitude, EpiLPS is in general less biased with more accurate credible intervals that are close to nominal coverage. The non lagged version of EpiEstim still exhibits more bias on average and also strong undercoverage in some cases. We also added a new figure in Section 3.1 that clarifies the difference between reporting Rt at the window boundary and at the midpoint of the window for EpiEstim.

A related point re EpiEstim is that one of the apparent added value of the new method is that it can deal with overdispersion, unlike EpiEstim, however I could not see an obvious improvement of the method's performance over EpiEstim's in scenarios with more overdispersion. I suggest the authors comment on this.

- In Scenarios with more overdispersion (i.e. Scenarios 1-4) an obvious improvement of EpiLPS over EpiEstim appearing in Table 2 of the main manuscript (and also now in the additional simulations that report Rt at the midpoint of the window in Supplementary information 3, Table-2-Supp3) can be seen in the column of the coverage probability. In particular looking at Scenario 2, 3 and 4, respectively, EpiEstim exhibits severe undercoverage going as low as around 65%, 23% and 9% respectively. Such low numbers for EpiEstim are not present anymore when considering the other scenarios where overdispersion is less strong. As such, EpiLPS performance is even more apparent compared to EpiEstim in scenarios with stronger overdispersion, at least regarding coverage performance of credible intervals. We revised the text to make this clear in Section 3.2.

I would also like to have more details on how the performance indicators computed in table 2 are calculated – is this on average across the whole epidemic? Because it seems from figures as if EpiEstim has for example wider CrI in the early epidemic periods, but quite narrow in the late epidemics.

- Detailed formulas for the computation of the performance indicators in the simulation study are now given in Supplementary Information 2. These indicators are computed across the whole epidemic (from day 8 to the last day of the epidemic in the corresponding scenario). The estimates in the first week are not taken into account as only limited information is available in such an early phase.

My second concern is that this is not applicable in real-time. Although the authors acknowledge it, they don't really show the behaviour of the method would have if applied in real-time. Most of the recent applications of EpiEstim have been to track the transmissibility of SARS-CoV-2 in real-time, so the authors should 1) show how their method performs in real-time in an extended simulation study, or if not then more clearly that it SHOULD not be applied in real time and 2) if not aimed at real-time, more clearly highlight the contexts in which this would be a good method to use (e.g. for retrospective analyses of an epidemic).

- EpiLPS is by nature only a partially real-time method and SHOULD therefore not be considered as a tool to replace EpiEstim. We clarify this in a new section (Section 3.4), specifically dedicated for discussing real-time considerations and retrospective recommendations. Note that in the first revision round, we already implemented a small simulation study that shows

the behavior of EpiLPS if it would have been applied in real-time (cf. Section 6 of Supplementary Information 2, where EpiLPS is applied in a timely manner on rolling windows and compared with EpiEstim). We do not think that adding further simulations in that direction would add value to the paper as our aim is not to prove that EpiLPS has better performance than EpiEstim in real-time. Given the currently available simulation results (including the new simulations with comparisons against Rt reported on the midpoint of the smoothing windows), we believe that it is fair to say that EpiLPS is a more robust retrospective estimation method as compared to EpiEstim. As such, EpiLPS is a tool that complements EpiEstim for robust retrospective analysis and using both tools jointly during an epidemic outbreak gives the end user the best of both worlds.

At the moment without such practical application guidelines, this feels like a very theoretical exercise, and I don't know to what extent it is useful.

- The new practical applications guidelines in Section 3.4 now clearly highlight that EpiLPS is well-tailored for retrospective analysis and EpiEstim for real-time implementation.

My third worry is that although the theoretical foundations of this novel approach seem valid, it may feel a bit like a black box to the user. For example, the scaling of the covariance matrix is briefly mentioned in the main text but it's unclear to me whether it's up to the user to do this, and if so how. Without a vignette with more instructions on how to use this tool I wonder whether one could really use it in practice. Similarly, although sensitivity analyses are now presented to demonstrate the impact of changing the number of splines, I feel like the user is not given an easy rule of thumb that they could follow re what value they should choose.

- By default, the user does not need to worry about the scaling of the covariance matrix as the latter is automatically implemented if the condition mentioned in the recommendation (see Supplementary Information 2, Section 3) is satisfied. Regarding the splines, we recommend using at least 30 B-splines in the basis (this is what we also currently use as a default value). In the large number of times we applied the EpiLPS tool, we have not faced any issues with using a minimum of 30 B-splines. Of course, the user is free to use more and this will not harm inference as shown by our sensitivity analyses. Although a vignette is not yet available, there are already various sources available that document and illustrate the use of the EpiLPS package, namely: (1) the EpiLPS reference manual available on CRAN (https://cran.r-project.org/web/packages/EpiLPS/EpiLPS.pdf), (2) the README page on the GitHub repository (https://github.com/oswaldogressani/EpiLPS), (3) the website dedicated to the project (https://www.epilps.com/) and (4) the folder containing the algorithms to reproduce the results of the paper (https://github.com/oswaldogressani/EpiLPS-ArticleCode). The latter sources already provide a large number of guidelines and instructions to use the EpiLPS package. A vignette as described by the reviewer is planned as part of a software paper on EpiLPS.

A final comment is on the evaluation of the overdispersion parameter. Even though it is considered a nuisance parameter, and I understand it is not the primary objective of the method to estimate it, I would have liked to see how well it is re-estimated in the simulation study.

- To have a flavor of the performance of the overdispersion parameter estimate with EpiLPS, we present boxplots of the latter parameter (in log scale) for Scenarios 5-8 computed over $S=100$ simulated epidemics and add the true value of the overdispersion parameter (in log scale) for the sake of comparison. The associated R code and figure is available in the GitHub

repository associated to the paper. Results show a mild (downward) bias and dispersion around the target.

Overall, as currently written, I think it is unclear in what context this method may be useful, and if it would truly outperform EpiEstim if a fair comparison was undertaken, I therefore encourage the authors to further revise their manuscript to address these issues.

- We now show that EpiLPS outperforms EpiEstim in a fair comparison.

I also add some more minor comments below

P21 “calibrating different values of rho” should be “using different values of rho”?

- “calibrating different values of rho” is changed to “using different values of rho” on P21.

End of p22: I struggle to understand how coverage can be so similar between two methods whilst one has much wider credible intervals; can you explain? Is this because one is really biased?

- Indeed, this is due to a bias argument. A general pattern that appears in the simulation results (Table 2 and Table 3) is that the coverage probability for EpiEstim with a one day window is closer to the target nominal value (but is still outperformed by EpiLPS) as compared to weekly or three days windows. The reason why the coverage is similar between EpiLPS and EpiEstim (with a one day window) despite EpiEstim having wider credible intervals is because EpiEstim provides biased Rt estimates. This feature can easily be seen from the figures provided in Supplementary Information 2. When the time window becomes smaller, the Rt estimate provided by EpiEstim captures more variation than necessary (undersmoothing). As such, the Rt estimates are often biased “above” or “below” the target and the wide credible intervals compensate (but unfortunately not enough) to encompass the target Rt value.

End of P24 you should also talk about the “price to pay” with the new method namely non applicability in real time

- We clearly specify that EpiLPS is by nature only a partially real-time approach and explicitly acknowledge that EpiLPS is a (robust) retrospective tool and that it is more preferable to use EpiEstim for analysis in real-time (see Section 3.4).

Figure 3 actually suggests more precise estimates of EpiEstim in that scenario?

- Figure 3 shows results for Scenario 5 comparing the trajectories of EpiLPS (LPSMAP) in blue with the trajectories of EpiEstim with weekly sliding windows in green. There are a few blue curves hat exhibit more volatility around the target as compared to the green curves, but globally the patterns are similar. Reading the corresponding performance measures in Table 2, we see that this reflects in a lower bias in favor of EpiLPS (0.013 vs 0.061) but also a lower MSE in favor of EpiEstim (0.162 vs 0.232). Note however that EpiEstim has coverage probabilities below the nominal value by roughly six percentage points for 90% CI and 95% CI respectively, while EpiLPS has a much better coverage performance.

Last sentence of section 3.1 --> I think this is really the main advantage of the new method and should be highlighted a bit more in the intro and conclusion; but the non-real-time nature of the approach should also be more emphasized

- We now use this main argument early on in the manuscript (see Introduction p.5). The partial real-time nature is also more specifically highlighted (see Section 3.4) and we also repeat the statement that EpiLPS is less preferred than EpiEstim for real-time analysis in the conclusion.

Figure 6: would be useful to see the underlying data (hospitalisations) and add some discussions about implications of using a proxy for infections (with alpha being more severe than previous variants R is going to be overestimated at the transition between the two when using hospitalisations).

- We compute the epidemic curve for the hospitalization data and add the code and figure in the GitHub repository associated to the paper so the underlying data for Belgium, Denmark, Portugal and France can be clearly visualized. The overestimation or underestimation of Rt that can potentially arise due to a change in variants is actually a very interesting point. Currently, the serial interval (SI) of EpiLPS has to be adapted manually to account for different variants that can be more/less virulent. Extending EpiLPS to allow for smooth transition between variants is a potential future research topic and we mention this in the discussion (Section 4).

First sentence of the discussion, you say "during epidemic outbreaks" but I think that is a push since the method won't work in real-time. You should clarify what contexts and analyses this method will be useful for.

- Albeit being a partially real-time approach (as now clarified in Section 3.4), EpiLPS can be used retrospectively even during epidemic outbreaks. We have used EpiLPS during the COVID-19 pandemic to track the Rt of Belgium (at a national level and for different regions) and it gives us interesting insights about its evolution over time.

P31 "EpiLPS and EpiEstim both use data from the past to estimate the instantaneous reproduction number" --> I think that is incorrect, i.e. EpiLPS also uses data from the future. If you want to demonstrate otherwise you need to present results of a real-time analysis.

- This is true, EpiLPS also uses data from the future to estimate Rt except for the last day on the domain of the epidemic curve where it uses the current value and past values. We make this explicit in Section 3.4 and repeat it again in the discussion (Section 4).

P31 "The method of Cori looks back in time only as far as the width of the chosen time window" --> That is also incorrect, it uses data from before that time window as well (it considers individuals infected in that time window but potential infectors from before as well as during that window).

- We changed the sentence to "The method of Cori looks back in time only as far as the width of the chosen time window in terms of infected individuals" to be more specific.

P31 "over the entire domain of the epidemic curve", i.e. including the future, I think this is important to highlight.

- It is now explicitly highlighted in Section 4.

P31 "there is however no free lunch" --> you should also highlight limitations of EpiLPS here namely the non real time nature which presumably greatly limit its applicability

- We highlight that EpiLPS is less preferred than EpiEstim for real-time analysis in the discussion section (Section 4). See also the dedicated new section (Section 3.4) that gives a more detailed description of the retrospective and real-time use of EpiLPS. We do not believe that EpiLPS as a partially real-time approach has a limited applicability.

P32, sentence starting “on that side” --> again you haven’t shown that EpiLPS will work if you are trying to estimate Rt in real time which is a major limitation so please clarify here that it is an accurate method to capture the PAST evolution of Rt.

- We make clear that EpiLPS is a tool for robust retrospective estimation in Section 3.4, however we do not believe that a retrospective analysis implies that EpiLPS is a tool that has a major limitation in practice. Based on the extensive simulation results, it turns out that EpiLPS provides more robust estimates as compared to EpiEstim (at least from a retrospective point of view). Using EpiEstim with a ‘‘too wide’’ window can blur biologically meaningful variations in Rt (Gostic et al. 2020). If looking at epidemic data over past values near the current day (e.g. until the day before yesterday), EpiEstim could miss a meaningful variation in Rt e.g. a convex direction change just below 1 indicating rising infections that EpiLPS will typically not miss.

P32 when recommending to use LPSMALA for shorter epidemics, perhaps highlight that it performs better than LPSMAP?

- LPSMALA has certainly better coverage performance, but it is not obvious to say if LPSMALA outperforms LPSMAP in terms of Bias or MSE based on the simulation results. So, we prefer not to mention that.

P32 “EpiLPS might be more accurate than EpiEstim in presence of overdispersed epidemiological data” --> what about in the absence of overdispersion? Again a detailed guide as to when one is best to use compared to the other would be really useful

- Actually, EpiLPS is more accurate than EpiEstim (for non real-time applications) in presence AND absence of overdispersion. In fact, Scenarios 5-8 (cf. Section 5 Supplementary Information 2) coincide with a virtual absence of overdispersion and EpiLPS has more accuracy compared to EpiEstim in the latter scenarios according to the reported performance indicators. We have added guidelines (Section 3.4) to make it clear that EpiLPS is robust for retrospective analyses.

Figure 11 supp2 and next ones: could you show how the CrI are affected by the changes in a_delta and b_delta parameters? This is important as it’s also one of the criteria you use to measure performance. Also in some of these figures the bias is actually quite a lot larger with low valus of a_delta and b_delta, which you should comment on in the matin text.

- We now show how the CI are affected by the changes in a_delta and b_delta (see additional code and figures given the in the GitHub repository associated to the paper). The CIs only vary slightly with a change of a_delta and b_delta, showing robustness. We added the following sentence below Figure-12-Supp2: “For $a_\delta = b_\delta = 5$ in Scenario 9, the median $R(t)$ trajectory is slightly more biased in regions where the target function has more pronounced curvature” to explicitly comment on the bias in this particular scenario.

Table 4 supp2 please explicitly say which assumptions (e.g. time window of EpiEstim) you are using.

- In Table-4-Supp-2 and in the text just above that table, we explicitly specify that we use weekly sliding windows for EpiEstim.

References

Eilers, P. H. C. and Marx, B. D. (1996). Flexible smoothing with B-splines and penalties. Statistical Science, 11(2):89–121. https://doi.org/10.1214/ss/1038425655

Cori, A., Ferguson, N. M., Fraser, C., and Cauchemez, S. (2013). A new framework and software to estimate time-varying reproduction numbers during epidemics. *American Journal of Epidemiology*, 178(9):1505–1512. https://doi.org/10.1093/aje/kwt133

Gostic, K. M., McGough, L., Baskerville, et al. (2020). Practical considerations for measuring the effective reproductive number, Rt. *PLoS Computational Biology*, 16(12), e1008409. https://doi.org/10.1371/journal.pcbi.1008409
