# Supplementary material for: EpiLPS: A fast and flexible Bayesian tool for estimation of the time-varying reproduction number
Source: PLoS Comput Biol. 2022 Oct 10;18(10):e1010618. doi: 10.1371/journal.pcbi.1010618 (PMC9584461; doi:10.1371/journal.pcbi.1010618)
Supplement: Attachment — Submitted filename: Answers_to_reviewers_Minrev.pdf [file pcbi.1010618.s006.pdf]

EpiLPS: a fast and flexible Bayesian tool for estimation of the time-varying reproduction number

Answers to reviewers' comments for Minor revision

General note to the reviewer

Dear reviewer,

I would like to thank you (on behalf of all co-authors) for your additional comments in this third review round. Indeed, adding the new simulation results (where the estimated Rt for EpiEstim is reported at the window midpoint) in the main text makes the comparison with the case where Rt is reported at the end of the window clearer. We also added the new sensitivity analyses to Supplementary Information 3 instead of including them exclusively in the GitHub repository and answered your minor comment. The point-by-point answers to your comments can be found below. Amendments made in the main manuscript are highlighted in color to ease the review.

Reviewer #1

I thank the authors for taking the time to take on board my main comment from the last round of review. Although I am happy with these new result, I am afraid I still have some comments as detailed below.

Why present the new results in supplementary material? I think these are the main results and should be shown in the main text, whilst the results at the end of the interval currently in section 3.2 should be transferred to supplementary material.

- The table showing the performance measures for the new results has now been moved from supplementary material to Section 3.3 and we also added 3 figures showing the estimated trajectories for Rt under Scenario 2, 3 and 9, respectively, when Rt is reported at the window midpoint for EpiEstim. The latter three figures can then be easily compared with the figures for the same scenarios in Section 3.2, where Rt is reported at the end of the window for EpiEstim. For the sake of space, we removed from the main text the figure associated with the scenario where Rt is constant over time as the latter is less interesting to analyze. Note however that the latter figure is still present in the supplementary materials.

The sensitivity analyses should also be altered to use this new window definition.

- Note that almost all sensitivity analyses are with respect to our EpiLPS approach and involves no sliding window. The only sensitivity analysis for which the new window definition could be adopted would be in Supplementary Information 2, where we measure the sensitivity with respect to the B-spline domain for EpiLPS and make comparison with EpiEstim (with weekly windows) for which the estimated Rt is reported at the end of the window (cf. Figure-15-Supp2). We now added the mean absolute error in Table-4-Supp-2 also for EpiEstim when the latter reports Rt at the window midpoint. EpiLPS still has lower mean absolute error values for most of the considered time domains.

The main text results show that the best performance of EpiEstim in terms of nominal coverage is often obtained for a 1d window, therefore the authors should add this to their new results for completeness (both tables and figures in the current supplement 3), and so the reader can fully assess the comparison between EpiEstim with the 3 time windows and EpiLPS.

- The performance measures and figures for the new results under a daily time window are actually identical to the results given in Section 3.2. In fact, under a daily time window $\omega = 0$, the sliding window under the Cori et al. (2013) convention [t-ω; t] and under the Gostic et al. (2020) convention [t-ω/2; t+ω/2] boils down to the degenerate interval [t,t]. As the simulations in Section 3.2 and 3.3 are using the same seeds (for the sake of comparison and reproducibility), the results are identical. This is the reason why we omitted the daily window results under the new simulations when Rt is reported at the window midpoint as it would be redundant and not informative. We add this explanation in Section 3.3 so that the reader can understand why results for daily windows are absent in Table 4. In addition, note that Table 1 in Supplementary Information 3 gives a detailed overview of how sliding windows are constructed under the two above-mentioned conventions.

Adding figures for new sensitivity analyses on the github repository, with no explanation and no legend, is in my opinion not sufficient, these should be included as supplementary material and appropriately documented inthere.

- We now added the new sensitivity analyses in Supplementary Information 3 as requested with appropriate documentation and figure captions. We also refer to the new sensitivity analyses in the main text (see last sentence in Section 3.5).

Minor:

Page 11 what do you mean by "(in)directly"?

- (In)directly was meant to refer to the fact that the estimated Rt depends on $\hat{\theta}$ via the "plug-in" estimate. Actually, we do not need the term (in)directly as it is quite explicit that Rt depends on θ (see for instance the beginning of Section 2.4.2). We therefore removed the term "(in)directly" on page 11 to make it less confusing.

Additional notes from the authors:

In figure captions, we give an explicit statement as to whether the estimated Rt for EpiEstim is reported at the end of the window or at the window midpoint.

References

Cori, A., Ferguson, N. M., Fraser, C., and Cauchemez, S. (2013). A new framework and software to estimate time-varying reproduction numbers during epidemics. *American Journal of Epidemiology*, 178(9):1505–1512. https://doi.org/10.1093/aje/kwt133

Gostic, K. M., McGough, L., Baskerville, et al. (2020). Practical considerations for measuring the effective reproductive number, Rt. *PLoS Computational Biology*, 16(12), e1008409. https://doi.org/10.1371/journal.pcbi.1008409
